# *Phaeophleospora vochysiae* Savi & Glienke sp. nov. Isolated from *Vochysia divergens* Found in the Pantanal, Brazil, Produces Bioactive Secondary Metabolites

**DOI:** 10.1038/s41598-018-21400-2

**Published:** 2018-02-15

**Authors:** Daiani C. Savi, Khaled A. Shaaban, Francielly Maria Wilke Ramos Gos, Larissa V. Ponomareva, Jon S. Thorson, Chirlei Glienke, Jürgen Rohr

**Affiliations:** 1 Department of Genetics, Universidade Federal do Parana, Av. Coronel Francisco Heráclito dos Santos, 210. CEP, 81531-970 Curitiba, PR Brazil; 2 Department of Pharmaceutical Sciences, College of Pharmacy, University of Kentucky, Lexington, Kentucky 40536-0596 USA; 3 Center for Pharmaceutical Research and Innovation (CPRI), College of Pharmacy, University of Kentucky, Lexington, Kentucky 40536-0596 USA

## Abstract

Microorganisms associated with plants are highly diverse and can produce a large number of secondary metabolites, with antimicrobial, anti-parasitic and cytotoxic activities. We are particularly interested in exploring endophytes from medicinal plants found in the Pantanal, a unique and widely unexplored wetland in Brazil. In a bio-prospecting study, strains LGMF1213 and LGMF1215 were isolated as endophytes from *Vochysia divergens*, and by morphological and molecular phylogenetic analyses were characterized as *Phaeophleospora vochysiae* sp. nov. The chemical assessment of this species reveals three major compounds with high biological activity, cercoscosporin (**1**), isocercosporin (**2**) and the new compound 3-(*sec*-butyl)-6-ethyl-4,5-dihydroxy-2-methoxy-6-methylcyclohex-2-enone (**3**). Besides the isolation of *P. vochysiae* as endophyte, the production of cercosporin compounds suggest that under specific conditions this species causes leaf spots, and may turn into a pathogen, since leaf spots are commonly caused by species of *Cercospora* that produce related compounds. In addition, the new compound 3-(*sec*-butyl)-6-ethyl-4,5-dihydroxy-2-methoxy-6-methylcyclohex-2-enone showed considerable antimicrobial activity and low cytotoxicity, which needs further exploration.

## Introduction

Endophytes are recognized as microorganisms that for all or part of their lifetime colonize internal plant tissues^1^, and using recent definitions, we refer to the term endophytes only in context of the microorganism habitat, with no definition of function (Hardoim *et al*. 2015). Endophytes associated with medicinal plants have been shown to possess a high potential to produce new bioactive metabolites, which often are valuable for plant growth promotion and/or biological control^2,3^.

Cataloguing microorganisms from unexplored biomes is an effective strategy to discover new species, often also producers of new secondary metabolites. Our groups are interested to explore the biodiversity of endophytes from medicinal plants found in the Pantanal, a unique and little explored wetland region in Brazil^2,4,5^, with seasonal inundation enduring more than 200 days a year^6^. Only few plants are able to tolerate such long periods of flooding, among them *Vochysia divergens* Pohl, a medicinal plant used in folk medicine to treat respiratory and gastric diseases^7^, and producer of antimicrobial compounds^8,9^.

In our search for novel bioactive compounds, we isolated two endophytic strains from *V. divergens*, with characteristic of the genus *Phaeophleospora*. The genus *Phaeophleospora* Rangel^10^ is an anamorph of the *Mycosphaerellaceae* and it is based on *P. eugeniae*, which was isolated in Brazil from leaf spots of *Eugenia uniflora*. To date, most species of this genus have been associated with leaf spot diseases in various plants, e.g., *Myrtaceae, Polypodiaceae, Elaeocarpaceae, Plantaginaceae, Proteaceae* and *Apocynaceae*^11–14^. So far, only two *Phaeophleospora* species, *P. stramenti* and *P. eucalypticola*, were found as endophytes from *Eucalyptus* sp^11^ and *Eugenia uniflora*^15^, respectively. Despite the fact that the genus *Phaeophleospora* was described as early as 1916, there were no reports of any bioactive secondary metabolites, and its metabolic potential remained unknown.

Based on morphological and phylogenetic analyses we describe here *Phaeophleospora vochysiae* as a new species within the genus *Phaeophleospora*, studied its secondary metabolites, and linked these to biological activities.

## Results

### Strains Isolation and Identification

**Figure 1:**
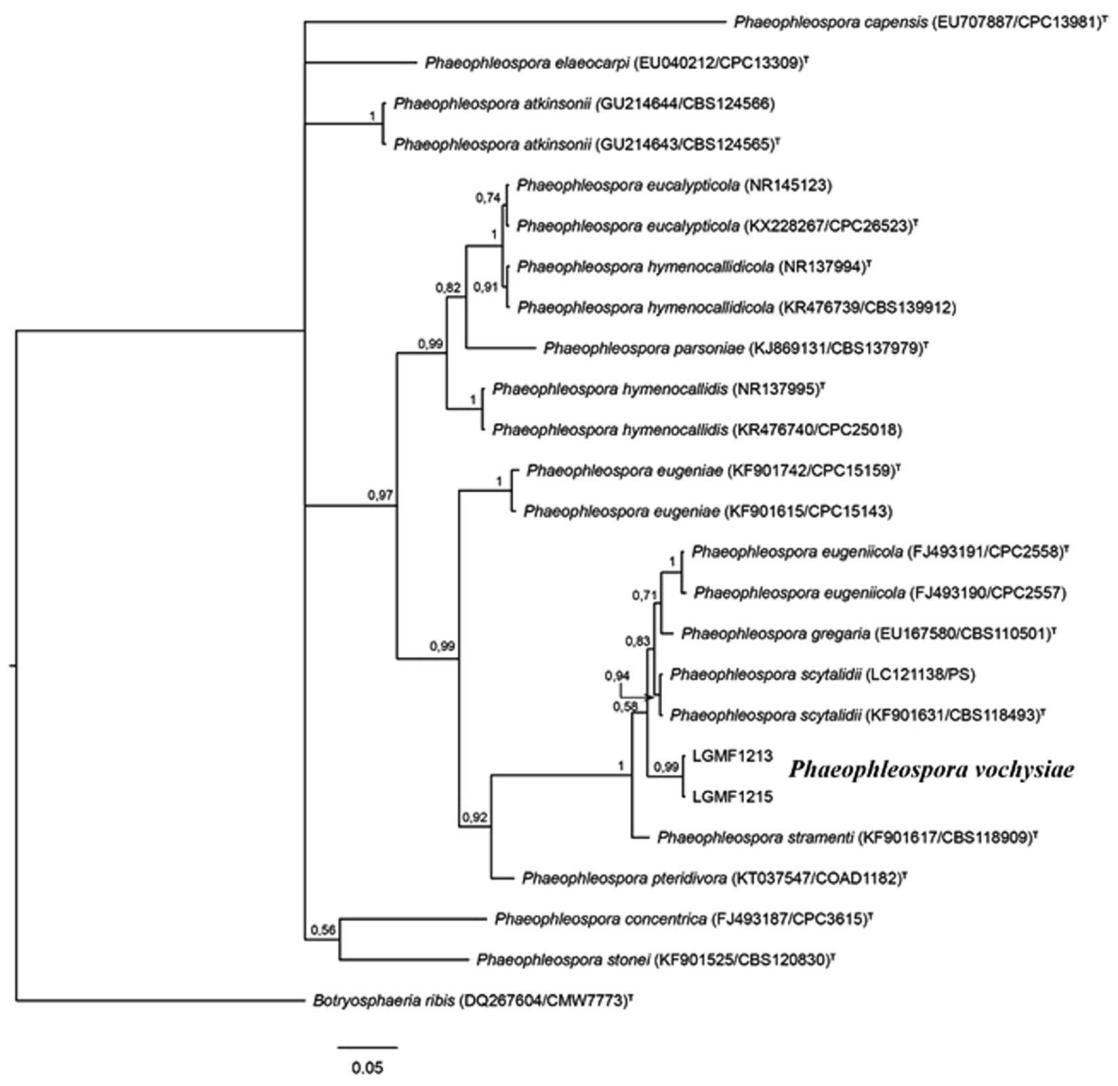
Bayesian phylogenetic tree based on ITS sequence of rRNA gene of LGMF1215 and LGMF1213 and 15 described species of *Phaeophleospora* genus. Values on the node indicate Bayesian posterior probabilities. The species *Botryosphaeria ribis* was used as outgroup. Scale bar indicates the number of substitutions per site.

Based on endophyte isolation protocol, strains LMGF1213 and LGMF1215 were isolated in June of 2010, from two different *V. divergens* asymptomatic plants, located in Nhecolândia, Pantanal, Brazil. The alignment of internal transcribed spacer region ITS1-5.8S-ITS2 comprised of 539 characters, 259 (48%) of these characters were constant, 122 (22.6%) were parsimony-uninformative and 158 (29.4%) characters were parsimony informative (Genbank access number: KY754582 and KY754582). The Bayesian phylogenetic analysis of ITS region showed strains LGMF1213 and LGMF1215 are in the same clade as *P. eugeniicola, P. gregaria, P. scytalidii* and *P. stramenti*, with a strong probability support (1.00), sharing 99% of ITS1–5.8S–ITS2 rDNA sequence similarity with species *P. scytalidii* (GenBank LC121138, Identities = 463/467). However, isolates LGMF1213 and LGMF1215 are located in a single branch (supported by 0.99 of probability), different from the *Phaeophleospora* species previously described (Fig. 1), and were characterized as a new species, *Phaeophleospora vochysiae* (Fig. 1). We also analyzed the large subunit ribosomal RNA (LSU) (Genbank access number: MG214701 and MG214702), elongation factor 1-alpha (TEF) (Genbank access number: MG190362 and MG190363) and acting (ACT) (Genbank access number: MG214703 and MG214704) genes (Fig. S38–40). As expected, LSU phylogenic analysis showed similar topology than ITS analysis, however with lower resolutions (Fig. S40). For the TEF gene, sequences of *P. eugeniicola, P. gregaria, P. scytalidii* and *P. stramenti* were not available, so our isolates clustered with the type species of the genus *Phaeophleospora* (*P. eugeniae*) (Fig. S39). ACT analysis that revealed strains LGMF1213 and LGMF1215 to be in the same clade as *P. eucalypti, P. destructans, P. gregaria* and *P. scytalidii*, however, in a separated branch (Fig. S38), supporting the proposition of LGMF1213 and LGMF1215 as a new species within the genus *Phaeophleospora*. A multilocus sequence analysis was not performed, since the sequence of other genes than ITS were not available for all the type strains.

### Taxonomic description

#### Phaeophleospora vochysiae

**Figure 2:**
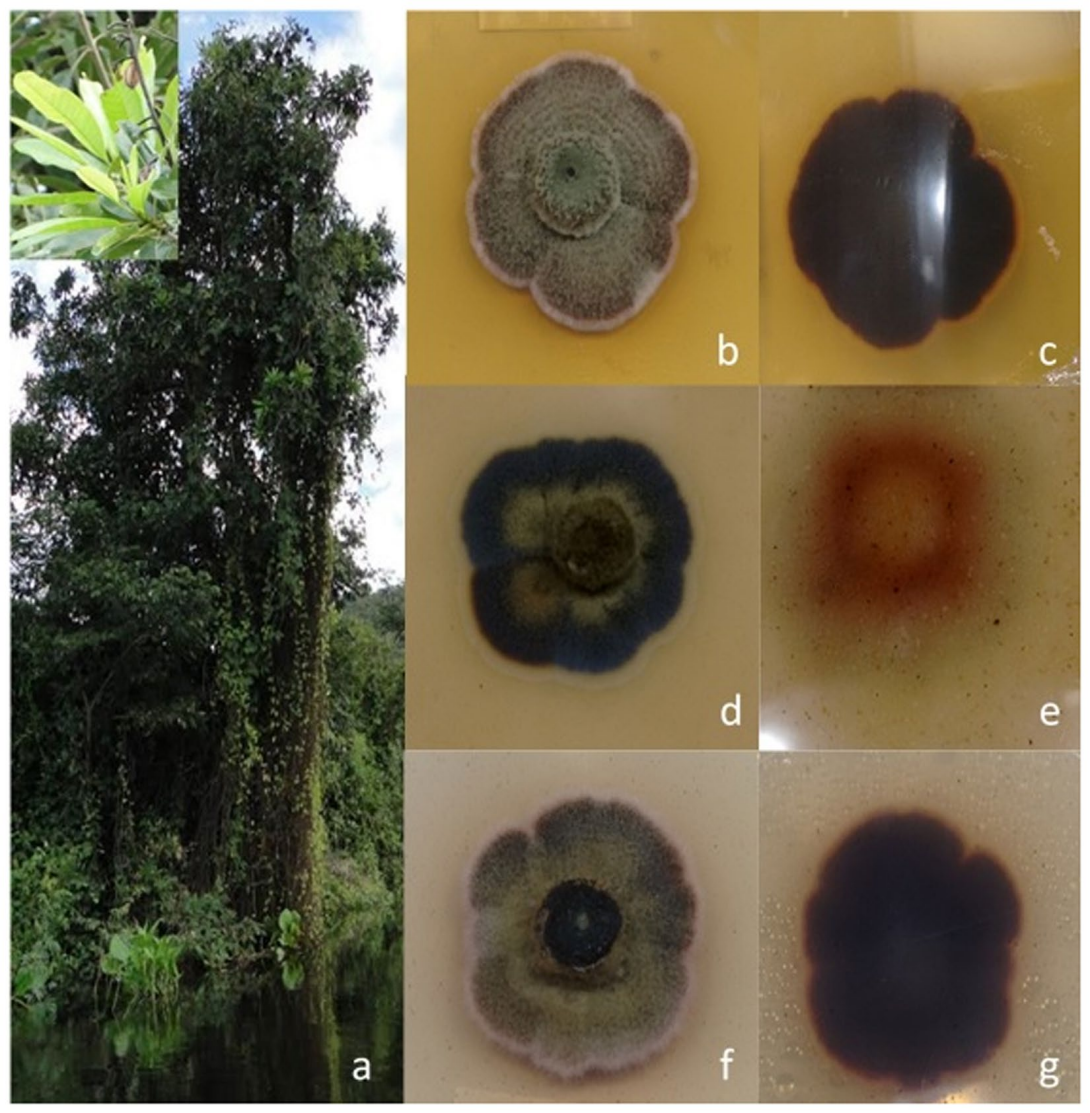
Host and morphology of *Phaeophleospora vochysiae* LGMF1215 on Malt Extract Agar (MEA), Oatmeal Agar (OA) and Potato Dextrose Agar (PDA) at 28 °C after 14 days. (**a**) *Vochysia divergens* (**b** and **c**) Colony on MEA surface and reverse; (**d** and **e**) colony on OA surface and reverse; (**f** and **g**) colony on PDA surface and reverse.

Savi & Glienke, *sp. nov*. Mycobank: MB821497. Fig. 2.

*Etymology*. Name after the host genus from which it was isolated, *Vochysia*.

**Figure 3:**
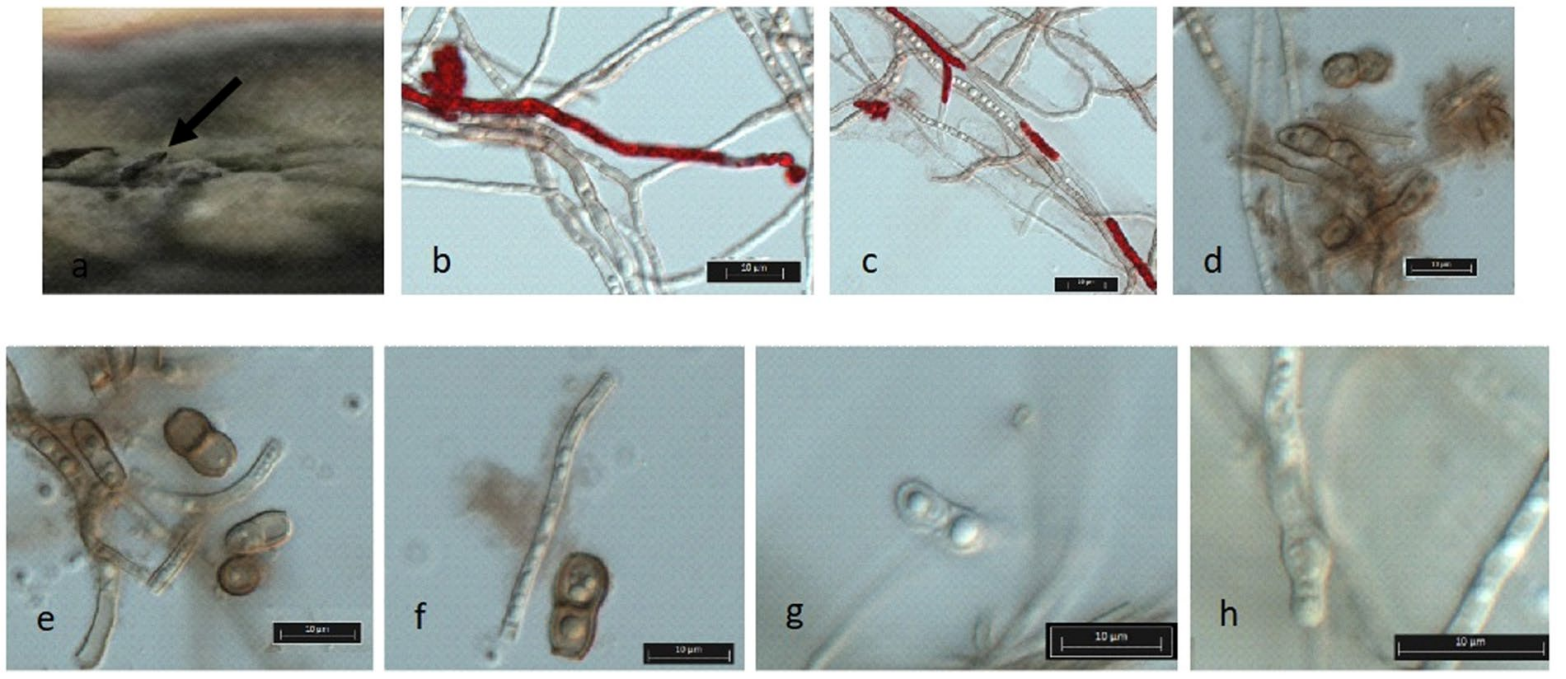
Macro- and micro-morphology of *Phaeophleospora vochysiae* LGMF1215. (**a**) Colony with pycnidium (arrow). (**b** and **c**) Mycelium consisting of septate, branched, verruculose hyphae in some points with red pigment inside. (**d**) Conidiophore and conidium. (**e**–**g**) Conidia. (**h**) Conidiophore.

*Mycelium* consisting of septate, branched, verruculose hyphae, 2–3 μm wide, in some points with red pigment inside, due the presence of cercosporin (**1**) and isocercosporin (**2**) (Fig. 3). Codiomata pycnidial, black, globose to subglobose. Conidiophores inside of pycnidium are aggregated arising from the upper cells of an irregular brown stroma up to 30 μm. Conidiogenous cells terminal, unbranched, medium brown, smooth to verruculose, 9.3 (7.4–11.2) × 5.0 (3.9–6.0) μm. Conidia solitary, or in simple chains, brown, verruculose, subcylindrical to oval, apex obtuse, base subtruncate, 1–2 septate, frequently constricted at the septa, 8.8 (7.0–13.7) × 4.7 (3.8–6.6) μm.

Culture characteristics: Colonies spreading, erumpent, lobed margins and moderate aerial mycelium, reaching 27 mm after 14 days at 28 °C. On Potato Dextrose Agar (PDA) medium, the surface is olivaceus-grey with vinaceus margin, the reverse side red due to a diffuse pigment. On oatmeal agar (OA) surface olivaceus-grey with yellow center, and on malt extract agar (MEA) green-grey and reverse iron-grey (Fig. 2).

Specimen examined: Brazil, Nhecolandia, Pantanal, Mato Grosso do Sul, S18°10.07′, W57°23.03′, on *Vochysia divergens* leaf, 9 Feb. 2010, D.C. Savi (Holotype: UPCB90660 (Herbarium of the Department of Botany code, University Federal of Paraná), ex-type culture LGMF1215 (Laboratory of Genetics of Microorganism Culture Collection code, University Federal of Paraná).

Notes: Strain LGMF1215 was isolated from healthy leaf tissue of *Vochysia divergens*, ITS sequence GenBank KY754582.

### Secondary metabolites

**Figure 4:**
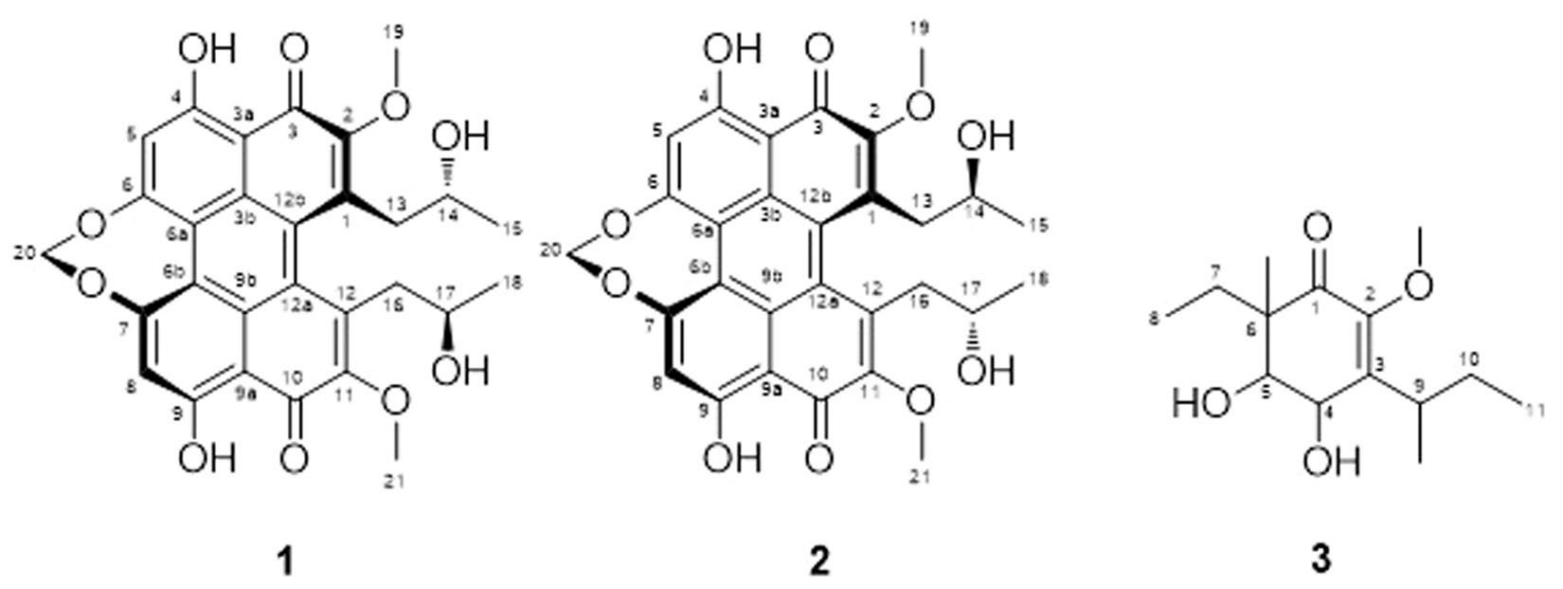
Chemical structures of compounds **1**–**3**.

A large-scale fermentation of the strain LGMF1215 in Potato Dextrose-medium produced 6.1 g of mycelium crude extract, and 3.7 g of culture filtrate extract. The purification of these extracts using various chromatographic techniques resulted two known compounds, (+)-cercosporin (**1**)^16,17^ and (+)-isocercosporin (**2**)^17^, plus one new compound, 3-(*sec*-butyl)-6-ethyl-4,5-dihydroxy-2-methoxy-6-methylcyclohex-2-enone (**3**) (Fig. 4 and S1). The chemical structure of compounds **1**-**3** were determined by cumulative 1D and 2D NMR spectroscopy, high resolution mass spectrometry (HRMS) (Table 1 and 2 and Fig. S2-S37), and by comparison with literature data^16,17^.

**Table 1:** Physicochemical properties of compounds 1–3 isolated from cultivation of strain LGMF1215.

	(+)-Cercosporin (1)	(+)-Isocercosporin (2)	Compound 3
Molecular Formula	C_29_H_26_O_10_	C_29_H_26_O_10_	C_14_H_24_O_4_
Appearance	Red solid, UV absorbing (254 nm)	Red solid, UV absorbing (254 nm)	Colorless oil, UV absorbing (254 nm)
2n NaOH	Dark-green	Dark-green	
HPLC-*R*_t_^a)^	21.45 (min)	21.85 (min)	18.19 (min)
(+)**-**APCI-MS: *m/z*	535 [M + H]^+^	535 [M + H]^+^	257 [M + H]^+^
(+)**-**HRESI-MS: *m/z*			
Found	535.1598 [M + H]^+^	535.1599 [M + H]^+^	257.1748 [M + H]^+^
Calcd.	535.1599 for C_29_H_27_O_10_ [M + H]^+^	535.1599 for C_29_H_27_O_10_ [M + H]^+^	257.1747 for C_14_H_25_O_4_ [M + H]^+^
(−)**-**HRESI-MS: *m/z*			
Found	533.1441 [M‒H]^−^	533.1444 [M‒H]^−^	255.1596 [M‒H]^−^, 291.1361 [M + Cl]^−^
Calcd.	533.1453 for C_29_H_25_O_10_ [M‒H]^−^	533.1453 for C_29_H_25_O_10_ [M‒H]^−^	255.1601 for C_14_H_23_O_4_ [M‒H]^−^ 291.1368 for C_14_H_24_ClO_4_ [M + Cl]^−^
UV/Vis λ_max_	220, 270, 450, 470 (sh), 570 (sh) nm	220, 270, 450, 470 (sh), 570 (sh) nm	250 nm

^a)^For HPLC, see Figs S4, S15 and S26.

**Table 2:** ^13^C (100 MHz) and ^1^H (400 MHz) NMR Spectroscopic Data of Compounds 1–3 in CDCl_3_ (*δ* in ppm) isolated from cultivation of strain LGMF1215, compared with the literature data.

Position	(+)-Cercosporin (**1**)		(+)-Isocercosporin (**2**)		Data reported in literature for compound **1**^**27**^	Data reported in literature for compound **2**^28^	Position	Compound **3**	
	*δ*_C_, type	*δ*_H_ (mult.)	*δ*_C,_ type	*δ*_H_ (mult.)	*δ*_H_ (mult.)	*δ*_H_ (mult.)		*δ*_C,_ type	*δ*_H_ (mult.)
1,12	135.4, C		136.8, C				1	198.5, C	
2, 11	153.0, C		153.4, C				2	148.1, C	
3, 10	182.0, C		181.9, C				2-OCH_3_	59.7, CH_3_	3.62 (s)
3a, 9a	108.4, C		108.6, C				3	144.7, C	
3b, 9b	128.1, C		127.6, C				4	68.8, CH	4.56 (m)
4, 9	167.7, C		167.8, C				5	74.4, C	3.87 (brd, 3.6)
4,9-OH		14.81 (s)		14.89 (s)	14.86 (s)	14.91 (s)	6	50.4, C	
5, 8	109.5, CH	7.06 (s)	109.4, CH	7.03 (s)	7.07 (s)	7.02 (s)	6-CH_3_	18.9, CH_3_	1.08 (s)
6, 7	163.6, C		163.6, C				7	25.4, CH_2_	1.81 (m), 1.73 (m)
6a, 6b	113.1, C		113.2, C				8	7.7, CH_3_	0.86 (t, 7.6)
12a, 12b	130.7, C		131.8, C				9	35.9, CH	2.80 (m)
13, 16	42.4, CH_2_	3.57 (dd, 13.0, 7.1) 2.88 (dd, 12.9, 5.9)	42.5, CH_2_	3.49 (dd, 14.0, 3.2) 2.85 (dd, 13.5, 8.4)	3.59 (dd, 13.1, 7.0) 2.90 (dd, 13.1, 6.0)	3.51 (dd, 13.3, 3.4) 2.87 (dd, 13.3, 8.2)	9-CH_3_	18.1, CH_3_	1.19 (d, 6.9)
14, 17	68.3, CH	3.36 (m)	69.8, CH	3.68 (m)	3.39 (m)	3.70 (m)	10	29.1, CH_2_	1.68 (m), 1.58 (m)
15, 18	23.6, CH_3_	0.62 (d, 6.0)	24.0, CH_3_	0.95 (d, 6.0)	0.65 (d, 6.1)	0.97 (d, 6.2)	11	13.1, CH_3_	0.91 (t, 7.5)
19, 21	61.4, CH_3_	4.19 (s)	61.2, CH_3_	4.21 (s)	4.21 (s)	4.23 (s)			
20	92.8, CH_2_	5.72 (s)	92.8, CH_2_	5.71 (s)	5.75 (s)	5.73 (s)			

*δ*_H_ (mult., *J* in [Hz]); Assignments supported by 2D HSQC and HMBC experiments.

The physicochemical properties of compounds **1–3** are summarized in Table 1. Compound **3** was obtained as colorless oil using various chromatographic techniques (see Supporting Information, Fig. S1). The molecular formula of **3** was deduced as C_14_H_24_O_4_ on the basis of (+) and (−)-HRESI-MS [*m/z* 257.1748 [M + H]^+^ (calcd. for C_14_H_25_O_4_, 257.1747) and 255.1596 [M‒H]^−^ (calcd. for C_14_H_23_O_4_, 255.1601)] and NMR data, revealing three double bond equivalents (DBE). The proton NMR spectrum of **3** in CDCl_3_ (Table 2) was rich in aliphatic proton signals, including two oxymethines (δ 4.56, m, 1 H; δ 3.87, brd, *J* = 3.6, 1 H), one methoxy (δ 3.62, s, 3 H), one methine (δ 2.80, m), two methylene (δ 1.85-1.50, 4 H), one singlet methyl (δ 3.62, s, 3 H) and four methyl signals of which two were observed as triplets at δ 0.91 (*J* = 7.5, 3 H) and 0.86 (*J* = 7.6, 3 H), suggesting the presence of two ethyl residues in **3**. The ^13^C NMR/HSQC spectra of **3** (Table 2) displayed 14 carbon signals corresponding to five methyl, two methylene, three methine and four quaternary carbon signals. In addition, the presence of three downfield quaternary carbon signals (δ 198.5, 148.1 and 144.7) indicate the presence of an *α*-*β*-unsaturated double bond with a carbonyl group (δ 198.5) in compound **3**. The attachment of the methoxy group at 2-position was confirmed through the ^3^*J* HMBC correlation observed from 2-OCH_3_ (δ_H_ 3.62) to C-2 (δ_C_ 148.1). The attachment of the ethyl/*sec*-butyl residues at 6-/3-positions, respectively, were established through the cumulative analyses of ^1^H^1^,H-COSY, TOCSY and HMBC spectra (Figures S2 and S3). The HMBC correlations observed from 6-CH_3_ (δ_H_ 1.08) to C-1 (δ_C_ 198.5), CH-5 (δ_C_ 74.4), C-6 (δ_C_ 50.4) and CH_2_-7 (δ_C_ 25.4) confirm the attachment of the methyl group at 6-position. All of the remaining HMBC correlations (Figure S2) and NMR data (Table 2) are in full agreement with structure **3**. Finally, the relative configuration at the stereocenters were indirectly established through the analyses of NOESY correlations (Figure S3), and by comparison to those of the reported cyclopentenone ring-contracted analogues, such as similin A (**4**) (produced by the fungus *Sporormiella similis*)^18^, phomapentenone A (**5**) (produced by the fungus *Phoma* sp. NRRL 25697)^19^ and boydone B (**6**) (produced by the plant associated *Pseudallescheria boydii* NTOU2362)^20^ (Figure S41). Thus, thorough cumulative analyses of 1D (^1^H^13^,C) and 2D (HSQC^1^,H^1^,H-COSY, TOCSY, HMBC and NOESY) NMR spectra established the structure of **3** as -(*sec*-butyl)-6-ethyl-4,5-dihydroxy-2-methoxy-6-methylcyclohex-2-enone (Fig. 4).

### Antibacterial and Antifungal Activities

The antimicrobial activity of the three crude extracts produced in the PD, Czapeck and MEA culture media were evaluated in order to select the best culture conditions to produce bioactive secondary metabolites. Extracts from PD showed the best results, with potent activity against the phytopathogen *Phyllosticta citricarpa*, moderate activity against methicillin sensitive and resistant *Staphylococcus aureus*, and low activity against the phytopathogen *Colletotrichum abscissum* (Table 3). The isolated compounds **1–3** (from cultures in PD medium), also displayed both, antibacterial activity against sensitive and resistant *S. aureus* and antifungal activity against the citrus phytopathogen *Phyllosticta citricarpa* (Table 3).

**Table 3:** Inhibition zones (in millimeters) of LGMF1215 crude extracts of different culture media and compounds 1~3 tested antibacterial and antifungal assays at 100 μg/disc.

Extracts/compounds	Staphylococcus aureus	MRSA	Escherichia coli	Phyllosticta citricarpa	Colletotrichum abscissum
Extract produced using PD	13	12	11	32	50
Extract produced using Czapeck	—	—	—	2	4
Extract produced using MEA	8	—	6	10	15
Cercosporin **1**	46	45	28	30	Ne
Isocercosporin **2**	35	27	20	28	Ne
3-(sec-butyl)-6-ethyl-4,5-dihydroxy-2-methoxy-6-methylcyclohex-2-enone **3**	14	14	—	30	Ne
Control	30	0	35	37	82

–denotes no measurable halo, antibacterial control: Ampicillin (1 mg/disc), Antifungal control: Derosal (1 mg/disc), ne: not evaluated.

### Cytotoxicity activity

**Figure 5:**
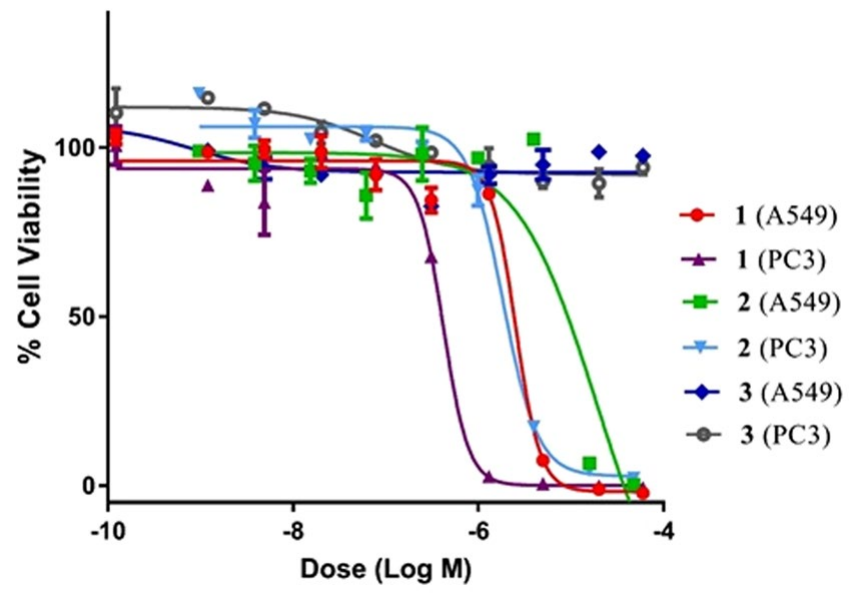
Dose-response viability assay of compounds **1**–**3** isolated from strain LGMF1215 against A549 (lung) and PC3 (prostate) human cancer cell lines for 72 hrs treatments. Note: each line represents a different treatment with a specific cell line and compound.

The cytotoxic activities of compounds **1–3** were determined, using PC3 (prostate) and A549 (non-small cell lung) human cancer cell lines (Fig. 5). Cell viability assays showed that cercosporin (**1**) and isocercosporin (**2**), were highly cytotoxic, IC_50_ = 2.82 μM (A549) and 0.42 μM (PC3), and IC_50_ = 19.21 μM (A549) and 2.10 μM (PC3), respectively. In contrast, compound 3-(*sec*-butyl)-6-ethyl-4,5-dihydroxy-2-methoxy-6-methylcyclohex-2-enone (**3**) showed no cytotoxicity up to 60 μM concentration against these cell lines.

## Discussion

Endophytic fungi are an important resource of secondary metabolites^5,21^. To better access this important source of bioactive molecules it is essential to explore the diversity of endophytes and to catalogue their species occurrence in different ecosystems^5^. Strains LGMF1215 and LGMF1213 were isolated from healthy leaves of a medicinal plant, *Vochysia divergens*, commonly found in flooding areas of the Pantanal (Brazil), and were characterized as a new species of *Phaeophleospora* genus, namely *P. vochysiae*.

Characterization of *Phaeophleospora* species traditionally was based on morphology^15^ in comparison with the type species *P. eugeniae*^10^. In the past, the asexual morphs of *Phaeophleospora* were characterized as pycnidial^22^, however, the recently described *P. pteridivora* has a sporodochial asexual morph^23^. Guatimosim *et al*.^23^ suggested that the genus *Phaeophleospora* should be defined based on phylogeny analysis instead, due to its high morphological diversity. So far, 19 species of *Phaeophleospora* were described, however, only 15 were accepted and validated based on ITS phylogenetic analysis (Table S1), but sufficient information to distinguish species within the genus *Phaeophleospora* was revealed^11,12,23,24^. We suggest a multilocus analysis as valuable tool to better understand evolutionary factors and intra- and inter-specific relationships of the *Phaeophleospora* species. We also suggest TEF1 and ACT as candidate genes for this analysis, in view of the number of informative sites compared to ITS and LSU sequences (Fig. 1, S-38-40). In the phylogenetic analysis, *P. vochysiae* is in the same clade (1) as *P. gregaria, P. scytaliddi, P. stramenti* and *P. eugeniicola*, but in a single branch, supported by high probability value (Fig. 1). Besides the phylogenetic support, *P. vochysiae* also showed difference in conidia size and growth rates from the *P. scytaliddi* (Table S2), the closest related species (99% of similarity in ITS sequence). *P. scytaliddi* has an elliptic conidium^25^, longer and narrowed compared to *P. vochysiae* and, different from the others species in this clade, only *P. vochysiae* produced a red pigment associated with the hypha (Fig. 3). *P. gregaria* also produced a red pigment in culture media, however it is not hypha associated^26^. The other species in the clade were associated with leaf spot diseases (Table S1), and, except for *P. gregaria*, which was described in Australia, they were all isolated in Brazil, from plants belonging to the *Myrtaceae* family^11,26^. This is the first isolation of a *Phaeophleospora* species from plants belonging to the family *Vochyseaceae* (*Vochysia divergens*). In contrast to those other species, the two strains of *P. vochysiae* were isolated from asymptomatic plants.

*Phaeophleospora* is an anamorph of the *Mycosphaerellaceae*^26^, and although other genera from the family of *Mycosphaerella* were recognized as producers of various secondary metabolites with antibacterial, antifungal and anti-parasitic activity^27–29^, there has been no report of secondary metabolites isolated from the species *Phaeophleospora*. Therefore, as a new species belonging to a genus without metabolite studies, and isolated from a region exposed to high hydric stress, we hoped for an interesting spectrum of secondary metabolites from *P. vochysiae*, including new metabolites. Two known compounds, cercosporin and isocercosporin, and a new compound, 3-(*sec*-butyl)-6-ethyl-4,5-dihydroxy-2-methoxy-6-methylcyclohex-2-enone, were produced in large amounts by the strain LGMF1215 (Fig. 3, S1). These compounds showed antibacterial activity against MSSA and MRSA, and high antifungal activity. Since MRSA strains have acquired resistance to many antibiotics and are associated with higher human death rate than those caused by HIV and influenza combined^30^, the search for new compounds with activity against this pathogen has great importance. In addition to the activity against MRSA, the new compound 3-(sec-butyl)-6-ethyl-4,5-dihydroxy-2-methoxy-6-methylcyclohex-2-enone (**3**) showed complete inhibition of mycelial growth of the phytopathogen *P. citricarpa*. On the other hand, compound **3** showed no cytotoxic activity against the human tumor cells evaluated, which may suggest a more selective, and less toxic antimicrobial activity (Table 3, Fig. 4). Therefore, compound **3** may be used for the control of the phytopathogen *P. citricarpa*, the causative agent of citrus black spot (CBS), a disease subjected to phytosanitary regulations in the European Union^31^.

The phytotoxic compounds cercosporin and isocercosporin are perylenequinones isolated for the first time in 1981 and 1991, from *Cercospora beticola* and *Scolecotrichum graminis*, respectively^16,17^, while 3-(sec-butyl)-6-ethyl-4,5-dihydroxy-2-methoxy-6-methylcyclohex-2-enone (**3**) was never found before, only similar cyclopentenone derivatives, i.e. ring-contracted analogues compared to **3**, such as similin A (**4**, produced by the fungus *Sporormiella similis*)^18^, phomapentenone A (**5**, produced by the fungus *Phoma* sp. NRRL 25697)^19^ and boydone B (**6**, produced by the plant associated *Pseudallescheria boydii* NTOU2362)^20^ (Figure S41). The cercosporins are photosensitizing compounds that interact with molecular oxygen to produce highly toxic singlet oxygen^32^. Nearly all bacteria and fungi are susceptible to cercosporin cell damage, however, the species of *Cercospora*^16,33^ and *Phaeophleospora vochysiae* are resistant. The resistance against cercosporins by *P. vochysiae* and the host *Vochysia divergens*, can be associated with different factors, the most common are the production of enzymes or compounds that quench or block the formation of oxygen species, such as carotenoids^34^ or antioxidants^35^. In addition, some studies suggest that cercosporin associated with the *Cercospora* hyphae is present in the reduced form, which makes the compound nontoxic, or not photoactived^36^. The association of this compound with the hyphae was also observed here in *P. vochysiae* (Fig. 3), and can be one explanation for the isolation of this species from healthy tissues from *V. divergens*. However, we cannot exclude the possibility of *P. vochysiae* to cause disease in certain unfavorable conditions.

In conclusion, the species *P. vochysiae*, isolated from healthy tissues of the medicinal plant *Vochysia divergens*, was described by morphological and phylogenetic analyses, and was characterized as a source of highly active secondary metabolites, including the new compound 3-(*sec*-butyl)-6-ethyl-4,5-dihydroxy-2-methoxy-6-methylcyclohex-2-enone (**3**). This new compound showed activity against MRSA and *P. citricarpa*, but no cytotoxicity. *P. vochysiae* also produced the phytotoxic compounds cercosporin (**1**) and isocercosporin (**2**) in culture conditions, suggesting the potential of this species to cause diseases, under specific conditions or in different host plants. More studies are necessary to find out whether the cercosporins are associated with the pathological mechanisms involved in the leaf spot diseases caused by other *Phaeophleospora* species, e.g., in *Eucalyptus* spp.

## Material and Methods

### Taxonomy

Strains LGMF1213 and LGMF1215 were isolated from *V. divergens* leaves collected in the Pantanal, in the region of Nhecolândia (S18°10.07′, W57°23.03′) in Brazil. For the isolation of endophytes, leaves with no marks, scratches or wounds were collected. To eliminate epiphytic microorganisms, a purification protocol of six steps was used^37^. The leaves were fragmented and inoculated in Petri dishes with medium PDA (Potato Dextrose Agar). The plates were incubated at 28 °C for 30 days, and the growth was verified daily. The cultures were deposited in the Laboratory of Genetics of Microorganisms (LabGeM) culture collection, Federal University of Paraná, Curitiba, Paraná, Brazil (http://www.labgem.ufpr.br/).

For the macro- and microscopic analysis, strain LGMF1215 was grown in plates in Potato Dextrose Agar (IBI Scientific), Oatmeal Agar (IBI Scientific) and Malt Extract Agar (Bacto Difco) culture medium at 23 °C and 28 °C, respectively, for 40 days, as described by Crous *et al*.^12^. Culture characteristics were studied from cultures 21 days after inoculation. Microscopic preparations were performed in distilled water, with 50 measurements per structure in microscope Axio Imager Z2 (Carl Zeiss, Jena, DE), equipped with Metafer 4/VSlide software (Metasystems, Altlussheim, DE), using differential phase interference contrast (DIC) illumination with software support, ImageJ.

Genomic DNA extraction was carried out using the UltraClean™ Microbial DNA Kit (MO Bio, Carlsbad, CA, USA). The internal transcribed spacer region (ITS) 1, 5.8 S, ITS2, LSU, elongation factor (TEF-1) and actin were amplified using the primers ITS4 and V9G^38^, LR0R and LR5^23^, EF-728F and EF-986R^23^, ACT-512F and ACT-783 R^18^, respectively. The PCR product was purified using the enzymes EXO1 and FastAP (ThermoFisher, Waltham, MA USA). The PCR product was sequenced using BigDye Terminator Cycle Sequencing Kit v3.1 (Applied Biosystems, Foster City, CA, USA), and sequences were analyzed on an ABI3500 DNA Sequencer (Applied Biosystems, Foster City, CA, USA). The sequence was compared with type specimens sequences available in the Genbank database of NCBI (http://www.ncbi.nlm.nih.gov/) and Mycobank (http://www.mycobank.org/), and aligned using CLUSTAL_X v.1.81^39^. Bayesian inference of the phylogeny was performed in MrBayes version 3.2.1^40^, with permutations allowed until a frequency of division ≤0.01 was reached. The general time-reversible (GTR) substitution model was used. FigTree version 1.4.2^41^ was used to edit the phylogenetic trees that were constructed. Sequences obtained in this study were deposited in GenBank with the accession numbers listed in Table S1.

### Fermentation, Extraction and Isolation

*Phaeophleospora vochysiae* LGMF1215 was cultivated on PDA-agar plates at 28 °C for 7 days. Fragments of agar (5/flask) were used to inoculate two Erlenmeyer flasks (500 mL) containing 250 mL of PD (IBI Scientific), MEA (Bacto Difco) and Czapeck media (Bacto Difco), and cultured for 21 days, at 27 °C and 250 rpm. Cultures were extracted with EtOAc (3 × 500 mL) and then the recovered organics were evaporated *in vacuo* at 40 °C. The antibacterial activity of crude extracts was determined and the best conditions were selected for a large-scale culture.

Large-scale fermentation (10 L) was performed using Potato Dextrose broth (IBI Scientific). The obtained red/black culture broth was separated by filtration using Whatman paper n 4. The biomass (mycelium) was extracted with MeOH/EtOAc (5 × 1000 mL) and then the recovered organics were evaporated *in vacuo* at 40 °C to yield 6.1 g of crude extract. The aqueous fraction was extracted with EtOAc (5 × 500 mL) and the recovered organics were evaporated *in vacuo* at 40 °C to yield 3.7 g of broth (culture filtrate) extract. The mycelium crude extract was subjected to Sephadex LH-20 (MeOH; 1 × 20 cm), and further purified by HPLC to afford compound **1** (3 mg) (Fig. S2). The EtOAc fraction was also subjected to Sephadex LH-20 (MeOH; 1 × 20 cm) and purified by HPLC to yield compounds **1** (2.6 mg), **2** (1.3 mg) and **3** (7.0 mg) in pure forms (Fig. S1).

### Antimicrobial and Antifungal Activity

The Gram-positive bacteria *Staphylococcus aureus* (ATCC 25923), methicillin-resistant *Staphylococcus aureus* (MRSA) (BACHC-MRSA) were maintained in lysogeny broth (LB) liquid media and Mueller-Hinton agar (Bacto Difco, USA). A loopful of each organism was inoculated into a 7 mL culture of LB broth and incubated in a 37 °C orbital shaker at 200 rpm for 10 hours. Each test organism was streaked on a sterile Mueller-Hinton agar plate with a sterile cotton swab. Compounds **1–3** were dissolved in methanol and aliquoted in 100 μg amounts per each 6 mm sterile filter disc and were allowed to dry in a laminar flow hood. The discs were placed on the plates, which were then incubated for 24 hours at 37 °C. The resulting inhibition zones were measured in millimeters.

The fungal strains *Phyllosticta citricarpa* LGMF06 and *Colletotrichum abscissum* LGMF1268 were used in disc diffusion assays. Solutions of amphotericin B (positive control) and tested compounds were dissolved in MeOH. Each sterile paper disc was loaded with 10 µL solution and was allowed to dry in the biosafety cabinet for 4 h. The dried discs were then placed on the PDA plate following the homogeneous distribution of fungus. MeOH was used as a negative control. The plates were incubated at 24 °C for 7 and 21 days, when the inhibition zones were measured.

### Cytotoxic activity

To assess the viability of human lung non-small cell carcinoma A549 and prostate adenocarcinoma PC3 cell against compounds **1**–**3** the conversion of resazurin (7-hydroxy-10-oxido-phenoxazin-10-ium-3-one) to its fluorescent product resorufin was monitored. DMEM/F-12 Kaighn’s modification media (Life Technologies, NY, USA) with 10% heat-inactivated fetal bovine serum (FBS), 100 U/mL penicillin, 100 μg/mL, streptomycin, 2 mM L-glutamine was used to grow A549 and PC3 cells (ATCC, Manassas, VA, USA). Cells were seeded at a density of 5 × 10^3^ cells per well in 96-well clear bottom culture plates (Corning, NY, USA), incubated 24 hours at 37 °C in a humidified atmosphere containing 5% CO_2_ and subsequently exposed to known toxins (1.5 mM hydrogen peroxide or 10 μg/mL actinomycin D, positive control) and test compounds for 72 hours. To assess cell viability, 150 μM of resazurin (Sigma, St. Louis, MO, USA) was added to each well, plates were shaken briefly for 10 seconds and incubated for another 3 hours at 37 °C to allow viable cells to convert resazurin into resorufin. The fluorescence intensity for resorufin was detected on a scanning microplate spectrofluorometer FLUOstar Omega (BMG Labtech, Cary, NC, USA) using an excitation wavelength of 560 nm and an emission wavelength of 590 nm.

## Electronic supplementary material

Supplementary information
