# Supplementary material for: Phaeophleospora vochysiae Savi & Glienke sp. nov. Isolated from Vochysia divergens Found in the Pantanal, Brazil, Produces Bioactive Secondary Metabolites
Source: Sci Rep. 2018 Feb 15;8:3122. doi: 10.1038/s41598-018-21400-2 (PMC5814415; doi:10.1038/s41598-018-21400-2)
Supplement: Supplementary file 1 — Supplementary information [file 41598_2018_21400_MOESM1_ESM.pdf]

Supporting Information

***Phaeophleospora vochysiae* Savi & Glienke sp. nov. Isolated from *Vochysia divergens* Found in the Pantanal, Brazil, Produces Bioactive Secondary Metabolites**

Daiani C. Savi [a,b,†], Khaled A. Shaaban[b,c,†], Francielly Maria Wilke Ramos Gos[a], Larissa V. Ponomareva[b,c], Jon S. Thorson[b,c], Chirlei Glienke[a*] and Jürgen Rohr[b*]

Department of Genetics, Universidade Federal do Parana, Av. Coronel Francisco Heráclito dos Santos, 210. CEP: 81531-970, Curitiba, PR, Brazil [a]; Department of Pharmaceutical Sciences, College of Pharmacy, University of Kentucky, Lexington, Kentucky 40536-0596, U.S.A.[b]; Center for Pharmaceutical Research and Innovation (CPRI), College of Pharmacy, University of Kentucky, Lexington, Kentucky 40536-0596, U.S.A.[c]

† Authors contributed equally to this work

* Correspondence authors: Jürgen Rohr, +1 859 323 5031, jrohr2@email.uky.edu; Chirlei Glienke, +5533611562, chglienke@gmail.com

Table of Contents:	Page
**General Experimental Procedures**	S2
**Physicochemical properties of compounds 1-2**	S3
**Figure S1.** Work-up scheme of the metabolites produced by *Phaeophleospora vochysiae* LGMF1215	S4
**Figure S2.** $^{1}H,^{1}H$-COSY (▬) and selected HMBC (↔) correlations in compounds **1-3**	S5
**Figure S3.** TOCSY (▬) and selected NOESY (↶↷) couplings in compounds **1-3**	S5
**Figure S4-37.** HPLC, UV, HRESI-MS and NMR spectra of compounds **1-3**	S6-39
**Figure S38.** Bayesian phylogenetic tree based on actin gene partial sequence of LGMF1213 and LGMF1215 and sequence of species of *Phaeophleospora* genus available in the Genbank. Values on the node indicate Bayesian posterior probabilities. The species *Botryosphaeria ribis* was used as outgroup. Scale bar indicates the number of substitutions per site.	S40
**Figure S39.** Bayesian phylogenetic tree based on elongation factor gene partial sequence of LGMF1213 and LGMF1215 and sequence of species of *Phaeophleospora* genus available in the Genbank. Values on the node indicate Bayesian posterior probabilities. The species *Botryosphaeria ribis* was used as outgroup. Scale bar indicates the number of substitutions per site	S41
**Figure S40.** Bayesian phylogenetic tree based on LSU gene partial sequence of LGMF1213 and LGMF1215 and sequence of species of *Phaeophleospora* genus available in the Genbank. Values on the node indicate Bayesian posterior probabilities. The species *Botryosphaeria ribis* was used as outgroup. Scale bar indicates the number of substitutions per site.	S42
**Figure S41.** Chemical structures of compounds **4-6**	S43
**Table S1.** Collection details and GenBank accession number of isolates included in this study	S44
**Table S2**. Morphological characters of isolate LGMF1215 and related *Phaeophleospora* species	S45

General Experimental Procedures

Ultraviolet-visible (UV-VIS) spectra were taken directly from analytical HPLC-PDA runs and show relative intensities. NMR spectra were measured using Varian (Palo Alto, CA) Vnmr 400 (^{1}H, 400 MHz; ^{13}C, 100 MHz) spectrometer where δ-values were referenced to respective solvent signals [CDCl3, δ_H 7.24 ppm, δ_C 77.23 ppm]. High resolution electrospray ionization (HRESI) mass spectra were recorded on AB SCIEX Triple TOF® 5600 system. HPLC-MS analyses were accomplished using a Waters (Milford, MA) 2695 LC module (Waters Symmetry Anal C18, 4.6 × 250 mm, 5 μm; solvent A: H_2O/0.1% Formic acid, solvent B: CH_3CN/0.1% Formic acid; flow rate: 0.5 mL min-1; 0-4 min, 10% B; 4-22 min, 10-100% B; 22-27 min, 100% B; 27-29 min, 100%-10% B; 29-30 min, 10 % B). HPLC analyses were performed on an Agilent 1260 system equipped with a photodiode array detector (PDA) detector and a Phenomenex C_{18} column (4.6 × 250 mm, 5 μm; Phenomenex, Torrance, CA). Semi-preparative HPLC was accomplished using Phenomenex (Torrance, CA) C18 column (10 × 250 mm, 5 μm) on a Varian (Palo Alto, CA) ProStar Model 210 equipped with a photodiode diode array detector and a gradient elution profile (solvent A: H_2O, solvent B: CH_3CN; flow rate: 5.0 mL min-1; 0-2 min, 25% B; 2-15 min, 25-100% B; 15-17 min, 100% B; 17-18 min, 100%-25% B; 18-19 min, 25% B). All solvents used were of ACS grade and purchased from the Pharmco-AAPER (Brookfield, CT). Size exclusion chromatography was performed on Sephadex LH-20 (25-100 μm; GE Healthcare, Piscataway, NJ). A549 and PC3 cells were obtained from ATCC (Manassas, VA). All other reagents used were reagent grade and purchased from Sigma-Aldrich (Saint Louis, MO).

Physicochemical properties of compounds 1-2

(+)-Cercosporin (1): Red solid; UV-absorbing (254 nm); dark-green with 2N NaOH; HPLC-R_t = 21.45 min (Figure S4); UV/vis λ_{max} 220, 270, 450, 470 (sh), 570 (sh) nm; ^{1}H NMR ($CDCl_3$, 400 MHz) and ^{13}C NMR ($CDCl_3$, 100 MHz), see Table S1; (+)-APCI-MS: *m/z* 535 $[M + H]^+$; (+)-HR-ESI-MS: *m/z* 535.1598 $[M + H]^+$ (calcd for $C_{29}H_{27}O_{10}$, 525.1599); (–)-HR-ESI-MS: *m/z* 533.1441 $[M – H]^–$ (calcd for $C_{29}H_{25}O_{10}$, 533.1453).

(+)-Isocercosporin (2): Red solid; UV-absorbing (254 nm); dark-green with 2N NaOH; HPLC-R_t = 21.85 min (Figure S15); UV/vis λ_{max} 220, 270, 450, 470 (sh), 570 (sh) nm; ^{1}H NMR ($CDCl_3$, 400 MHz) and ^{13}C NMR ($CDCl_3$, 100 MHz), see Table S1; (+)-APCI-MS: *m/z* 535 $[M + H]^+$; (+)-HR-ESI-MS: *m/z* 535.1599 $[M + H]^+$ (calcd for $C_{29}H_{27}O_{10}$, 525.1599); (–)-HR-ESI-MS: *m/z* 533.1444 $[M – H]^–$ (calcd for $C_{29}H_{25}O_{10}$, 533.1453).

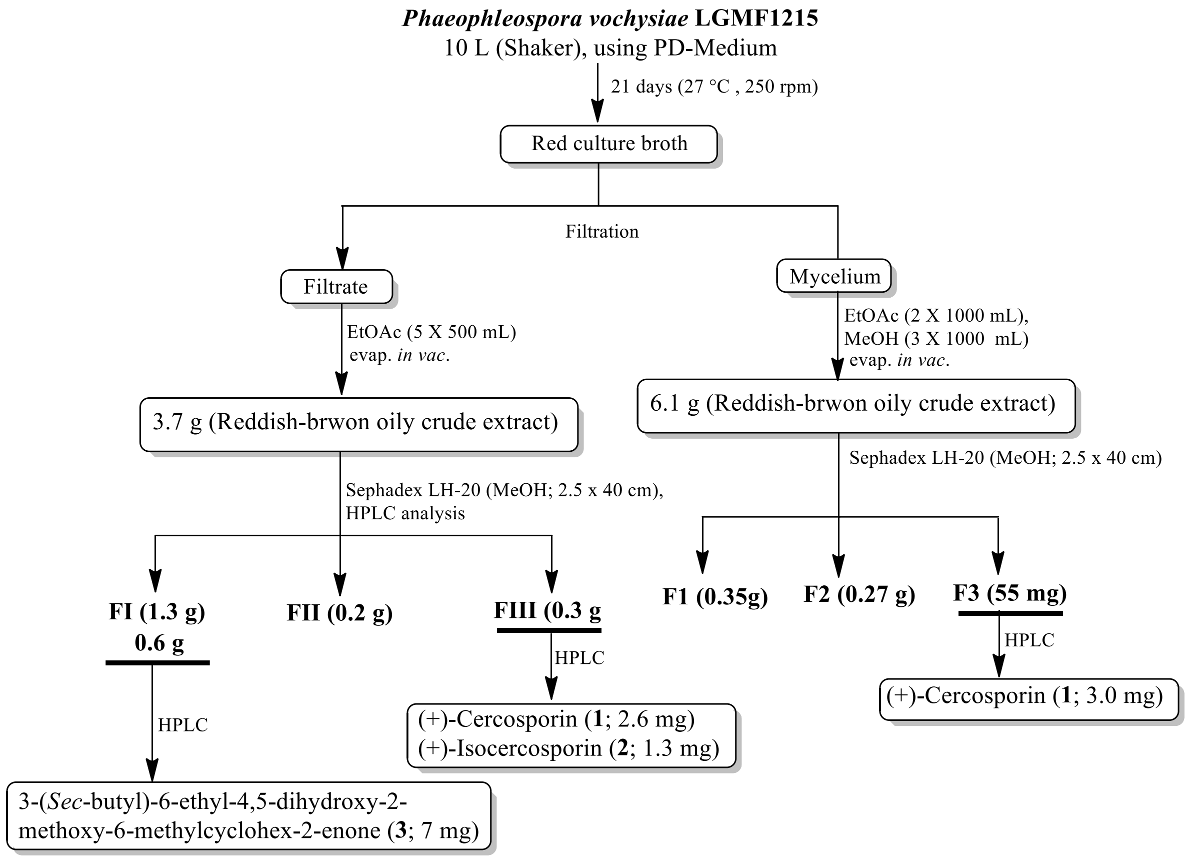

Figure S1. Work-up scheme of the metabolites produced by *Phaeophleospora vochysiae* LGMF1215

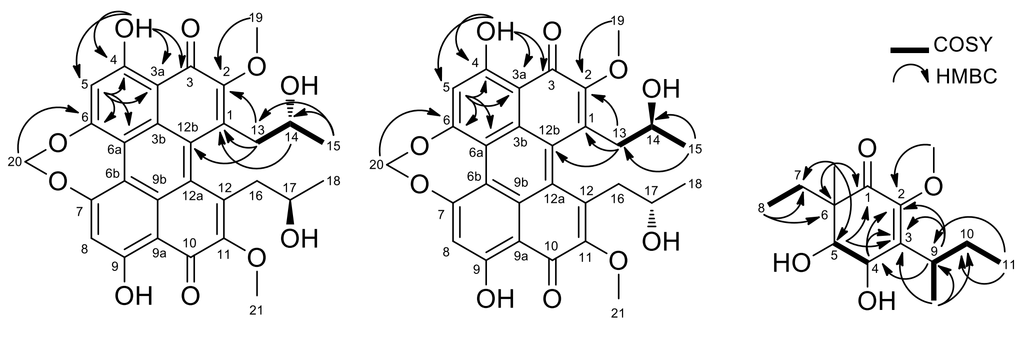

1 2 3

Figure S2. ^{1}H,^{1}H-COSY (▬) and selected HMBC (↔) correlations in compounds **1-3**

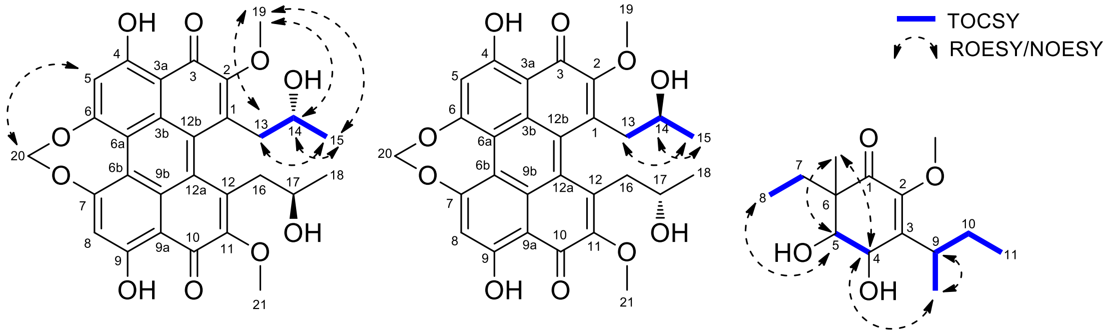

1 2 3

Figure S3. TOCSY (▬) and selected NOESY (↶↷) couplings in compounds **1-3**

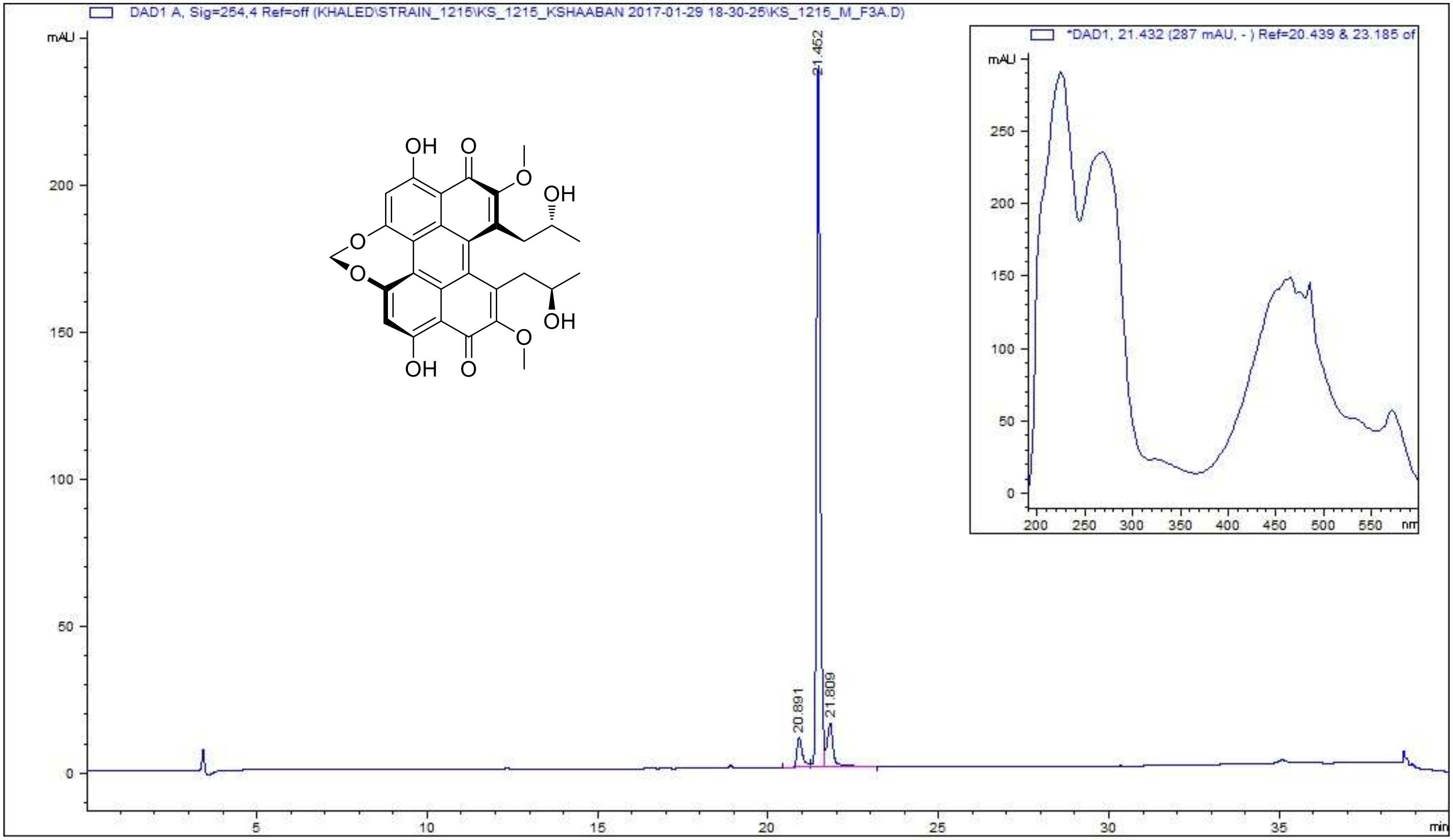

Figure S4. HPLC/UV analyses of (+)-cercosporin (**1**). HPLC-conditions: Detection wavelength 254 nm; solvent A: H_2O/0.1% TFA; solvent B: acetonitrile; flow rate: 1.0 mL min^{-1}; 0-35 min, 95-0% A (linear gradient); 35-40 min 0% A; 40-41 min 0-95% A (linear gradient); 41-45 min 95% A.

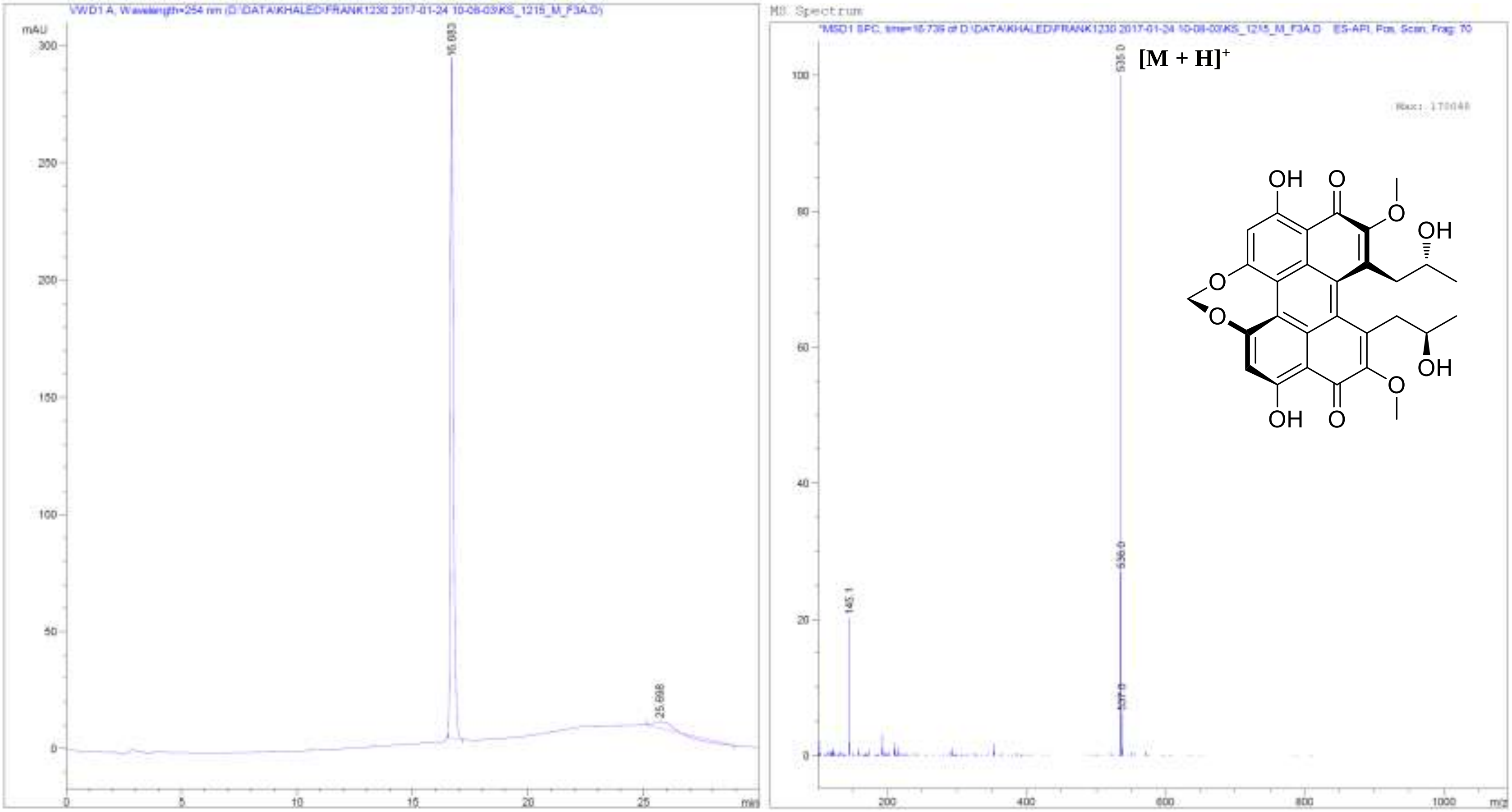

Figure S5. HPLC/MS analyses of (+)-cercosporin (**1**). HPLC-conditions: Detection wavelength 254 nm; solvent A: H_2O/0.1% Formic acid, solvent B: CH_3CN/0.1% Formic acid; flow rate: 0.5 mL min^{-1}; 0-4 min, 10% B; 4-22 min, 10-100% B; 22-27 min, 100% B; 27-29 min, 100%-10% B; 29-30 min, 10 % B.

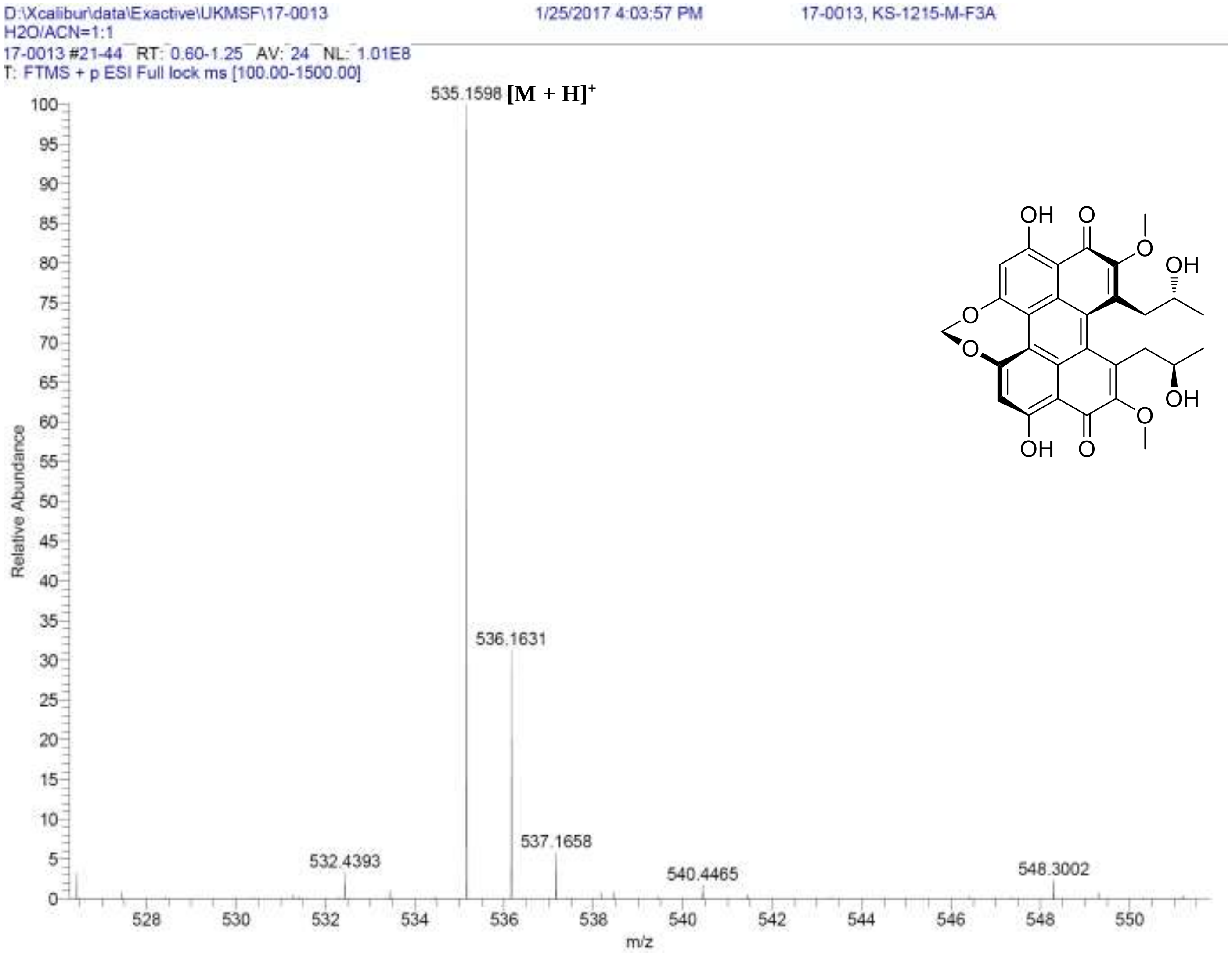

Figure S6. (+)-HRESI-MS spectrum of (+)-cercosporin (**1**)

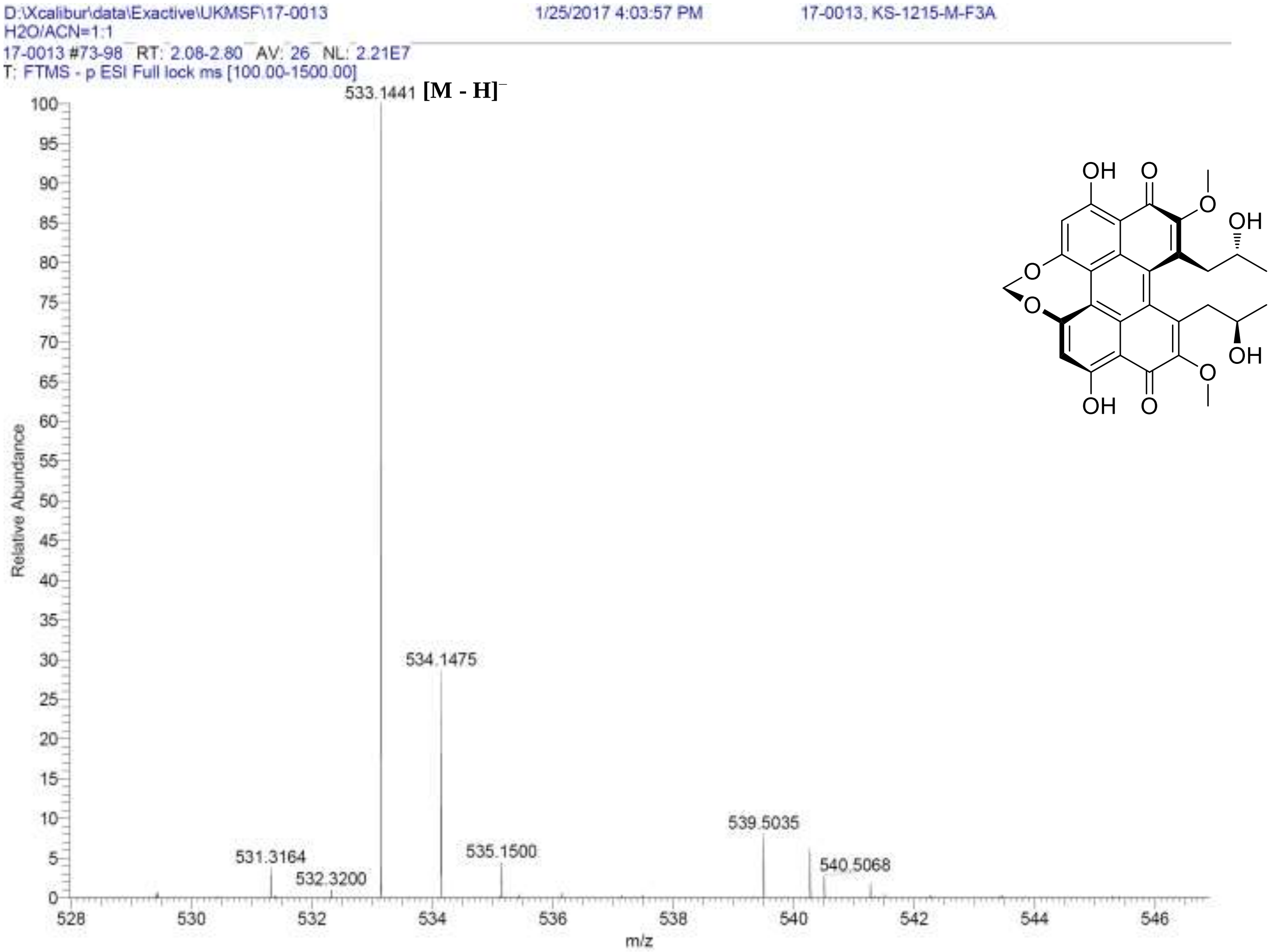

Figure S7. (−)-HRESI-MS spectrum of (+)-cercosporin (**1**)

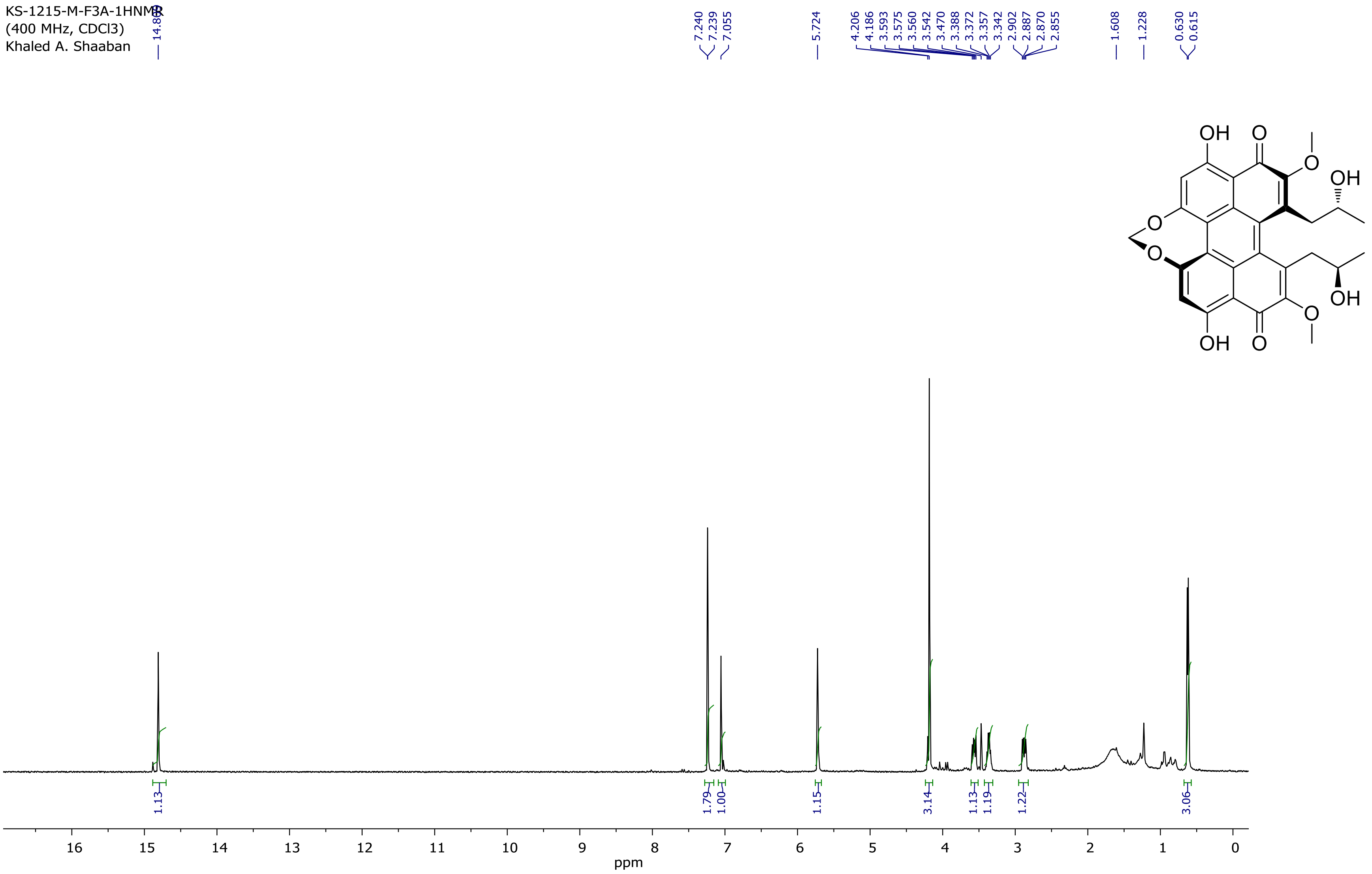

Figure S8. ^{1}H NMR spectrum ($CDCl_3$, 400 MHz) of (+)-cercosporin (**1**)

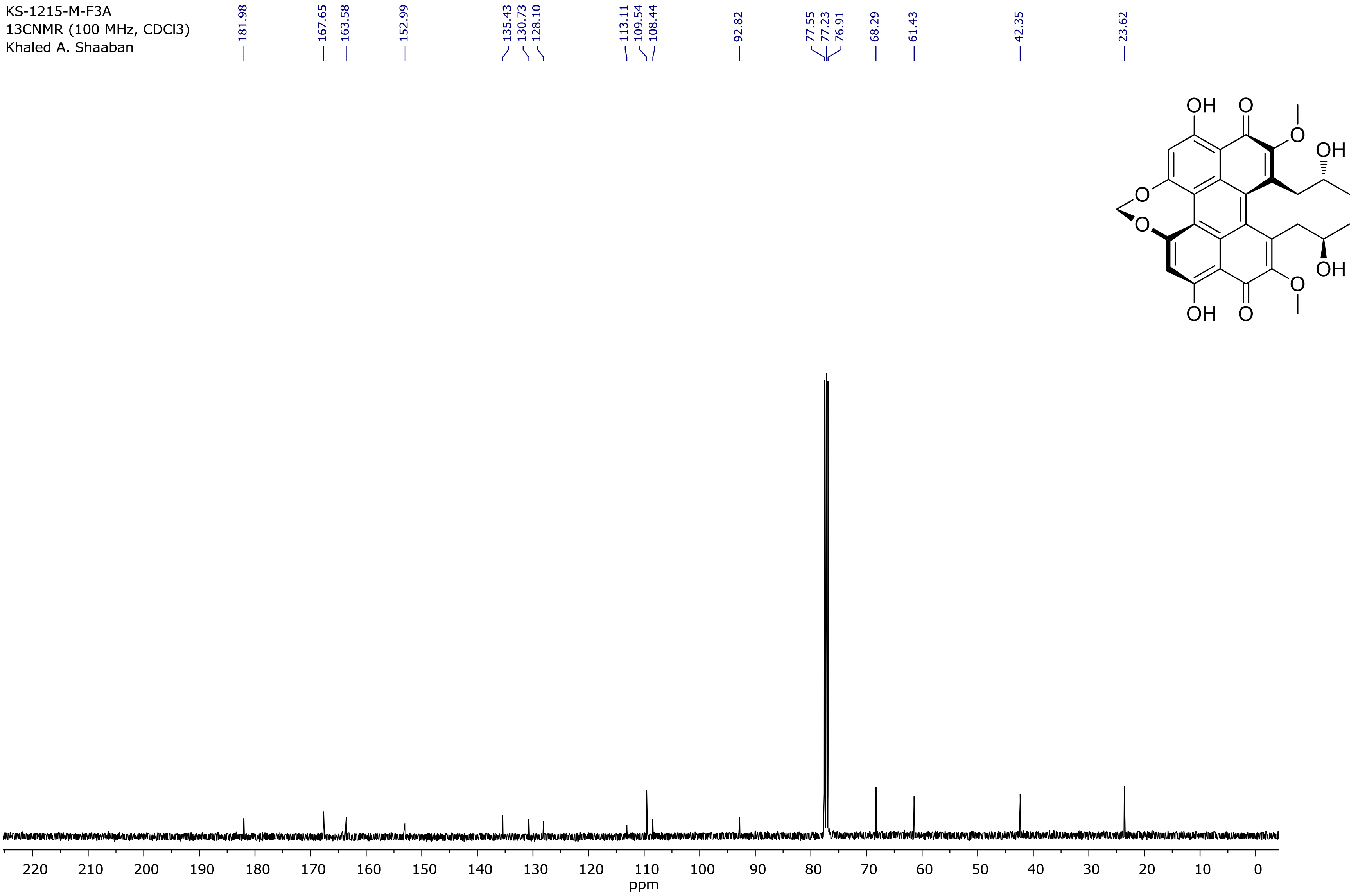

Figure S9. ^{13}C NMR spectrum ($CDCl_3$, 100 MHz) of (+)-cercosporin (**1**)

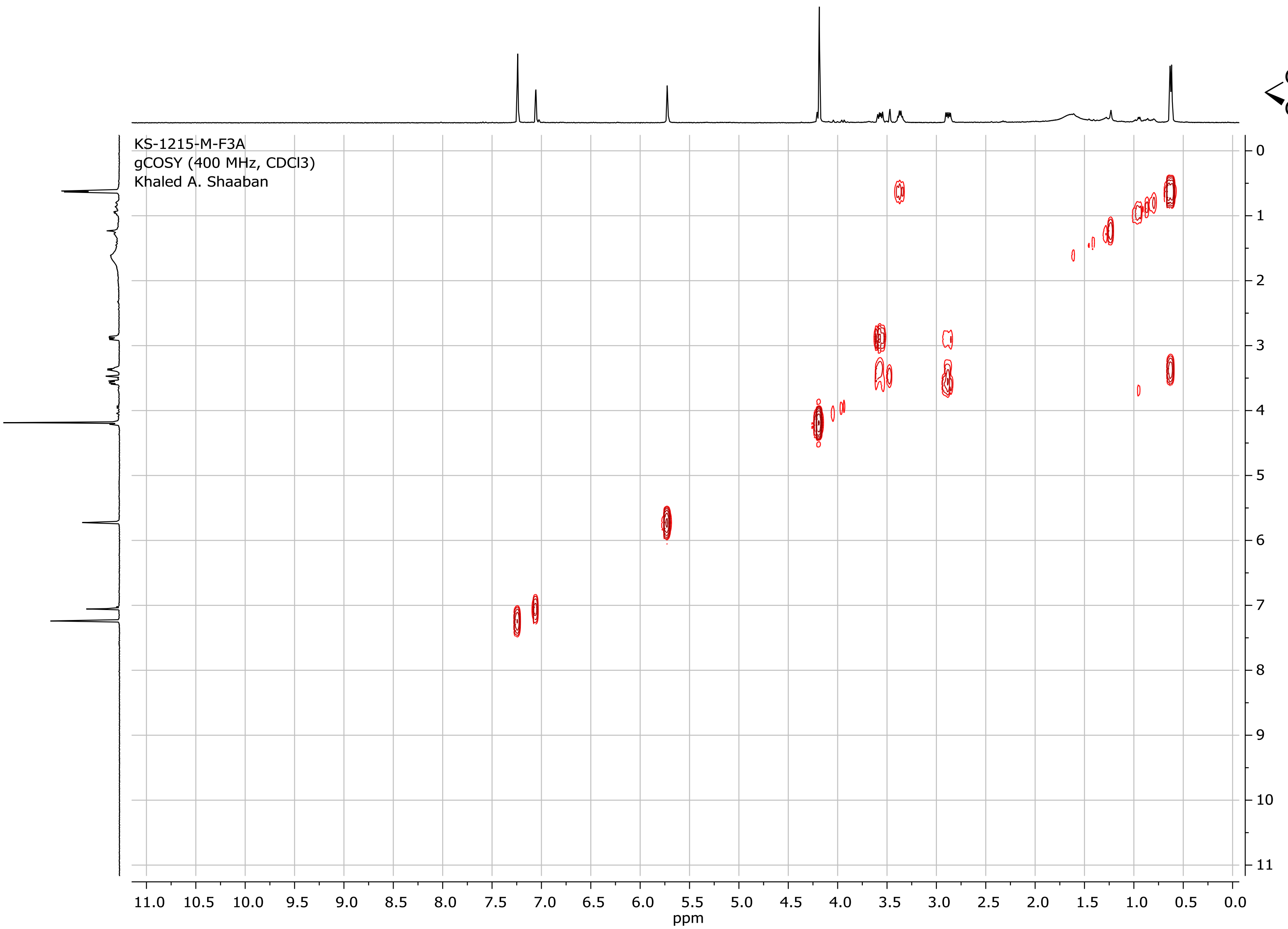

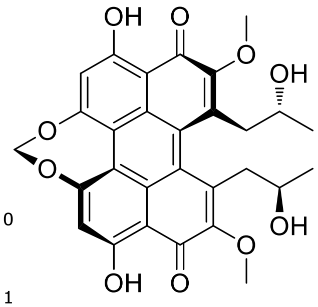

Figure S10. ^1H-^1H COSY correlation ($CDCl_3$, 400 MHz) of (+)-cercosporin (**1**)

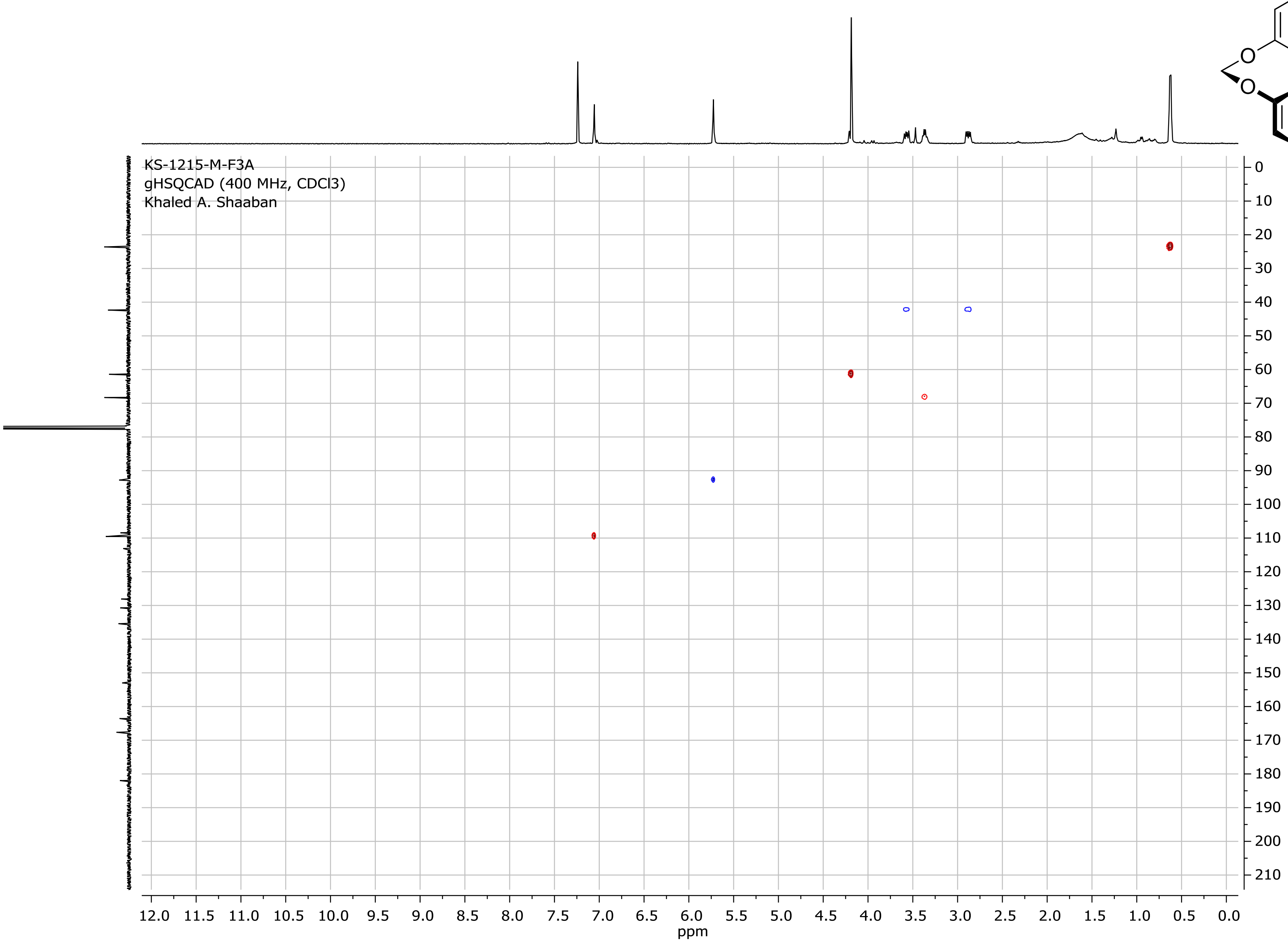

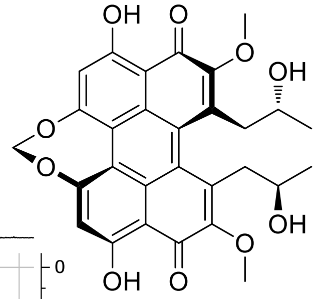

Figure S11. HSQC spectrum ($CDCl_3$, 400 MHz) of (+)-cercosporin (**1**)

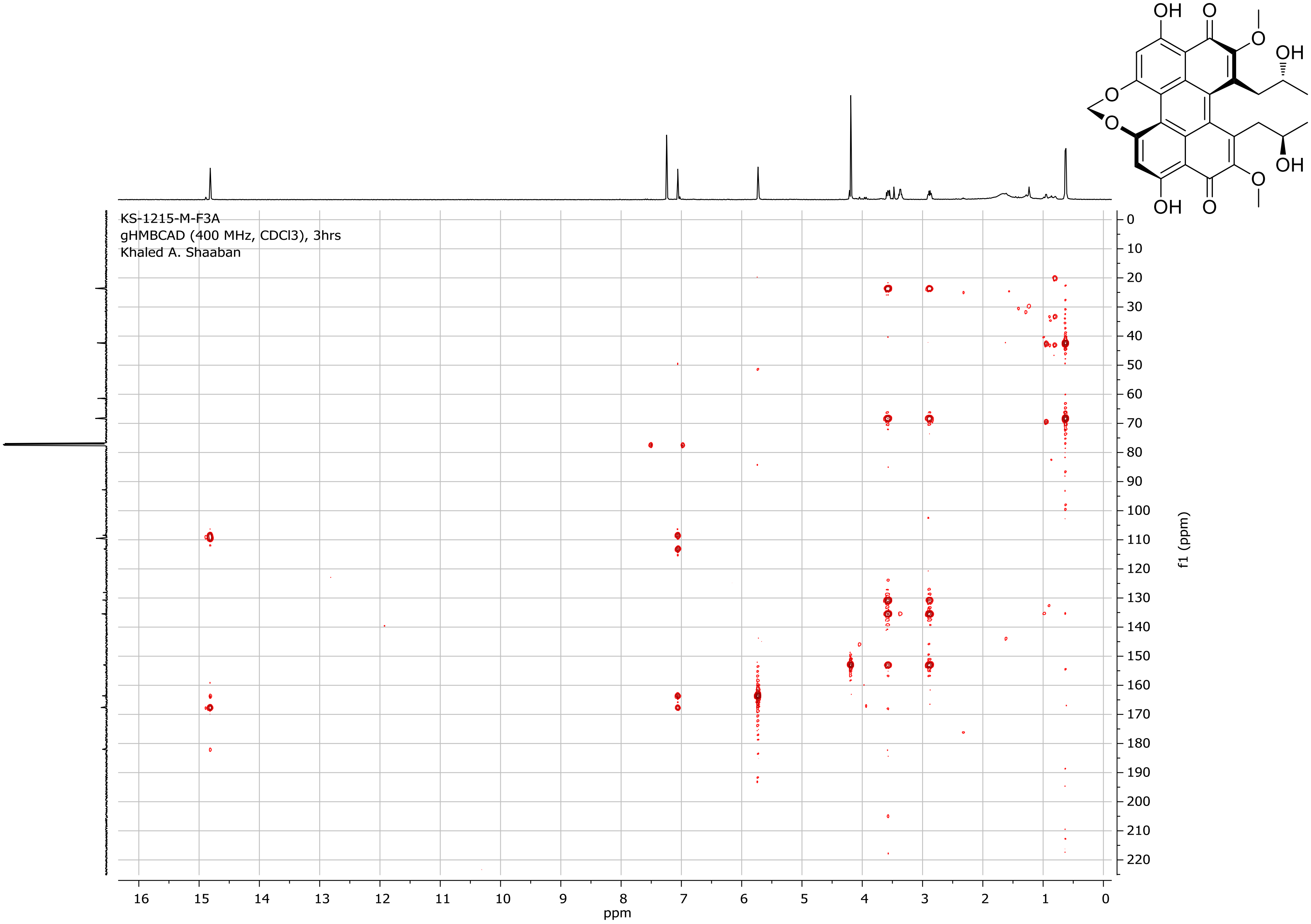

Figure S12. HMBC spectrum ($CDCl_3$, 400 MHz) of (+)-cercosporin (**1**)

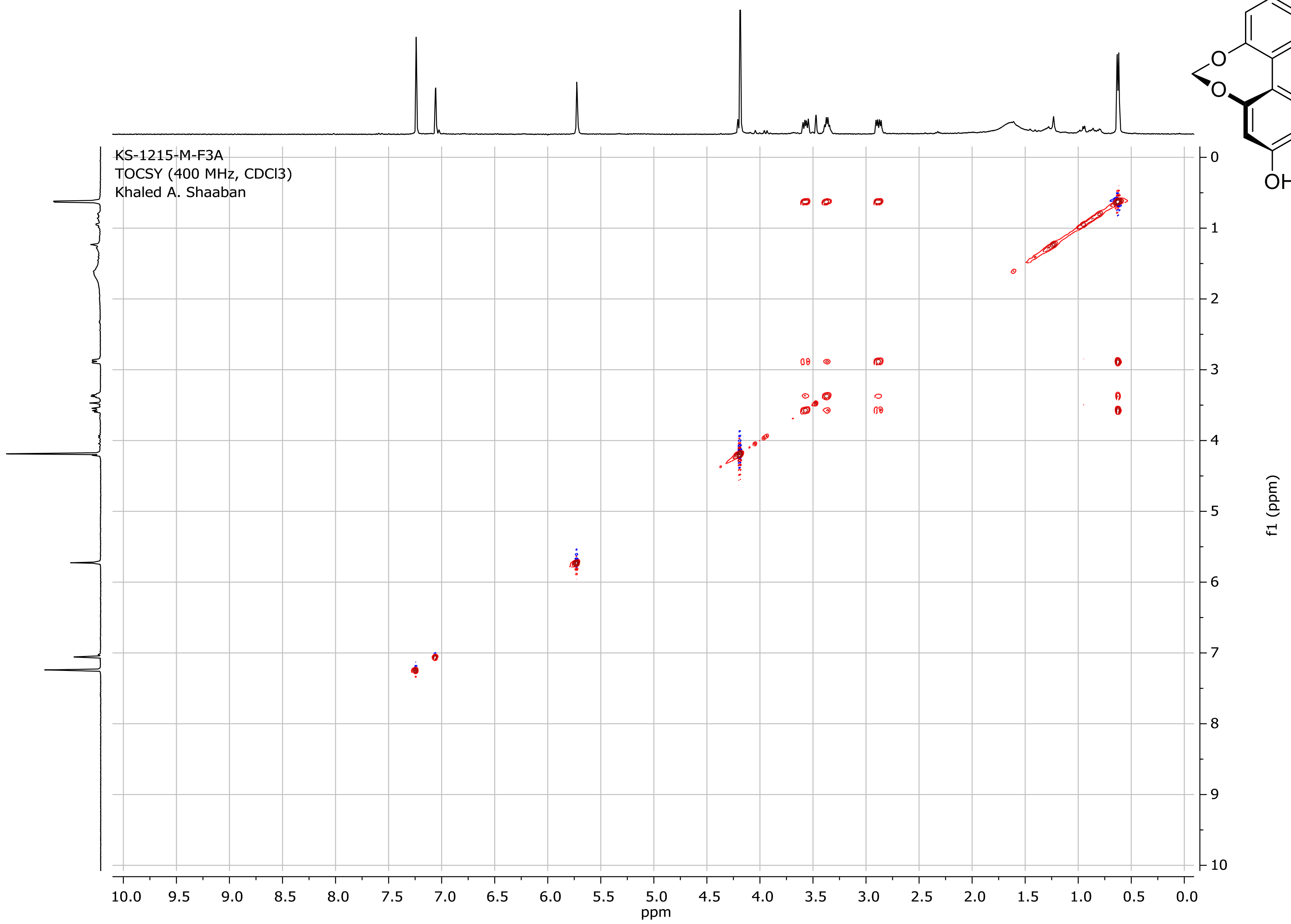

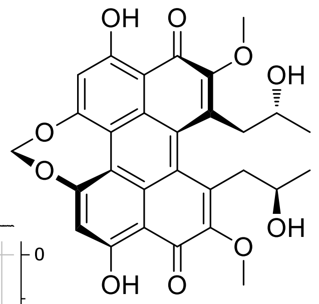

Figure S13. TOCSY spectrum ($CDCl_3$, 400 MHz) of (+)-cercosporin (**1**)

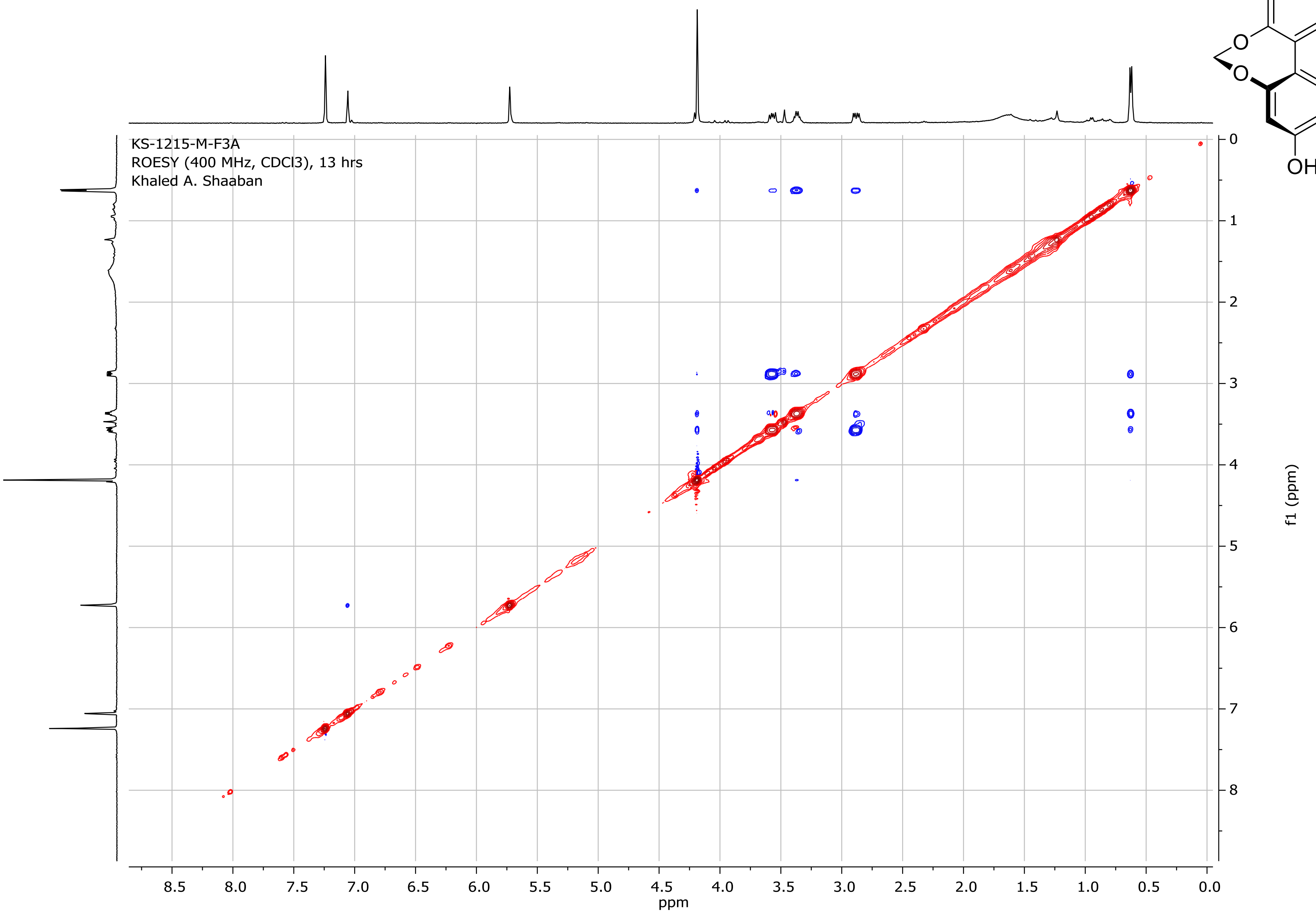

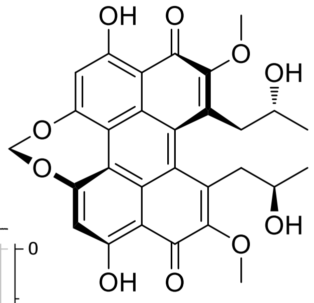

Figure S14. ROESY spectrum ($CDCl_3$, 400 MHz) of (+)-cercosporin (**1**)

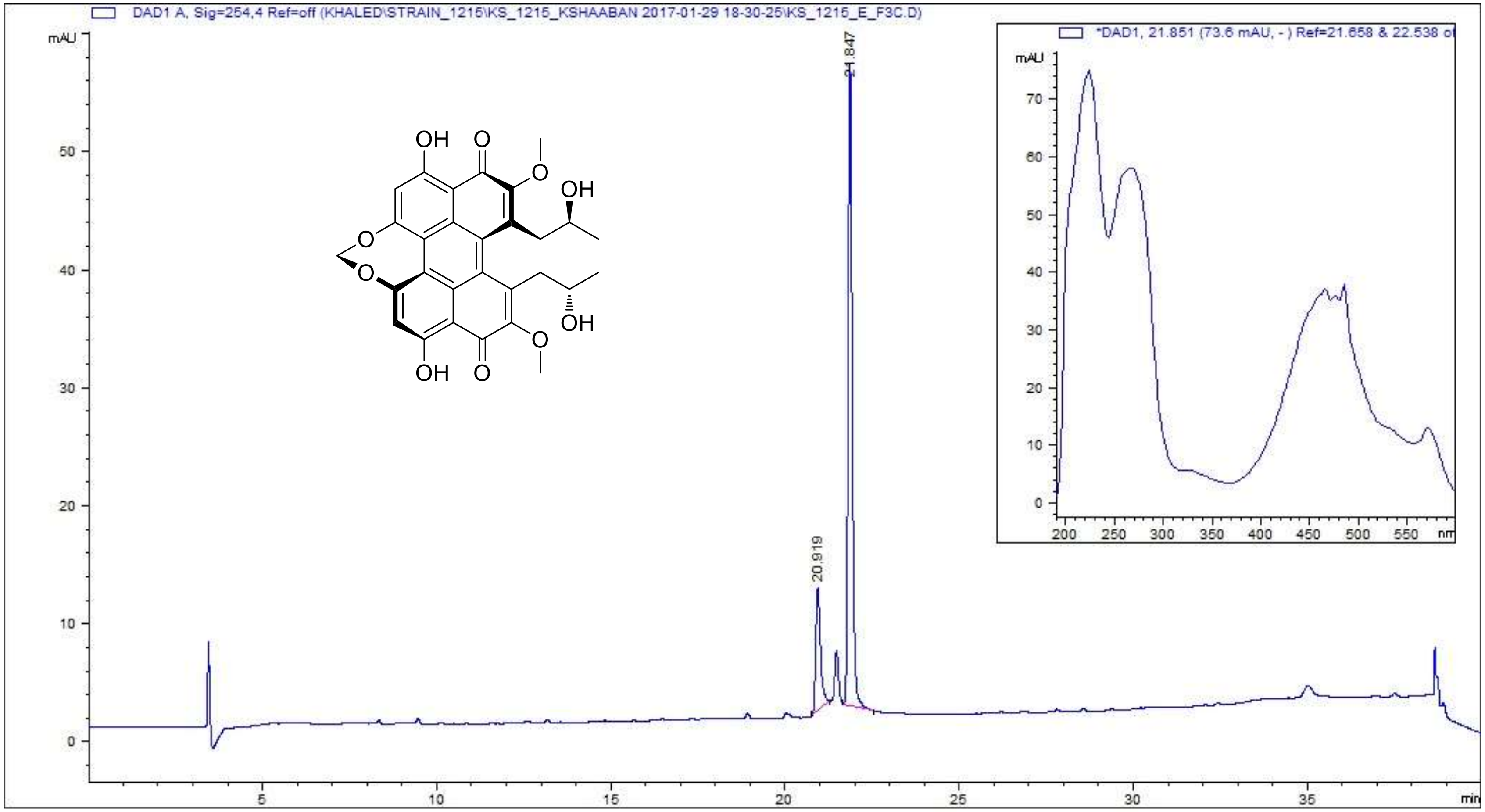

Figure S15. HPLC/UV analyses of (+)-isocercosporin (**2**). HPLC-conditions: Detection wavelength 254 nm; solvent A: H_2O/0.1% TFA; solvent B: acetonitrile; flow rate: 1.0 mL min^{-1}; 0-35 min, 95-0% A (linear gradient); 35-40 min 0% A; 40-41 min 0-95% A (linear gradient); 41-45 min 95% A.

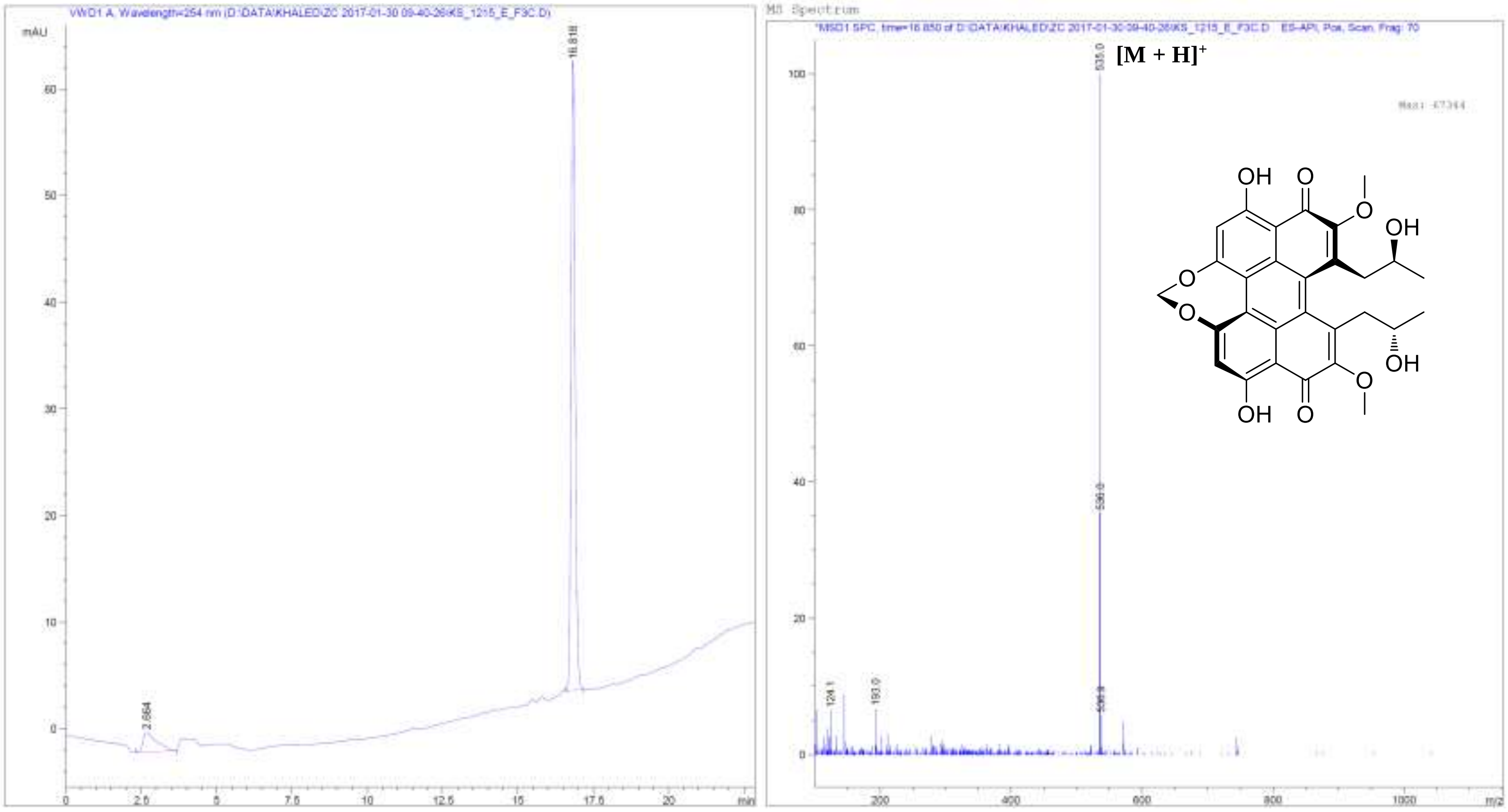

Figure S16. HPLC/MS analyses of (+)-isocercosporin (**2**). HPLC-conditions: Detection wavelength 254 nm; solvent A: H_2O/0.1% Formic acid, solvent B: CH_3CN/0.1% Formic acid; flow rate: 0.5 mL min^{-1}; 0-4 min, 10% B; 4-22 min, 10-100% B; 22-27 min, 100% B; 27-29 min, 100%-10% B; 29-30 min, 10 % B.

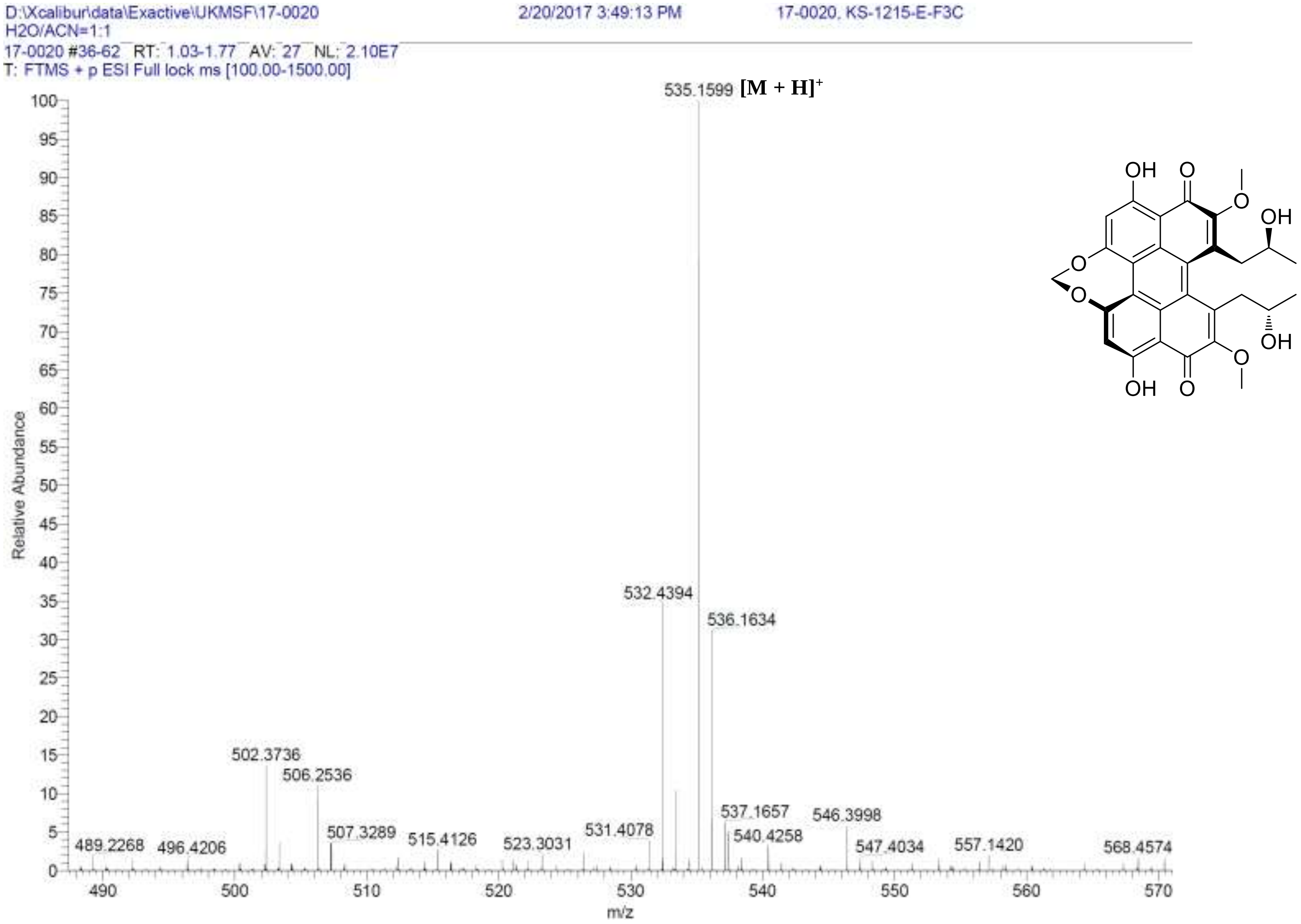

Figure S17. (+)-HRESI-MS spectrum of (+)-isocercosporin (**2**)

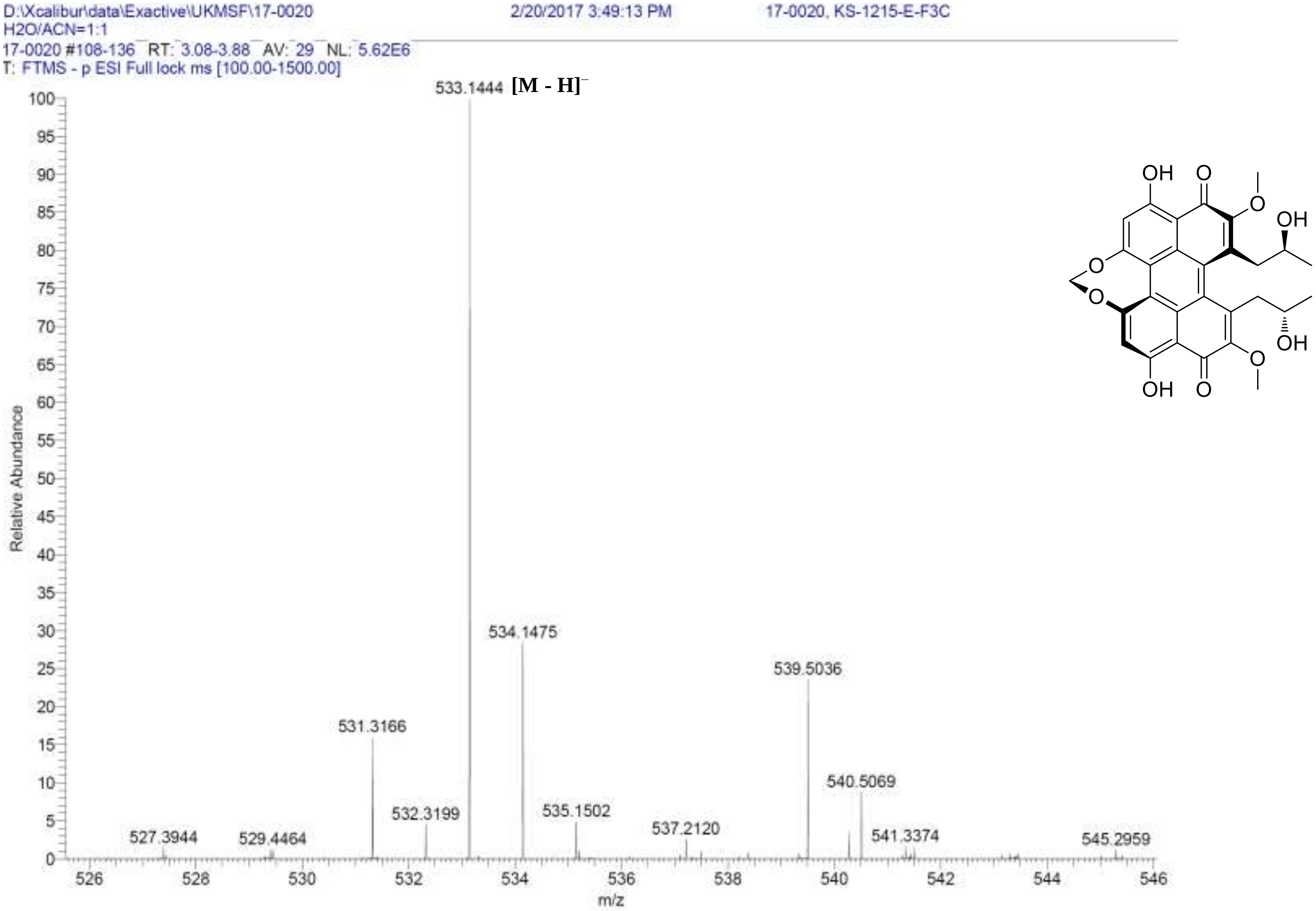

Figure S18. (−)-HRESI-MS spectrum of (+)-isocercosporin (**2**)

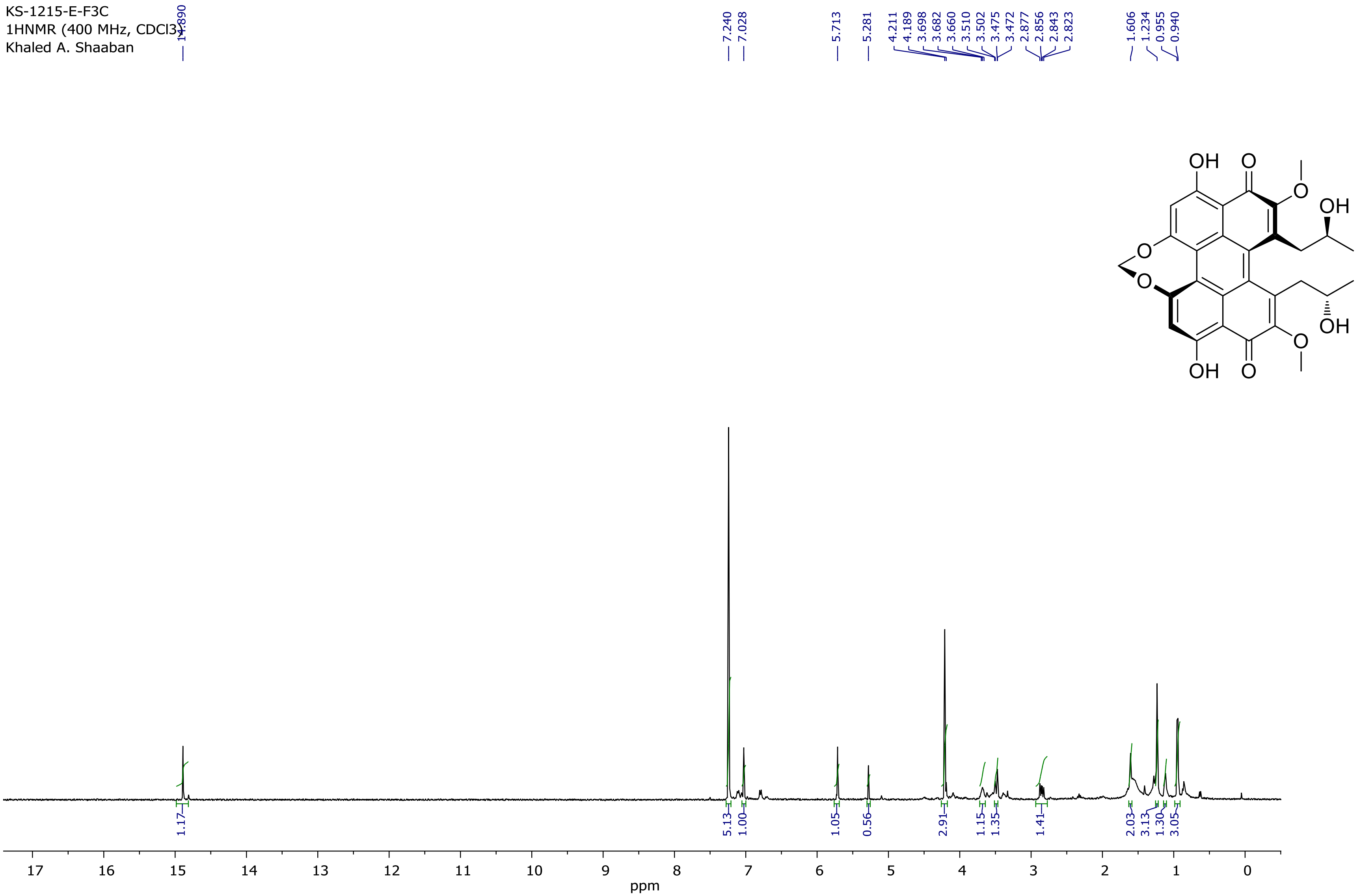

Figure S19. ^{1}H NMR spectrum ($CDCl_3$, 400 MHz) of (+)-isocercosporin (**2**)

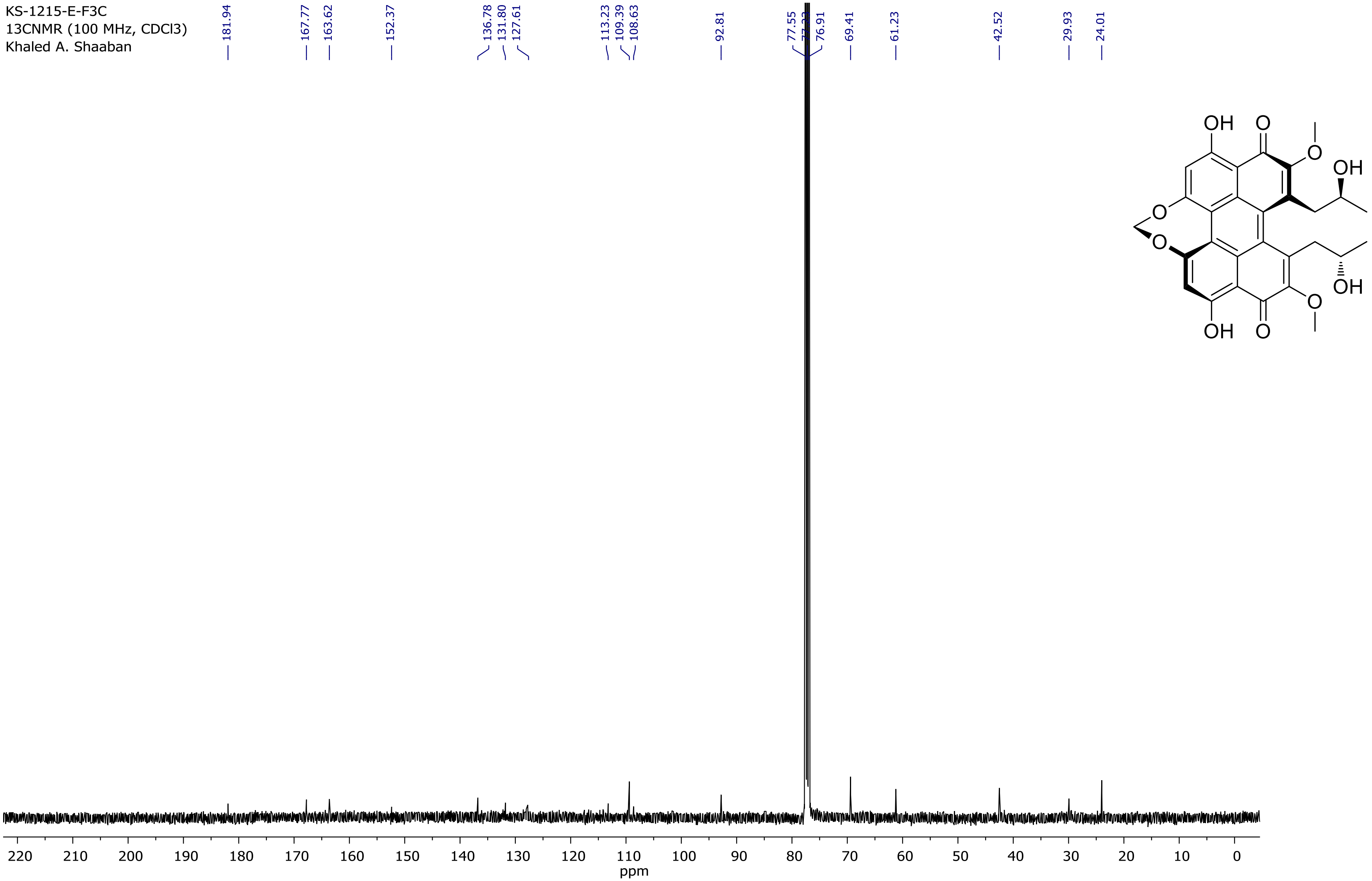

Figure S20. ^{13}C NMR spectrum ($CDCl_3$, 100 MHz) of (+)-isocercosporin (**2**)

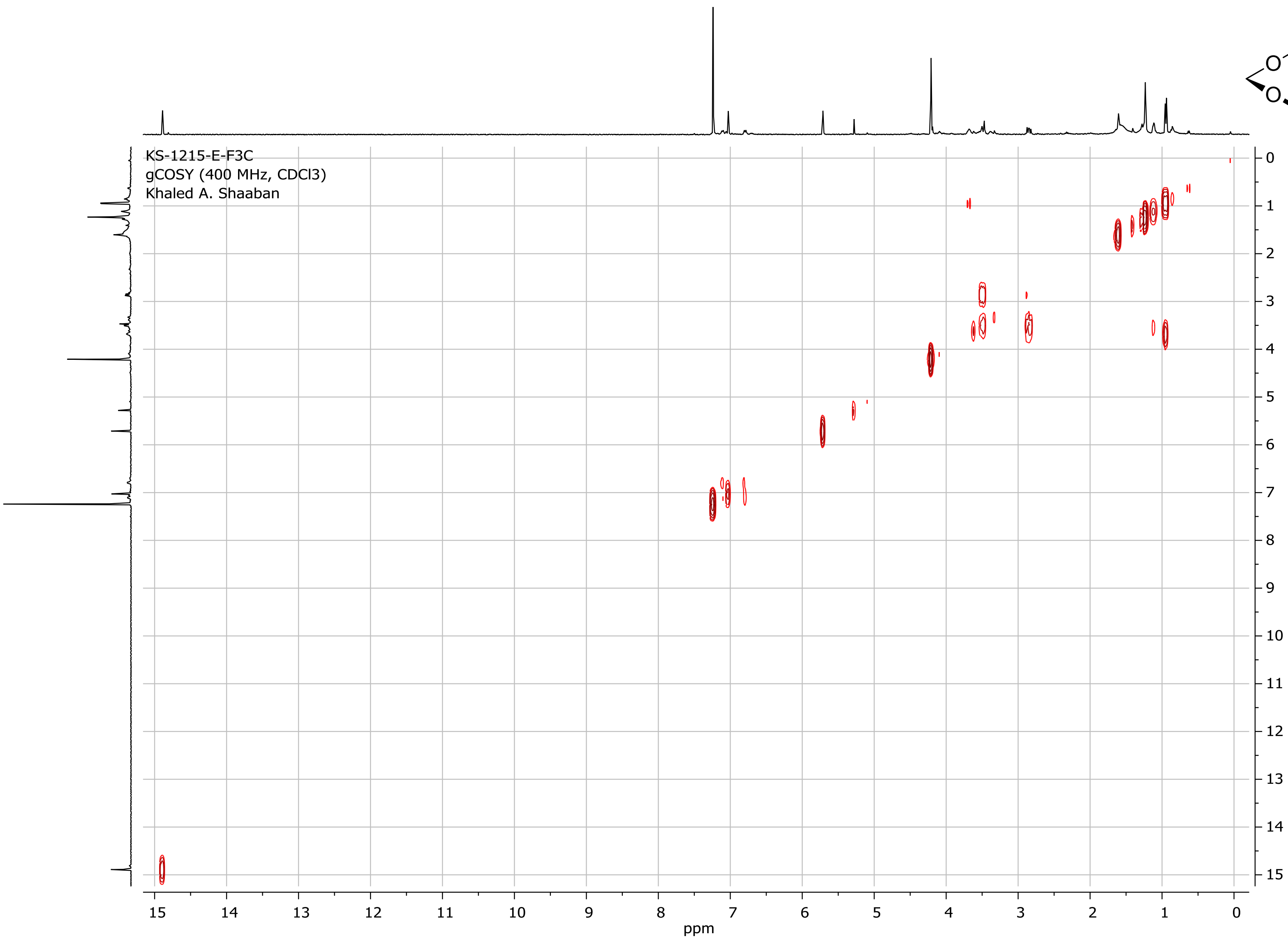

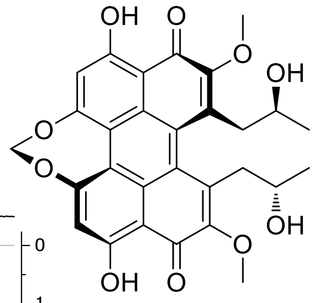

Figure. S21. ^1H-^1H COSY spectrum ($CDCl_3$, 400 MHz) of (+)-isocercosporin (**2**)

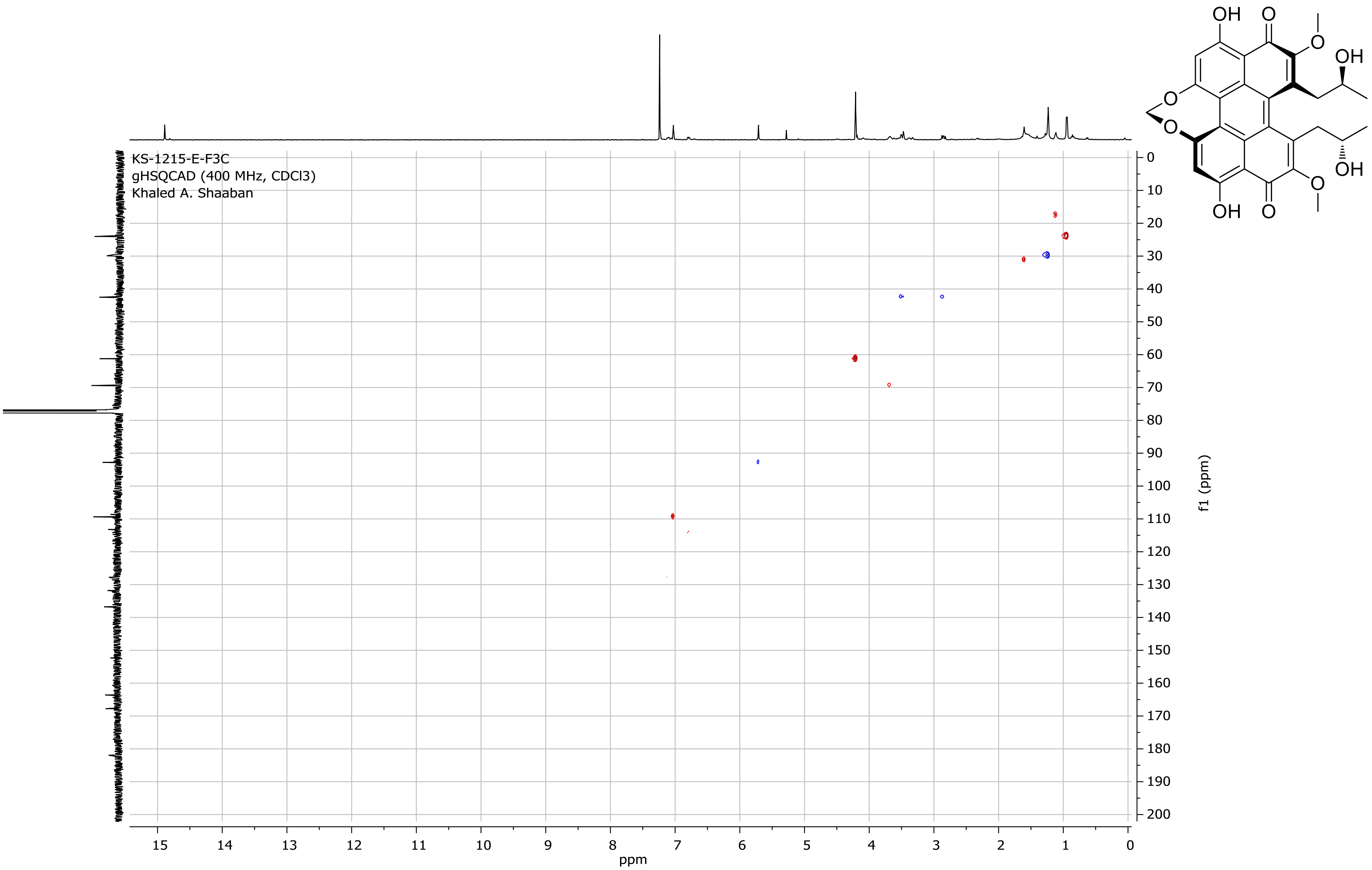

Figure. S22. HSQC spectrum ($CDCl_3$, 400 MHz) of (+)-isocercosporin (**2**)

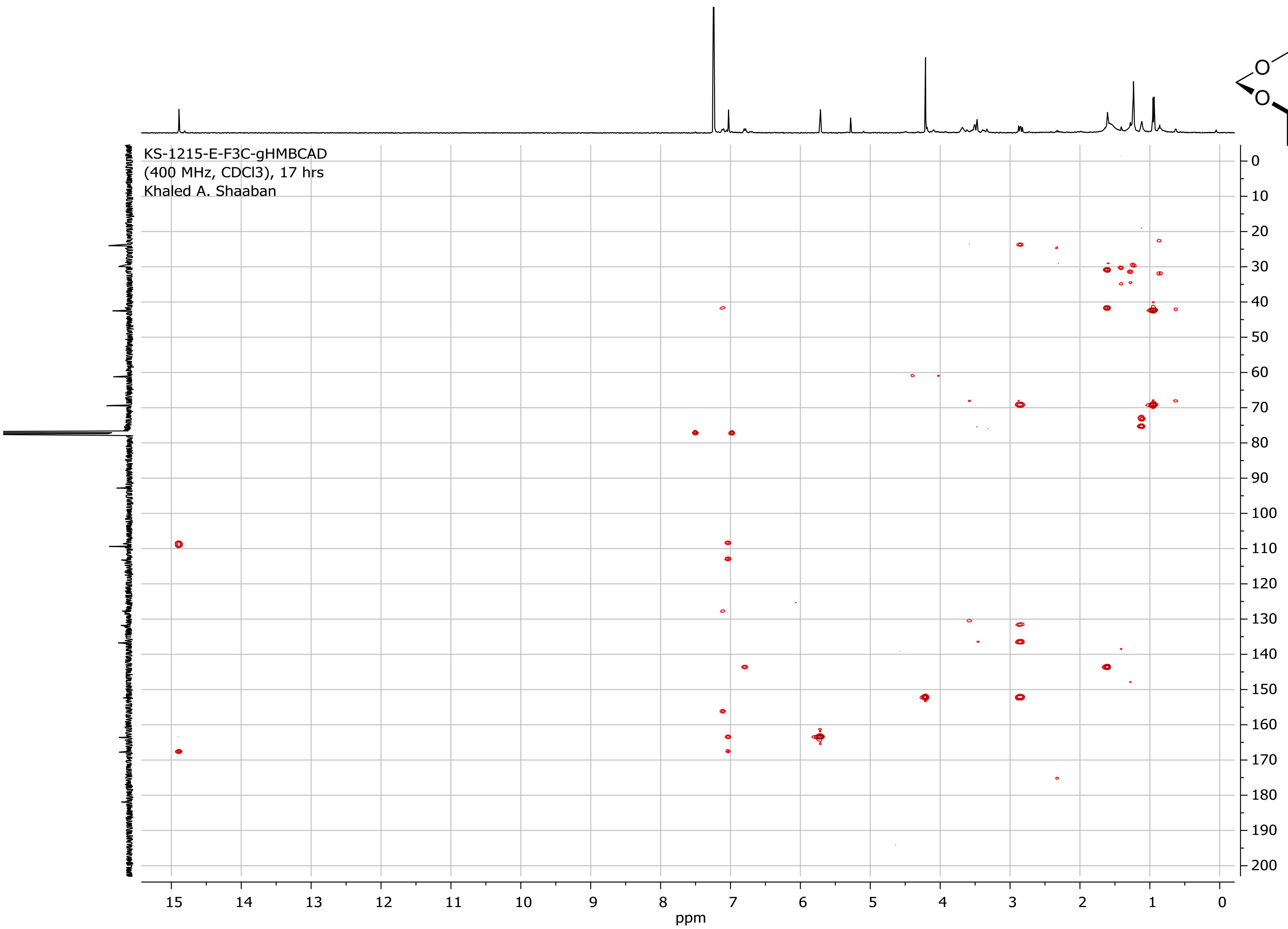

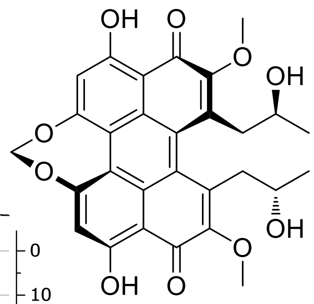

Figure. S23. HMBC spectrum ($CDCl_3$, 400 MHz) of (+)-isocercosporin (**2**)

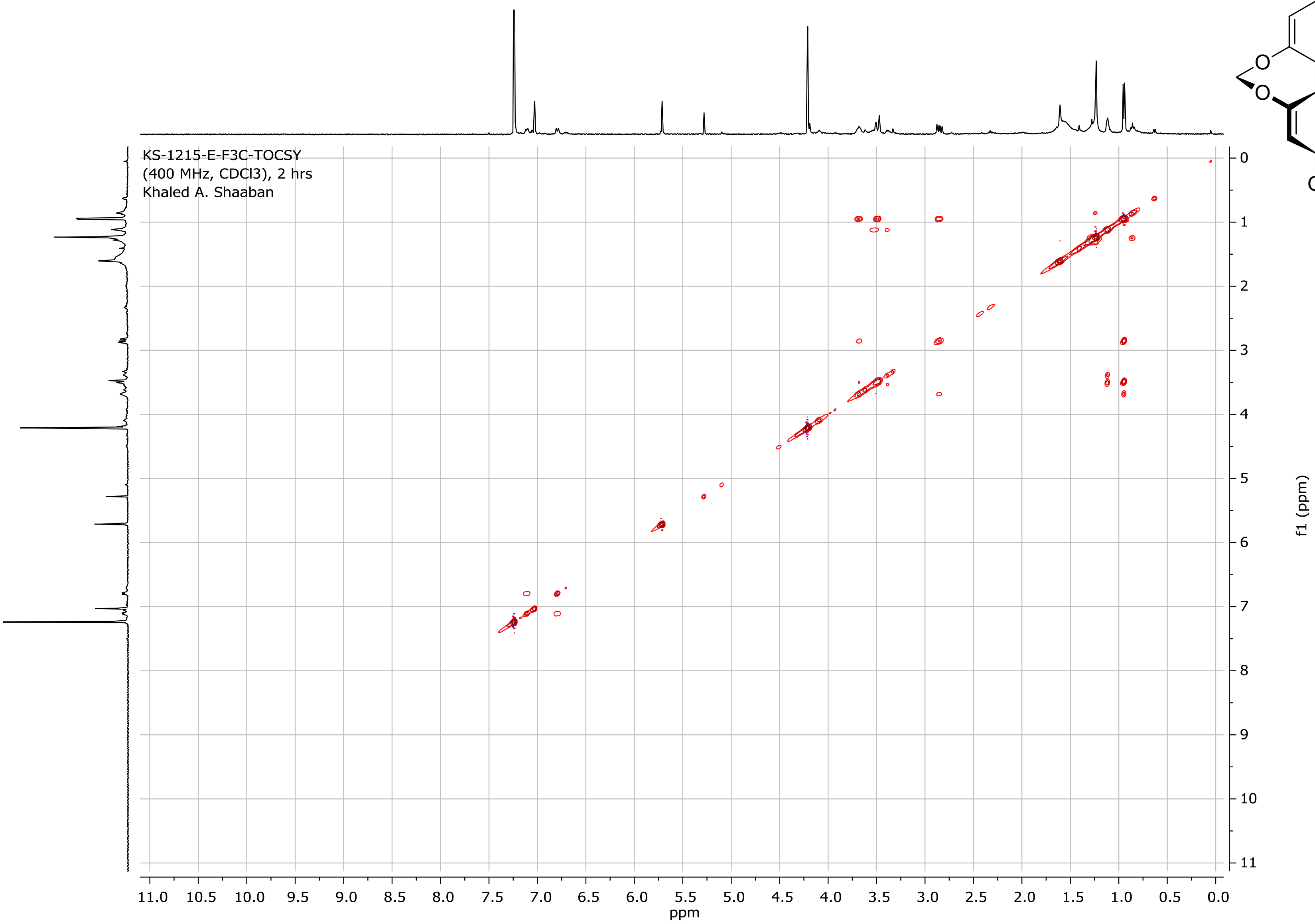

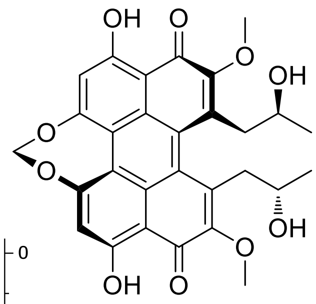

Figure. S24. TOCSY spectrum ($CDCl_3$, 400 MHz) of (+)-isocercosporin (**2**)

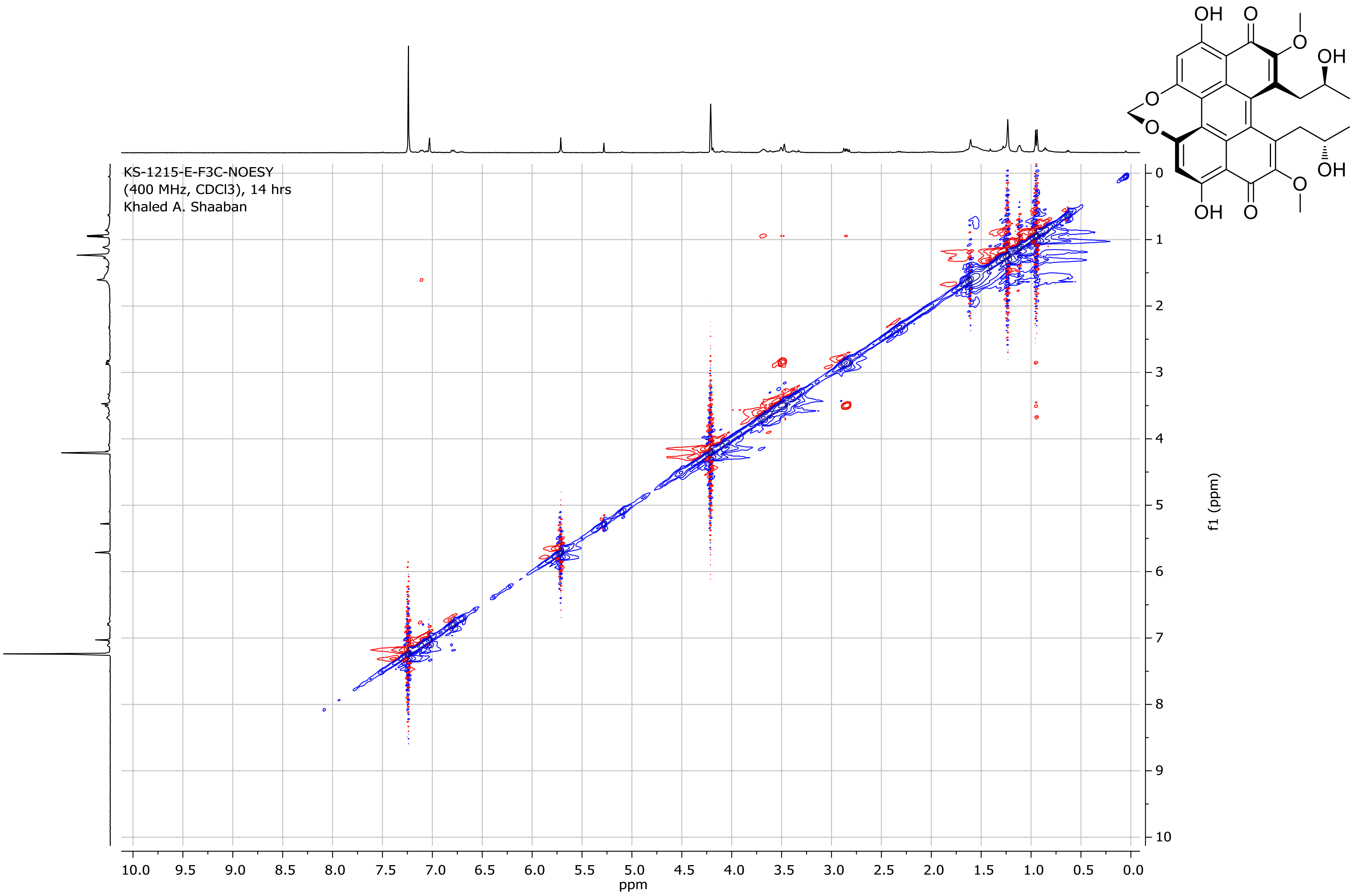

Figure. S25. NOESY spectrum ($CDCl_3$, 400 MHz) of (+)-isocercosporin (**2**)

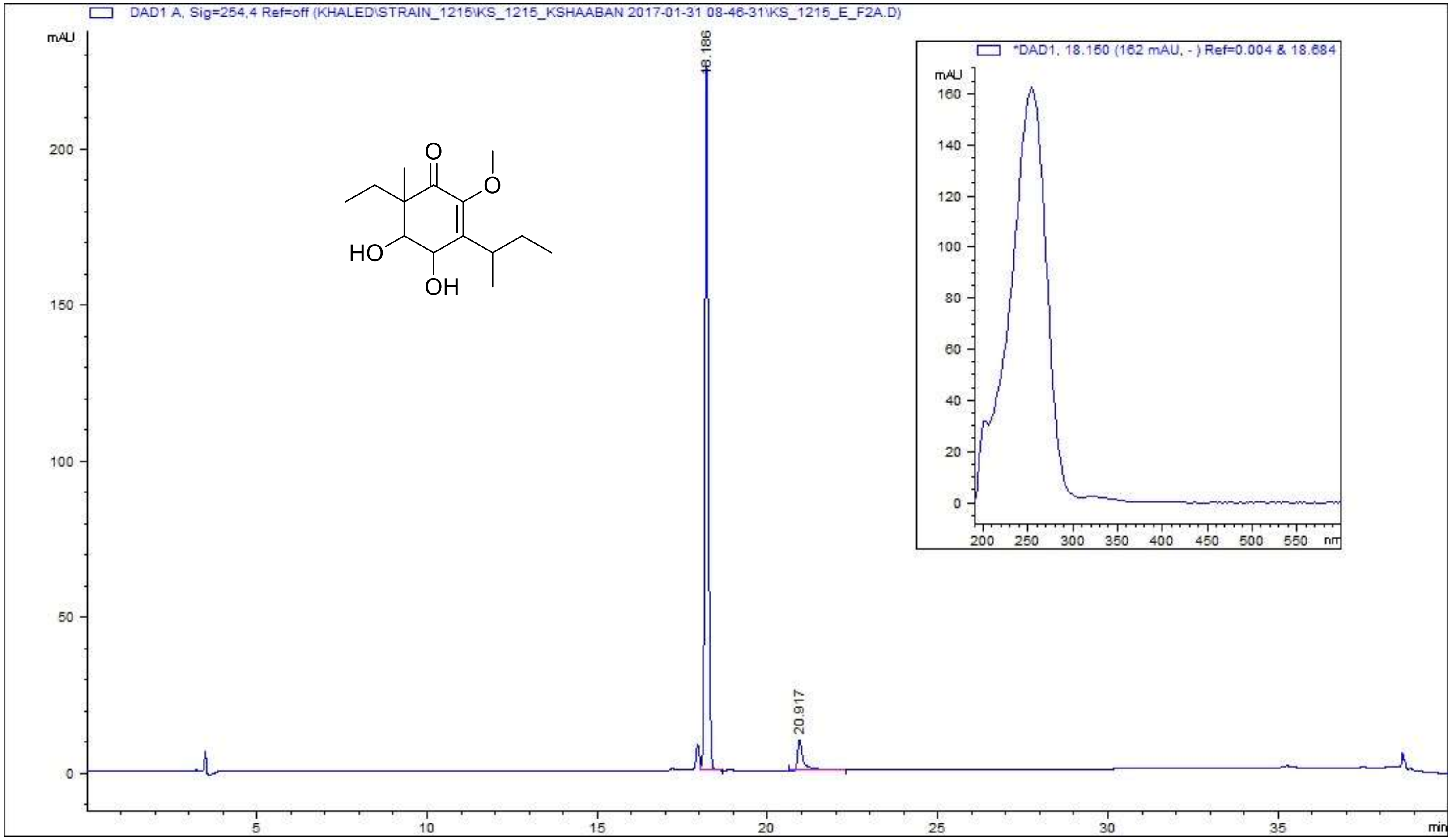

Figure. S26. HPLC/UV analyses of compound **3**. HPLC-conditions: Detection wavelength 254 nm; solvent A: H_2O/0.1% TFA; solvent B: acetonitrile; flow rate: 1.0 mL min^{-1}; 0-35 min, 95-0% A (linear gradient); 35-40 min 0% A; 40-41 min 0-95% A (linear gradient); 41-45 min 95% A.

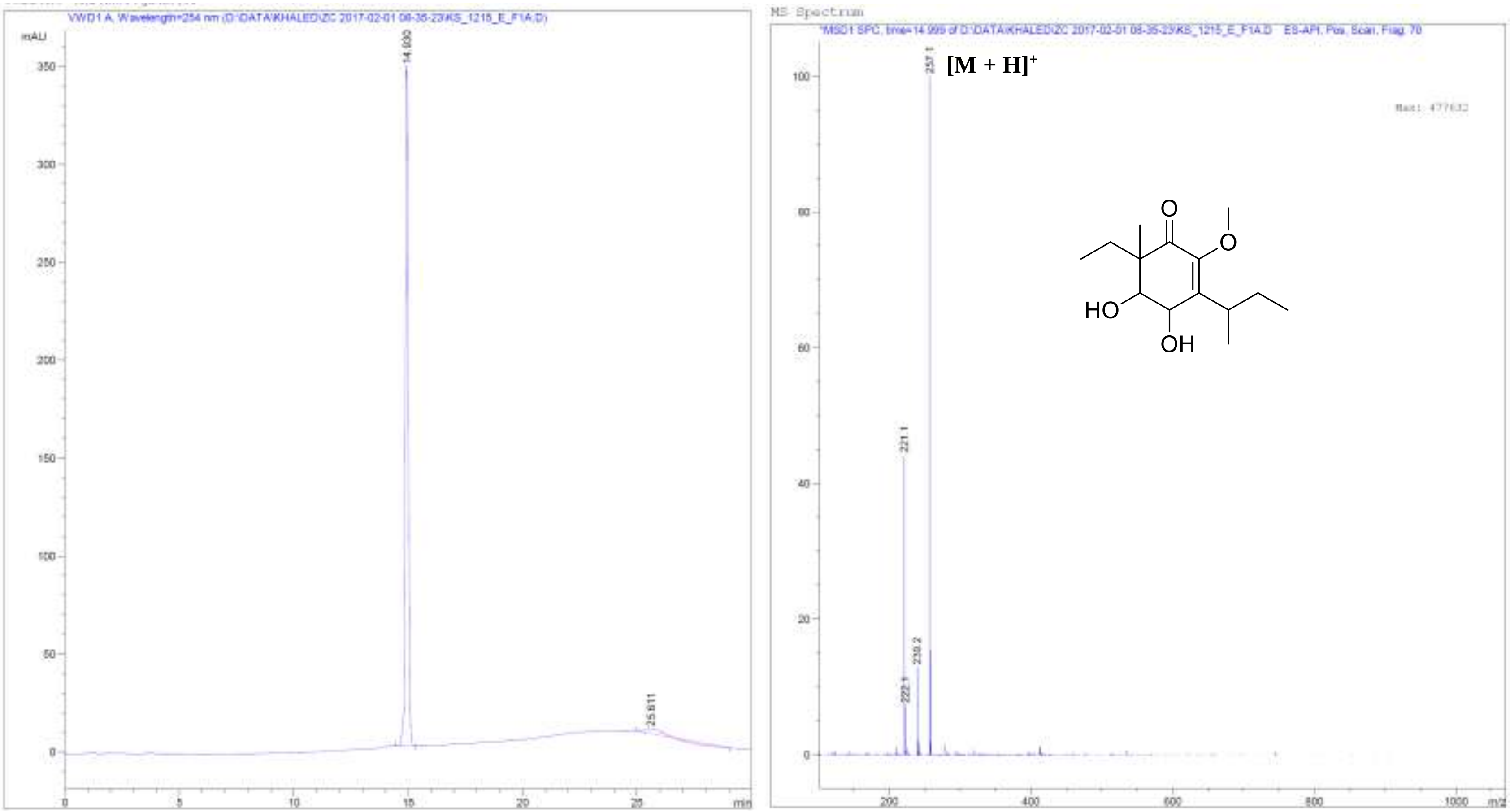

Figure. S27. HPLC/MS analyses of compound **3**. HPLC-conditions: Detection wavelength 254 nm; solvent A: H_2O/0.1% Formic acid, solvent B: CH_3CN/0.1% Formic acid; flow rate: 0.5 mL min^{-1}; 0-4 min, 10% B; 4-22 min, 10-100% B; 22-27 min, 100% B; 27-29 min, 100%-10% B; 29-30 min, 10 % B.

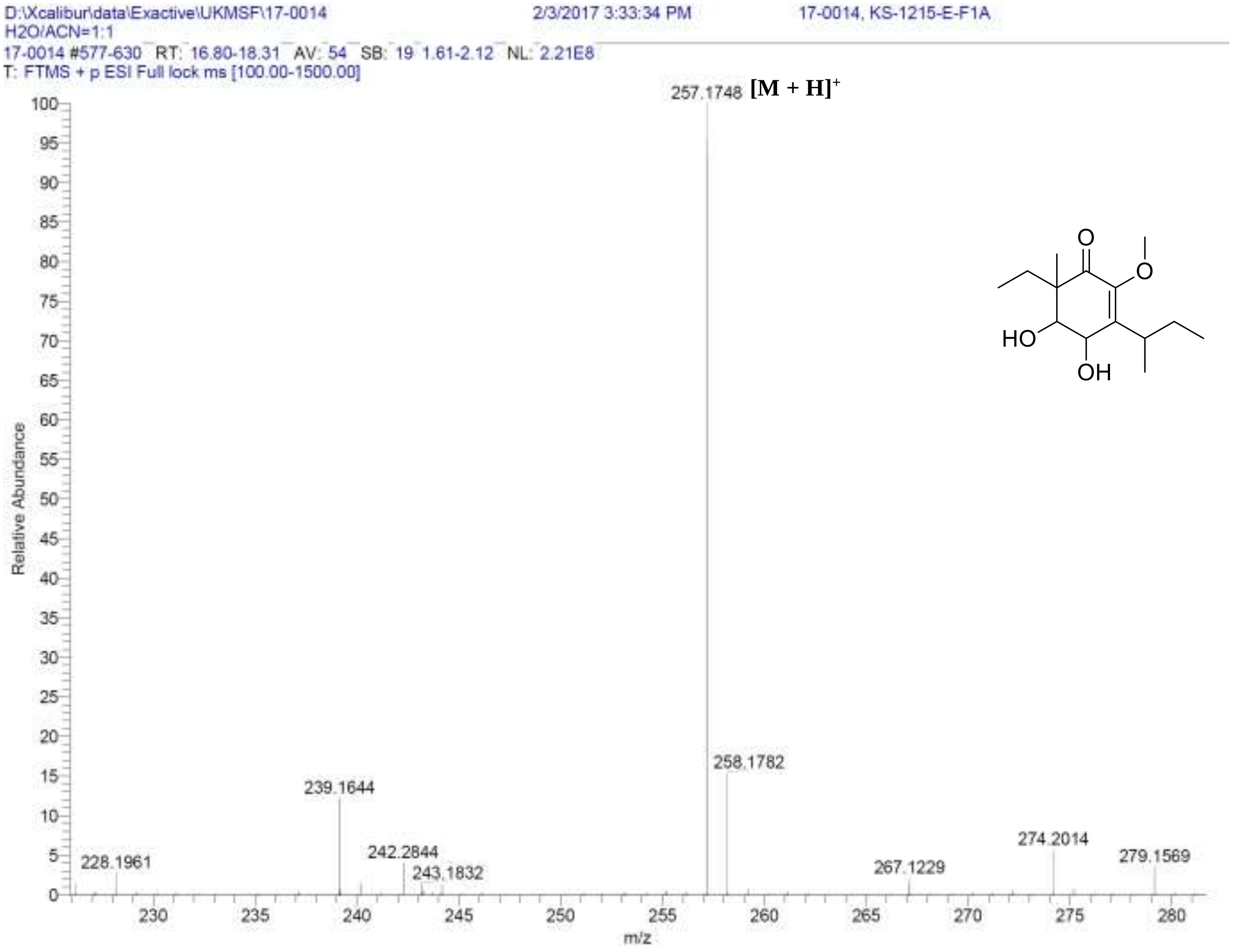

Figure. S28. (+)-HRESI-MS spectrum of compound **3**

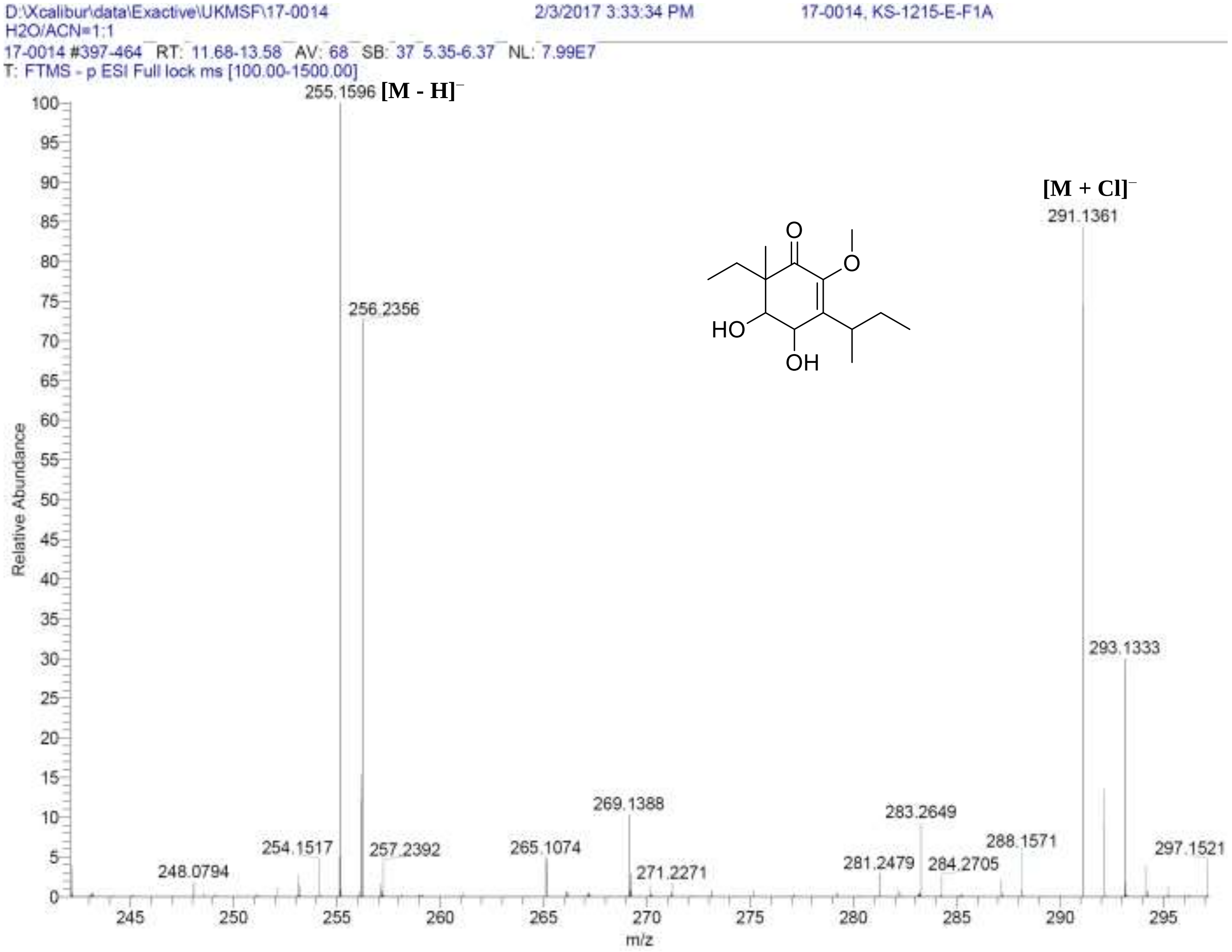

Figure. S29. (−)-HRESI-MS spectrum of compound **3**

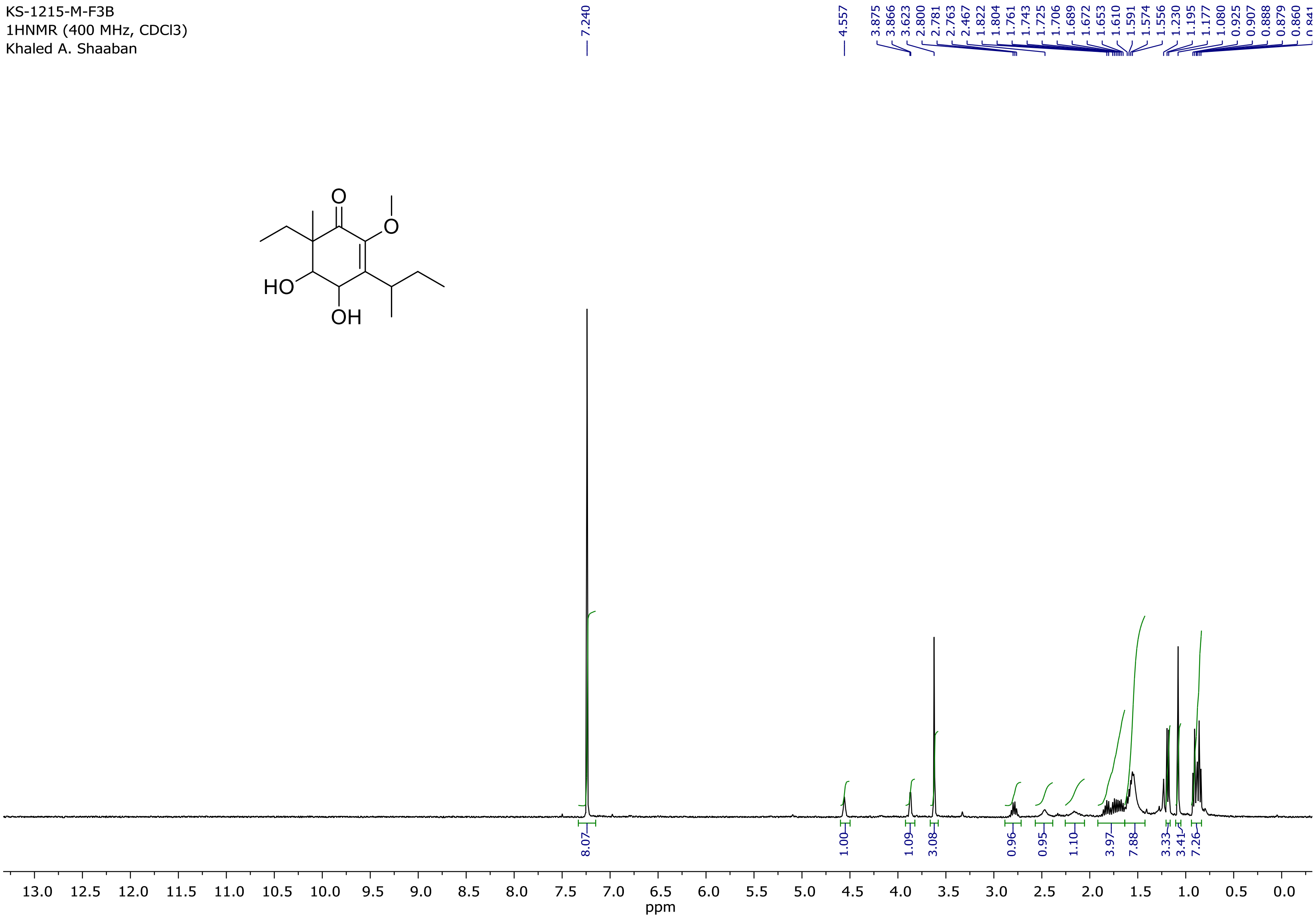

Figure. S30. ^{1}H NMR spectrum ($CDCl_3$, 400 MHz) of compound **3**

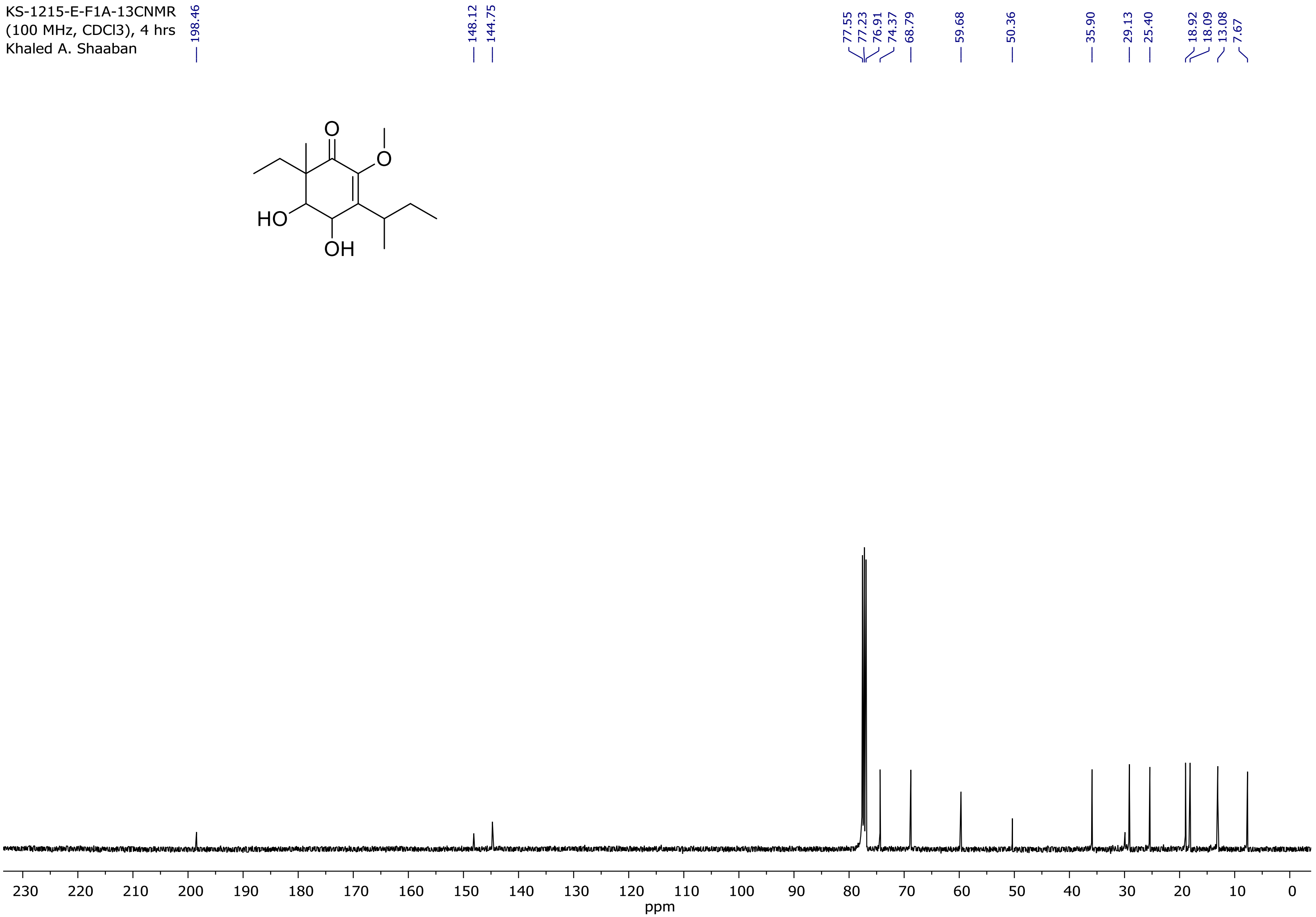

Figure. S31. ^{13}C NMR spectrum ($CDCl_3$, 100 MHz) of compound **3**

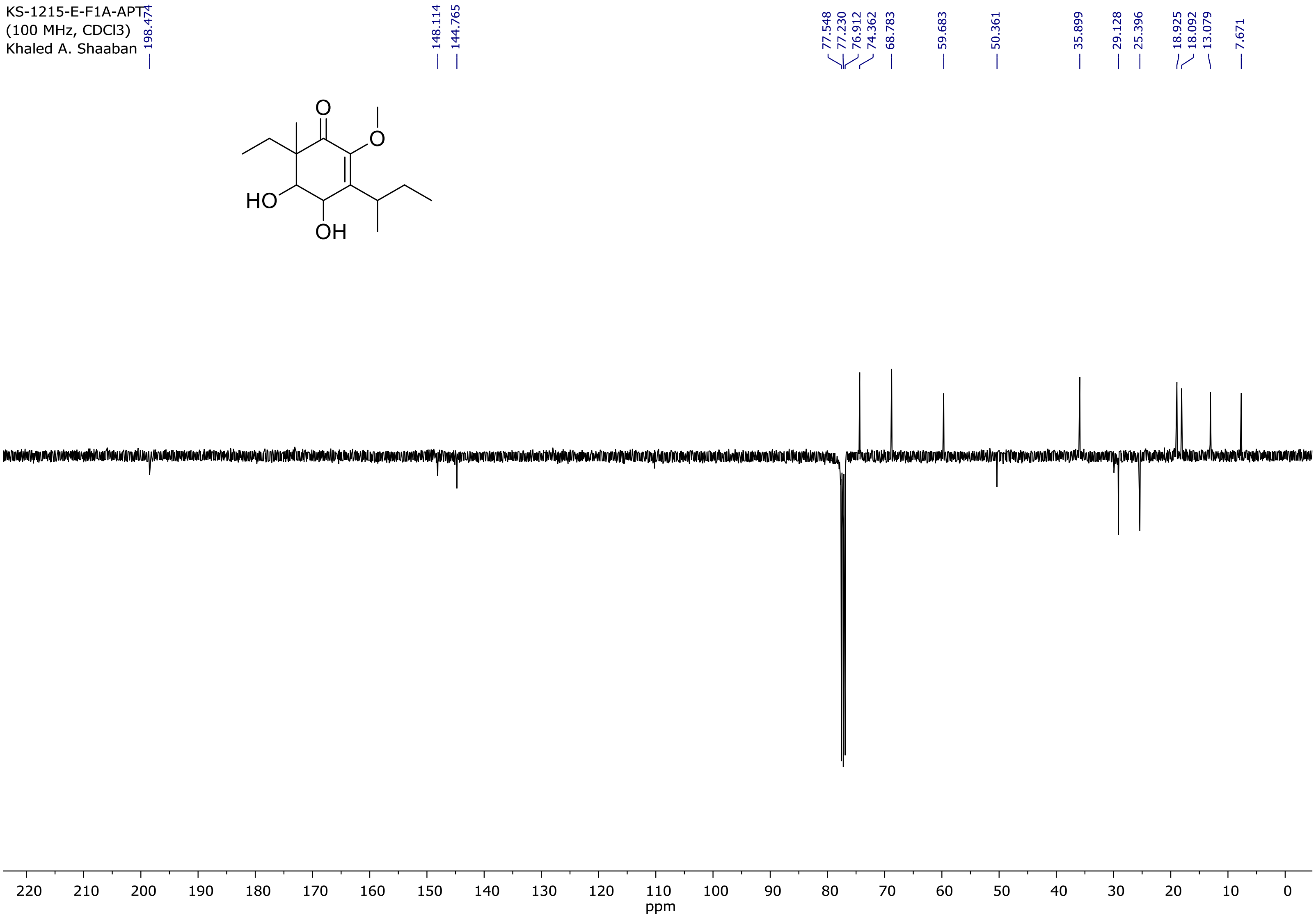

Figure. S32. APT NMR spectrum ($CDCl_3$, 100 MHz) of compound **3**

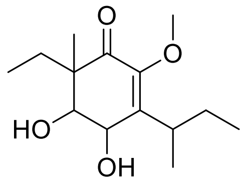

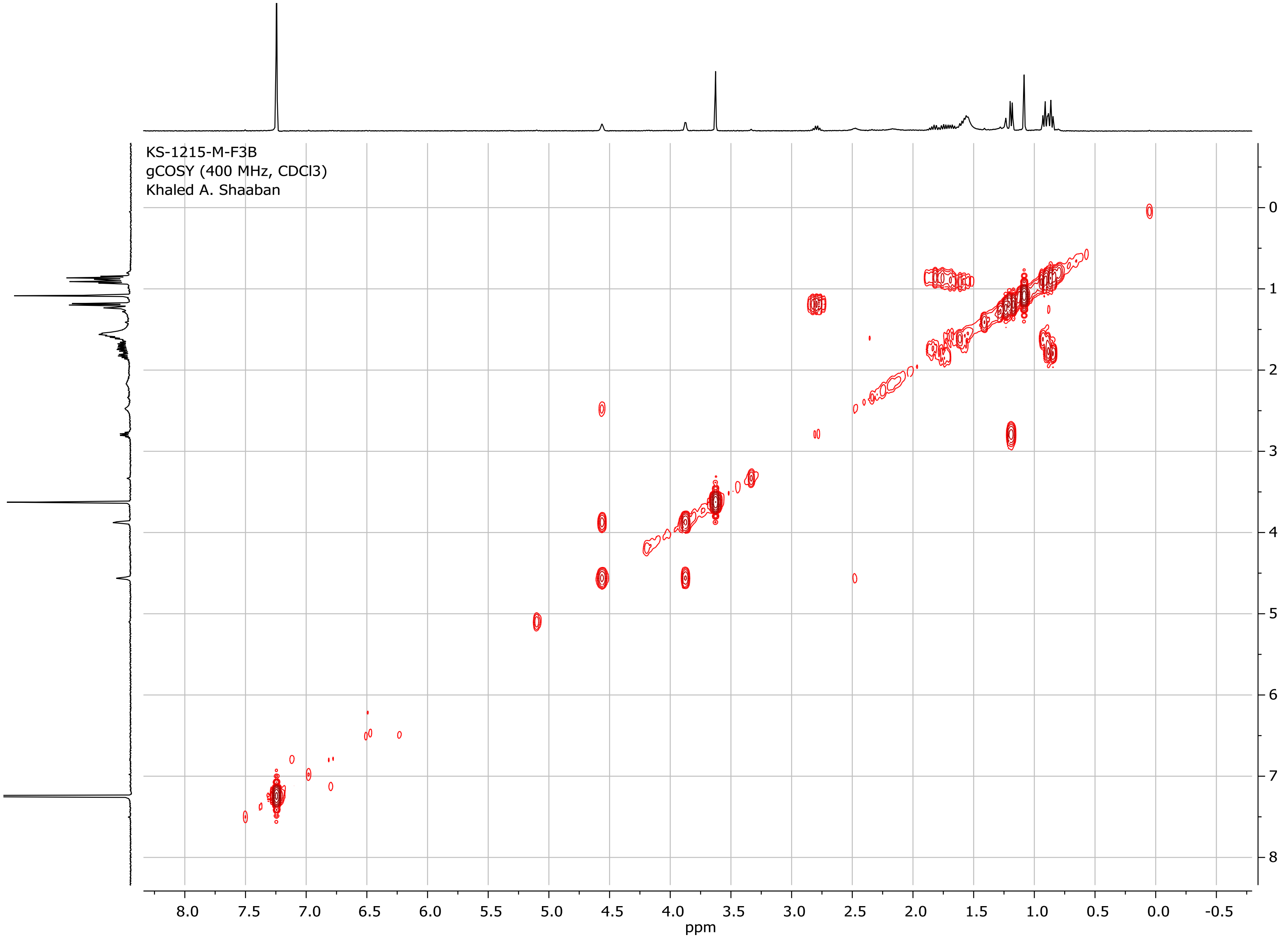

Figure. S33. ^1H-^1H COSY spectrum ($CDCl_3$, 400 MHz) of compound **3**

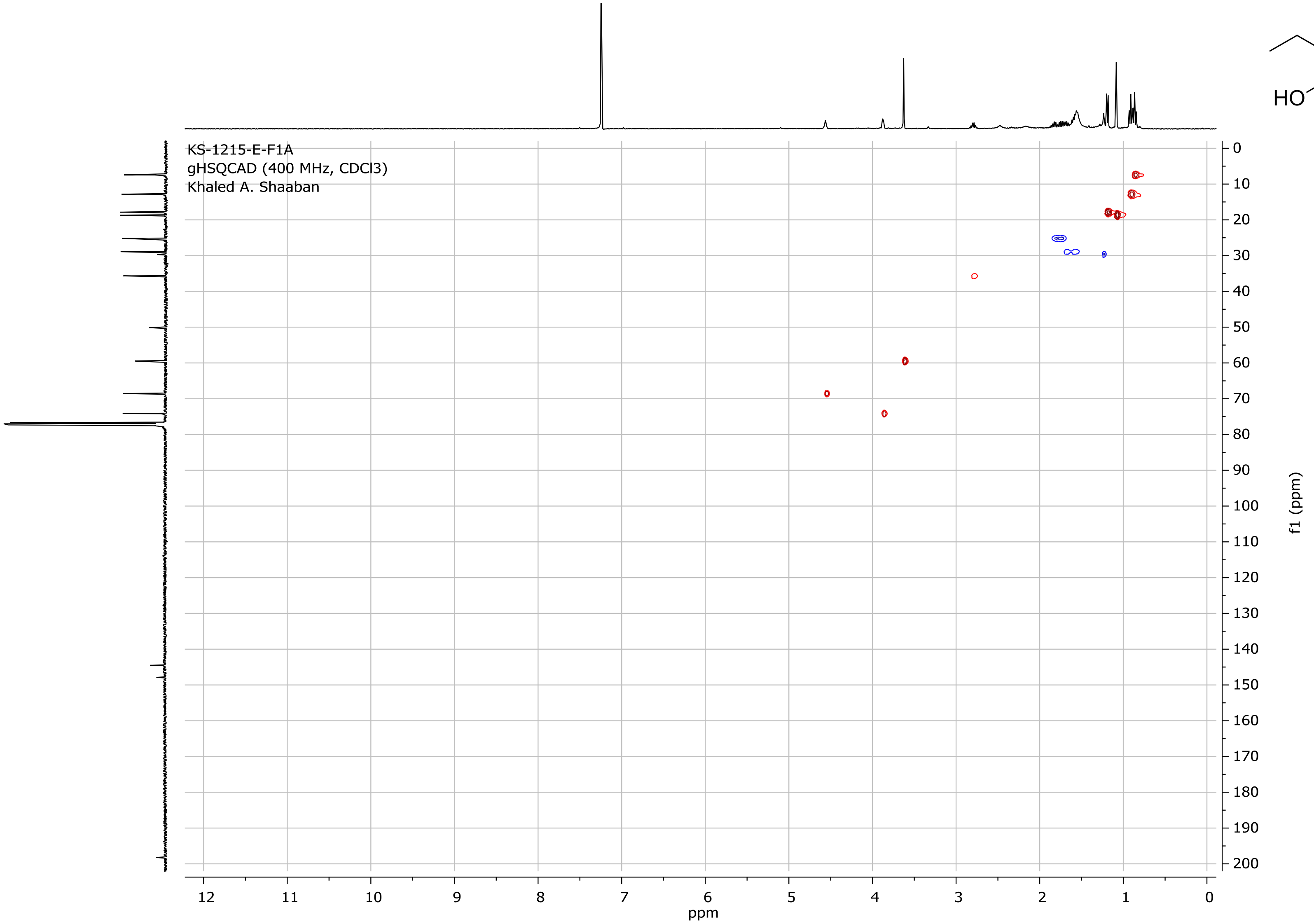

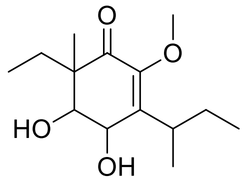

Figure. S34. HSQC spectrum ($CDCl_3$, 400 MHz) of compound **3**

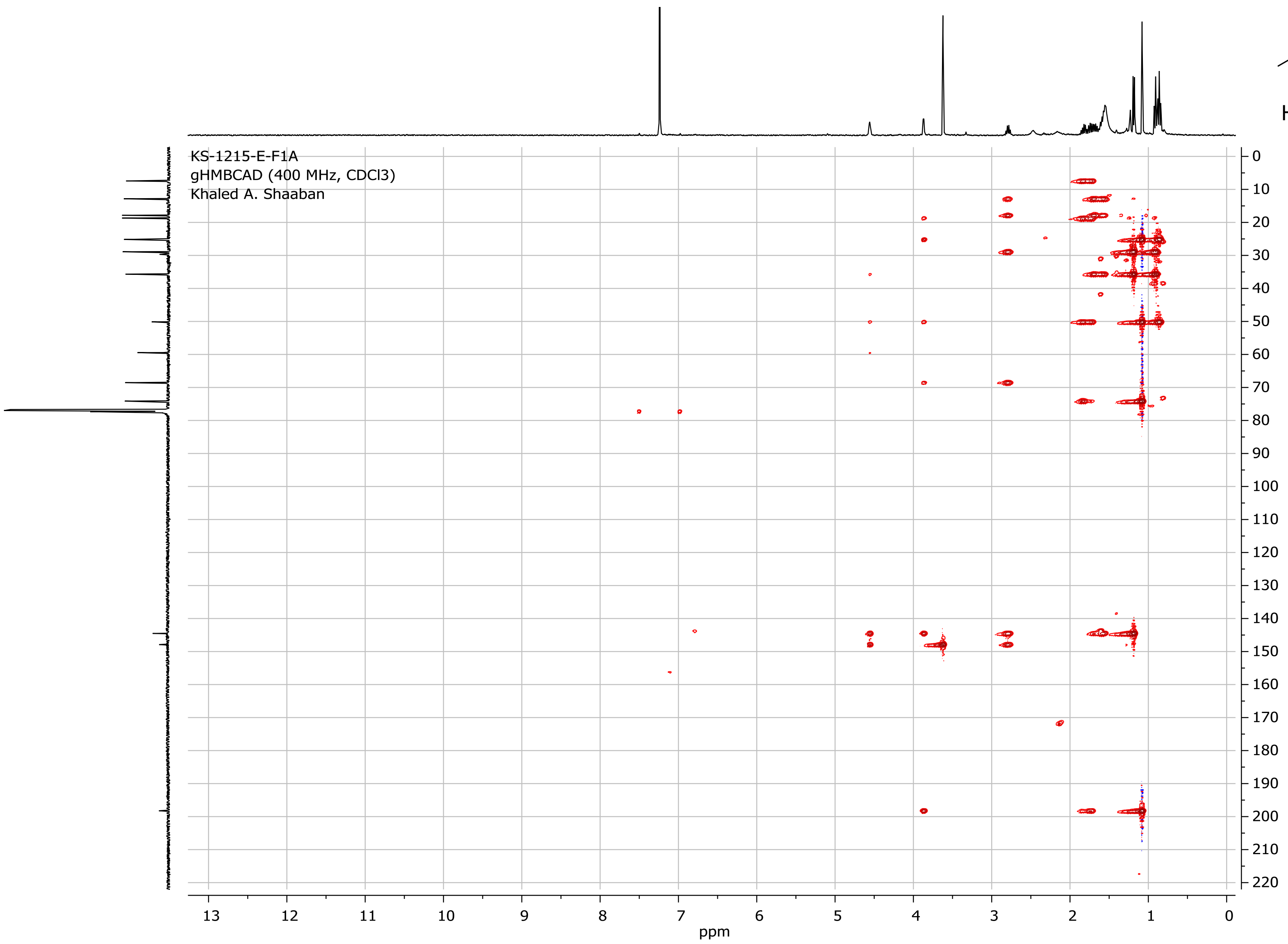

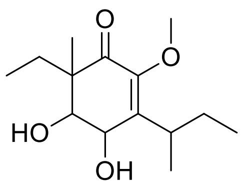

Figure. S35. HMBC spectrum ($CDCl_3$, 400 MHz) of compound **3**

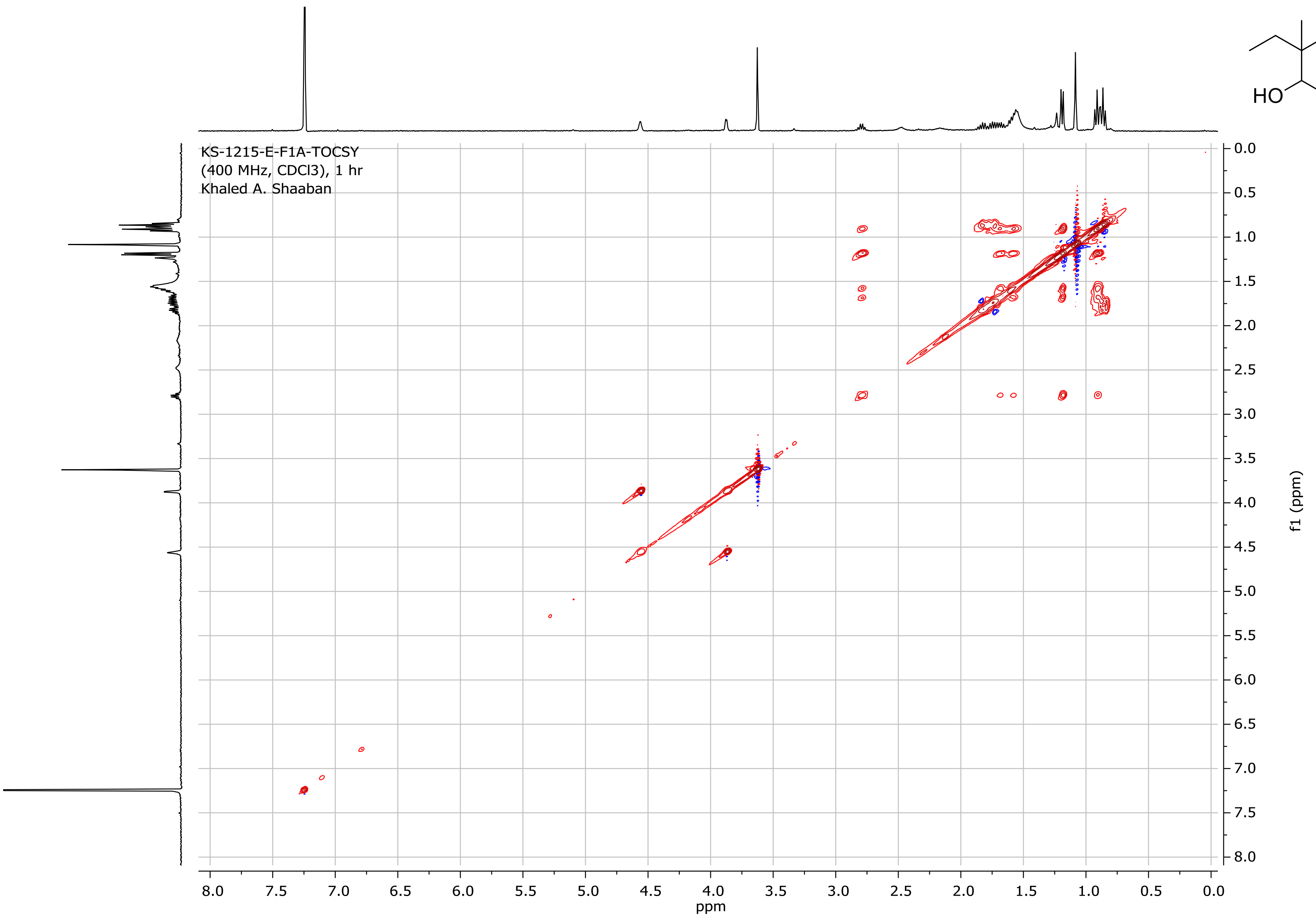

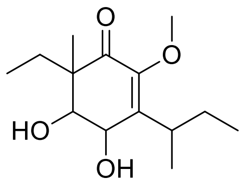

Figure. S36. TOCSY spectrum ($CDCl_3$, 400 MHz) of compound **3**

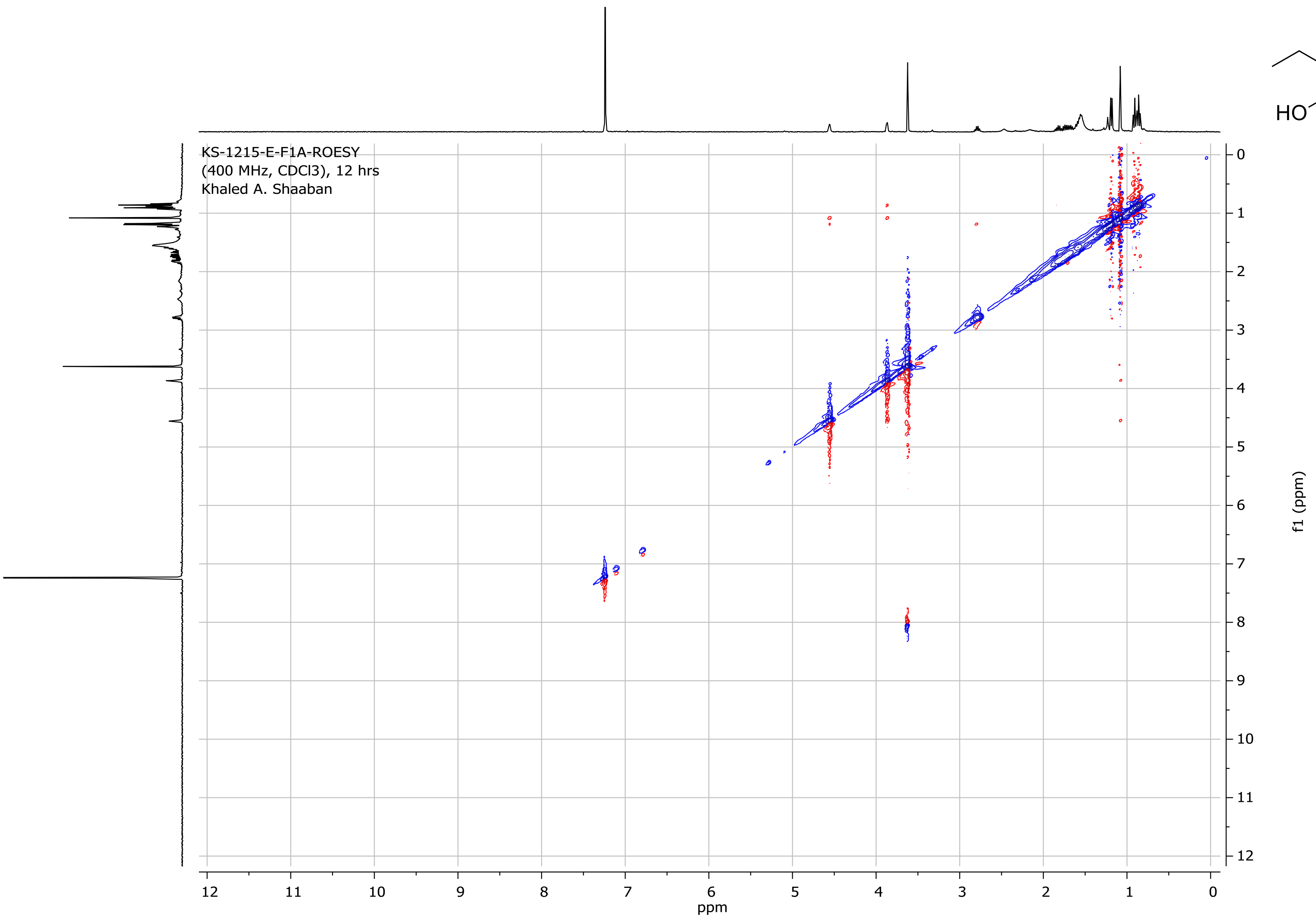

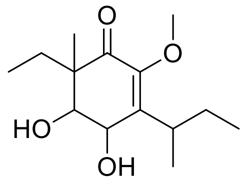

Figure. S37. ROESY spectrum ($CDCl_3$, 400 MHz) of compound **3**

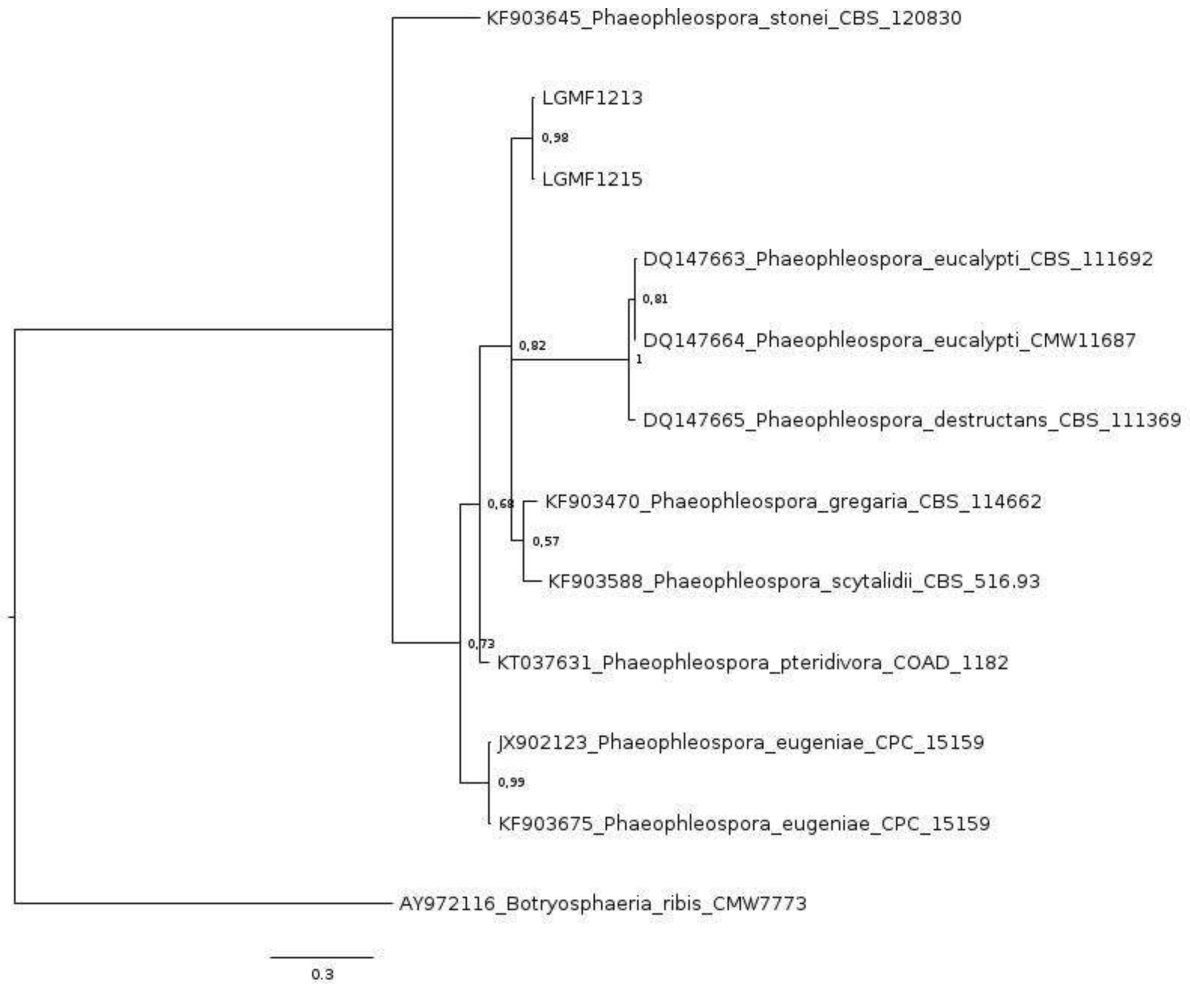

Figure S38. Bayesian phylogenetic tree based on actin gene partial sequence of LGMF1213 and LGMF1215 and sequence of species of *Phaeophleospora* genus available in the Genbank. Values on the node indicate Bayesian posterior probabilities. The species *Botryosphaeria ribis* was used as outgroup. Scale bar indicates the number of substitutions per site.

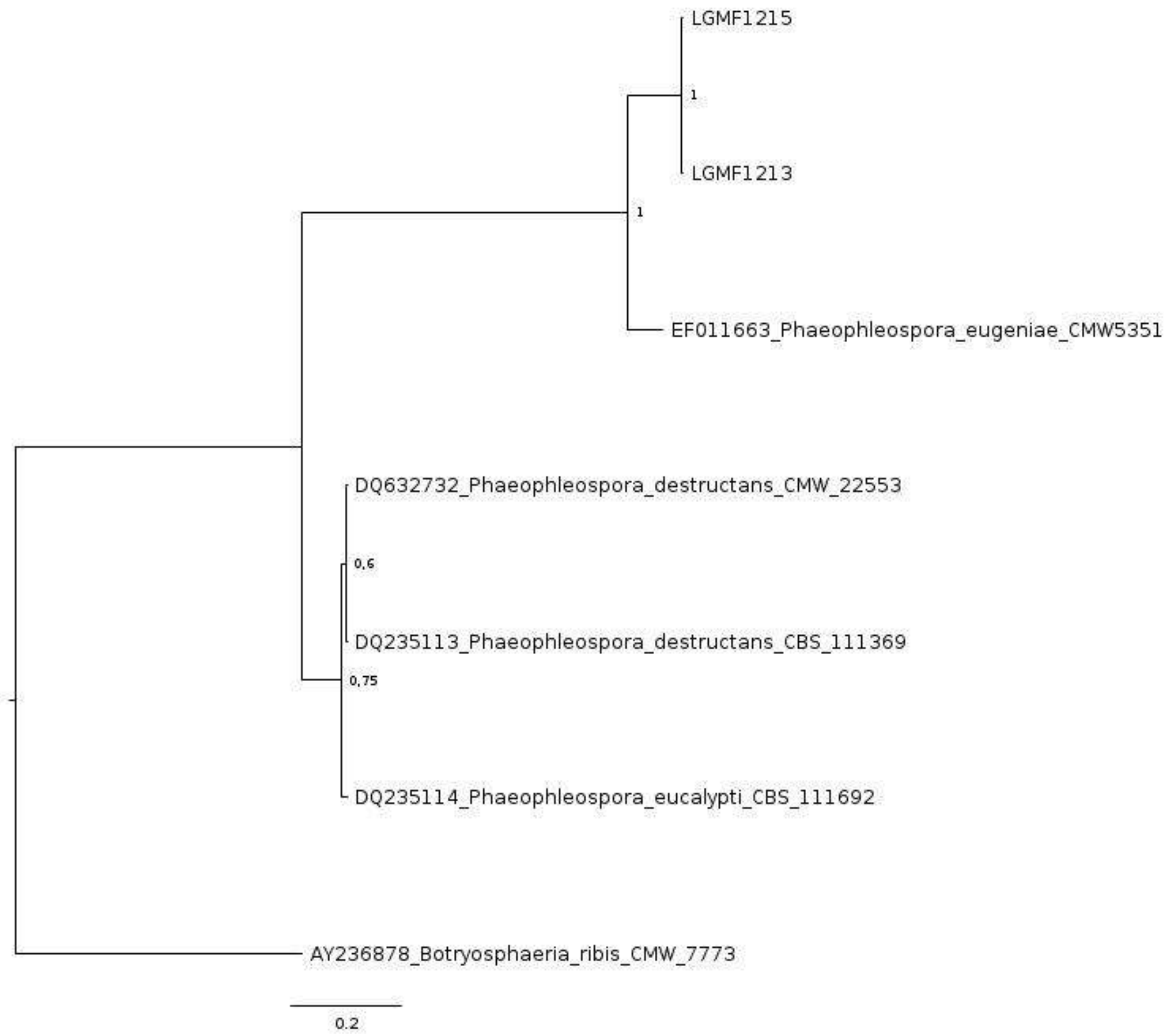

Figure S39. Bayesian phylogenetic tree based on elongation factor gene sequence of LGMF1213 and LGMF1215 and sequence of species of *Phaeophleospora* genus available in the Genbank. Values on the node indicate Bayesian posterior probabilities. The species *Botryosphaeria ribis* was used as outgroup. Scale bar indicates the number of substitutions per site.

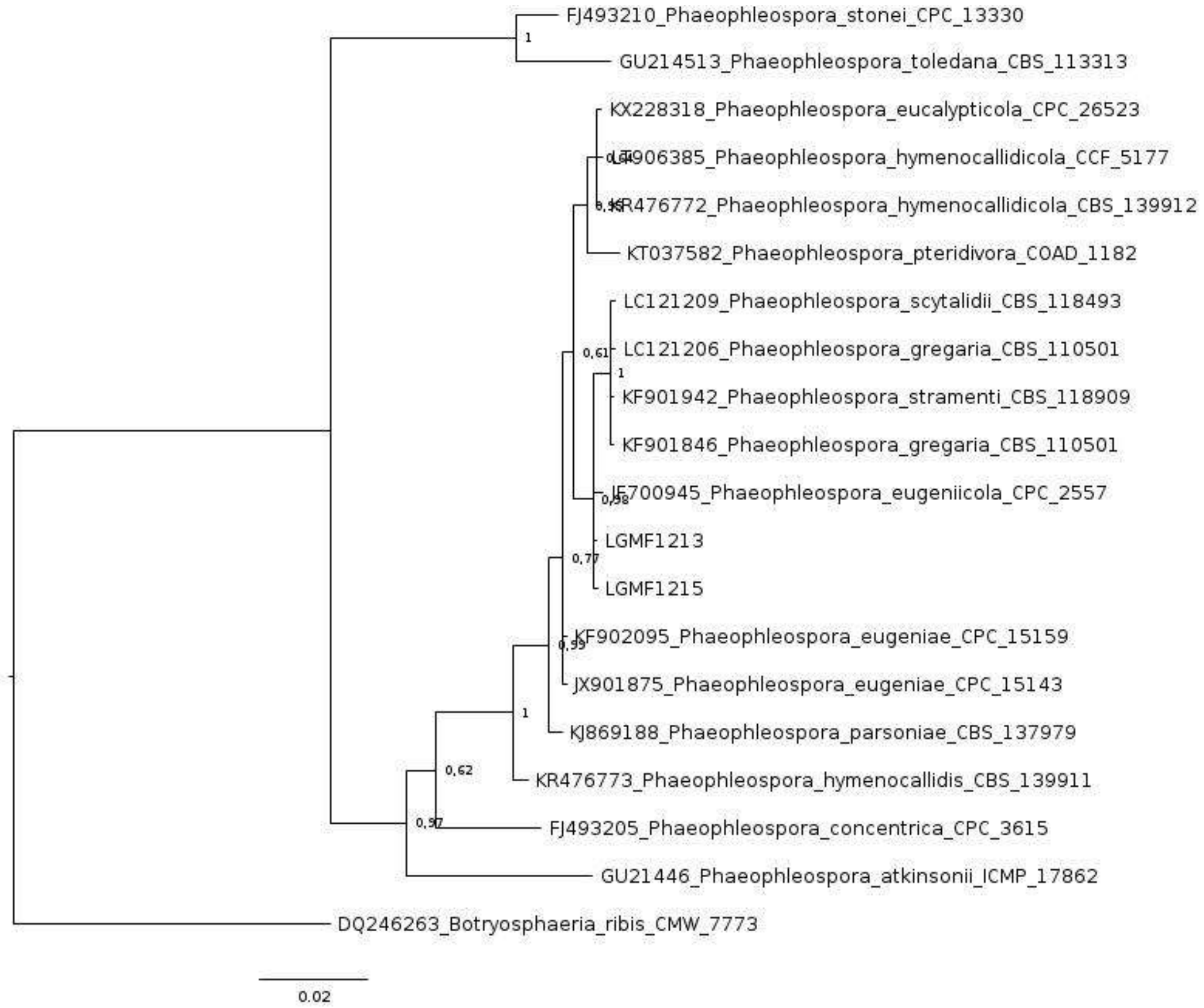

Figure S40. Bayesian phylogenetic tree based on LSU sequence of LGMF1213 and LGMF1215 and sequence of species of *Phaeophleospora* genus available in the Genbank. Values on the node indicate Bayesian posterior probabilities. The species *Botryosphaeria ribis* was used as outgroup. Scale bar indicates the number of substitutions per site.

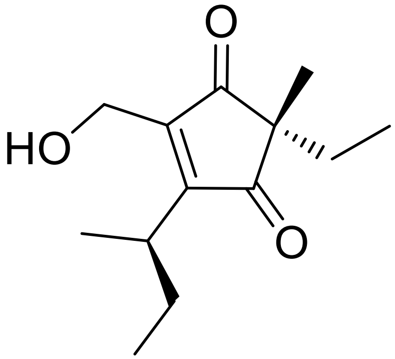

Similin A (**4**)

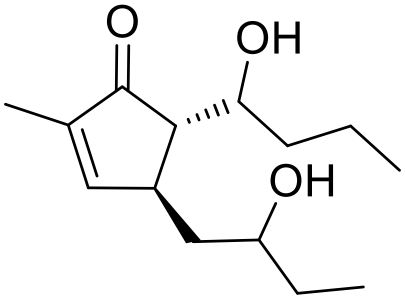

Phomapentenone A (**5**)

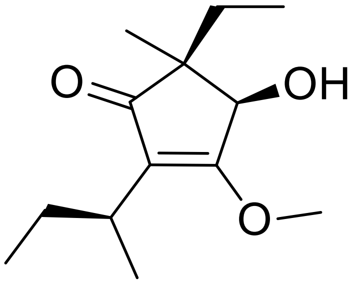

Boydone B (**6**)

Figure S41. Chemical structures of compounds **4-6**

Table S1. Collection details and GenBank accession number of isolates included in this study.

Species	Culture accession numbers	Host/Isolation source	Host family	Tissue source	Country	ITS GenBank accession numbers
Phaeophleospora eugeniae	CPC15143	*Eugenia uniflora*	*Myrtaceae*	Leaf spot	Brazil	K901615
	CPC15159	*Eugenia uniflora*	*Myrtaceae*	Leaf spot	Brazil	K901742
P. gregaria	CBS110501	*Eycalyptus globulus*	*Myrtaceae*	Leaf spot	Australia	KF901524
P. hymenocallidicola	CBS 139912 = CPC 25014	unkown fern	*Polypodiaceae*	Leaf spot	Thailand	KR476739
		unkown fern	*Polypodiaceae*	Leaf spot	Thailand	NR137994
P. hymenocallidis	CBS 139911 = CPC 25018	unkown fern	*Polypodiaceae*	Leaf spot	Thailand	KR476740
		unkown fern	*Polypodiaceae*	Leaf spot	Thailand	NR137995
P. pteridivora	CPC 24683 = COAD 1182	*Serpocaulon triseriale*	*Polypodiaceae*	Leaf spot	Brazil	KT037547
P. scytalidii	CBS 118493 = CPC 10998	*Eucalyptus urophylla*	*Myrtaceae*	Leaf spot	Colombia	KF901631
	CBS 516.93 = CPC 653	*Eucalyptus globulus*	*Myrtaceae*	Leaf spot	Brazil	LC121138
P. stonei	CBS 120830 = CPC 13330	*Eucalyptus* sp.	*Myrtaceae*	Leaf spot	Australia	KF901525
P. stramenti	CBS 118909 = CPC 11545	*Eucalyptus* sp.	*Myrtaceae*	leaf	Brazil	KF901617
P. atkinsonii	CBS 124565	*Hebe* sp.	*Plantaginaceae*	Leaf spot	New Zealand	GU214643
	CBS 124566	*Hebe* sp.	*Plantaginaceae*	Leaf spot	New Zealand	GU214644
P. capensis	CPC 13981	*Protea repens*	*Proteaceae*	Leaf spot	Portugal	EU707887
P. concentrica	CPC 3615	*Protea caffra*	*Proteaceae*	Leaf spot	Kenya	FJ493187
P. elaeocarpi	CPC 13309	*Elaeocarpus* sp.	*Elaeocarpaceae*	Leaf spot	Australia	EU040212
P. eucalypticola	CPC 26523	*Eucalyptus robusta*	*Myrtaceae*	leaf	France	KX228267
		Eucalyptus robusta	*Myrtaceae*	leaf	France	NR145123
P. eugeniicola	CPC 2558	*Eugenia uniflora*	*Myrtaceae*	Leaf spot	Brazil	FJ493191
	CPC 2557	*Eugenia uniflora*	*Myrtaceae*	Leaf spot	Brazil	FJ493190
P. parsoniae	CBS 137979	*Parsonia straminea*	*Apocynaceae*	Leaf spot	Australia	KJ869131
P. abyssinicae	BPI 38386	*Protea gaguedi*	*Proteaceae*	Leaf spot	Ethiopia	No sequence available
P. congestum	BPI 368056	*Protea caffra*	*Proteaceae*	Leaf spot	South Africa	No sequence available
P. faureae	---	*Faurea saligna*	*Proteaceae*	Leaf spot	South Africa	No sequence available
P. indica	CBS97153	*Achras sapota*	*Sapotaceae*	Leaf spot	Bangalore	No sequence available
P. vochysiae	LGMF1215	*Vochysia divergens*	*Vochysiaseae*	leaf	Brazil	KY754582
	LGMF1213	*Vochysia divergens*	*Vochysiaseae*	leaf	Brazil	KY754583

Table S2. Morphological characters of isolate *P. vochysiae* LGMF1215 and related *Phaeophleospora* species

Structure	*P. eugeniicola*		*P. gregaria*		*P. scytalidii*		*P. vochysiae*	
	**Description**	**Measurement**	**Description**	**Measurement**	**Description**	**Measurement**	**Description**	**Measurement**
**Conidiophores**	Mostly reduced to conidiogenous cells, or multi-septate, subcylindrical, branched or not, brown	15–25 × 4–5 µm	lack the asexual state		aggregated in loose fascicles arising from the upper cells of a brown stroma up to 50 µm wide and 30 µm high, or situated on the top of the ascomata; brown, finely verruculose, 1–4-septate, subcylindrical, straight to geniculate–sinuous, unbranched,	20–40 × 2–4 µm.	aggregated arising from the upper cells of an irregular brown stroma	up to 30 µm.
**Conidiogenous Cells**	terminal, discrete, brown, verruculose, subcylindrical or doliiform, with 1–5 inconspicuous percurrent proliferations, or at times with sympodial proliferation	8–15 × 4–6 µm	lack the asexual state		terminal, unbranched, medium brown, smooth to verruculose, tapering to the flattipped apical loci, proliferating sympodially	7–15 × 2–3 µm	terminal, unbranched, medium brown, smooth to verruculose,	9.3 (7.4–11.2) × 5.0 (3.9–6.0) µm.
**Conidia**	solitary, exuded in cirri, subcylindrical to obclavate, apex obtuse, base obconically truncate, widest in middle or basal third of conidium, thick-walled	60–80 × 5–6 µm	lack the asexual state		solitary, or in simple chains, medium brown, verruculose, subcylindrical to ellipsoidal, apex obtuse, base subtruncate, 1–2-septate, frequently constricted at the septa	7–15 × 3–3.5 µm	solitary, or in simple chains, brown, verruculose, subcylindrical to oval, apex obtuse, base subtruncate, 1–2 septate, frequently constricted at the septa,	8.8 (7.0–13.7) × 4.7 (3.8–6.6) µm
**Culture characteristics**	Colonies pale white (surface), and pale mouse-grey (reverse), with smooth, slightly irregular margins, obtaining 13 mm diam after 14 d at 25°C in the dark on MEA		Growth of single-spore cultures on malt-extract agar at 25° was relatively fast with cultures reaching 60–70 mm diam. in 2 months. Aerial hyphae in the centre of cultures was whitish grey and slightly raised, becoming flat, light grey to dark grey and finally pink-grey at the irregular outer edge. Cultures were dark grey to reddish black on the reverse. If cultures are left for more than 2 months central hyphae becomes pink and apinkish brown pigment develops in the media.		Colonies on MEA reaching 18–30 mm diam after 3 wk; colonies erumpent, folding, margin smooth, irregular, aerial mycelium moderate, pale olivaceousgrey; reverse iron-grey; on PDA with moderate aerial mycelium, olivaceous-grey with patches of pale olivaceous-grey; reverse olivaceous-black; on OA pale olivaceous-grey with patches of olivaceous-grey and iron-grey.		Mycelium consisting of septate, branched, verruculose hyphae, 2–3 µm wide, in some points with red pigment inside, due the presence of cercosporin and isocercosporin. Colonies spreading, erumpent, lobed margins and moderate aerial mycelium, reaching 27 mm after 14 days at 28 ºC. On PDA medium, the surface is olivaceus-grey with vinaceus margin, the reverse side showed the same characteristics however red due to a diffuse pigment. On OA surface olivaceus-grey with yellow center, and on MEA green-grey and reverse iron-grey	
**Material examined**	Brazil, Belém, on leaves of *Eugenia klotzschiana*		Nowa Nowa, on leaves of *Eucalyptus grandis*		Colombia, Angela Maria, on leaves of *Eucalyptus urophylla*,		Brazil, Pantanal, Mato Grosso do Sul, on leaves of *Vochysia divergens*	
